# Supplementary material for: The impact of locomotion on the brain evolution of squirrels and close relatives
Source: Commun Biol. 2021 Apr 12;4:460. doi: 10.1038/s42003-021-01887-8 (PMC8042109; doi:10.1038/s42003-021-01887-8)
Supplement: Supplementary file 2 — Supplementary Information [file 42003_2021_1887_MOESM2_ESM.pdf]

Supplementary Figures, Tables and Code

The impact of locomotion on the brain evolution of squirrels and close relatives

Ornella C. Bertrand[1], Hans P. Püschel[1], Julia A. Schwab[1], Mary T. Silcox[2] and Stephen L. Brusatte[1]

[1]School of GeoSciences, University of Edinburgh, Grant Institute, Edinburgh, Scotland, UK

[2]Dept. of Anthropology, University of Toronto Scarborough, 1265 Military Trail Scarborough, Toronto, ON, Canada M1C 1A4

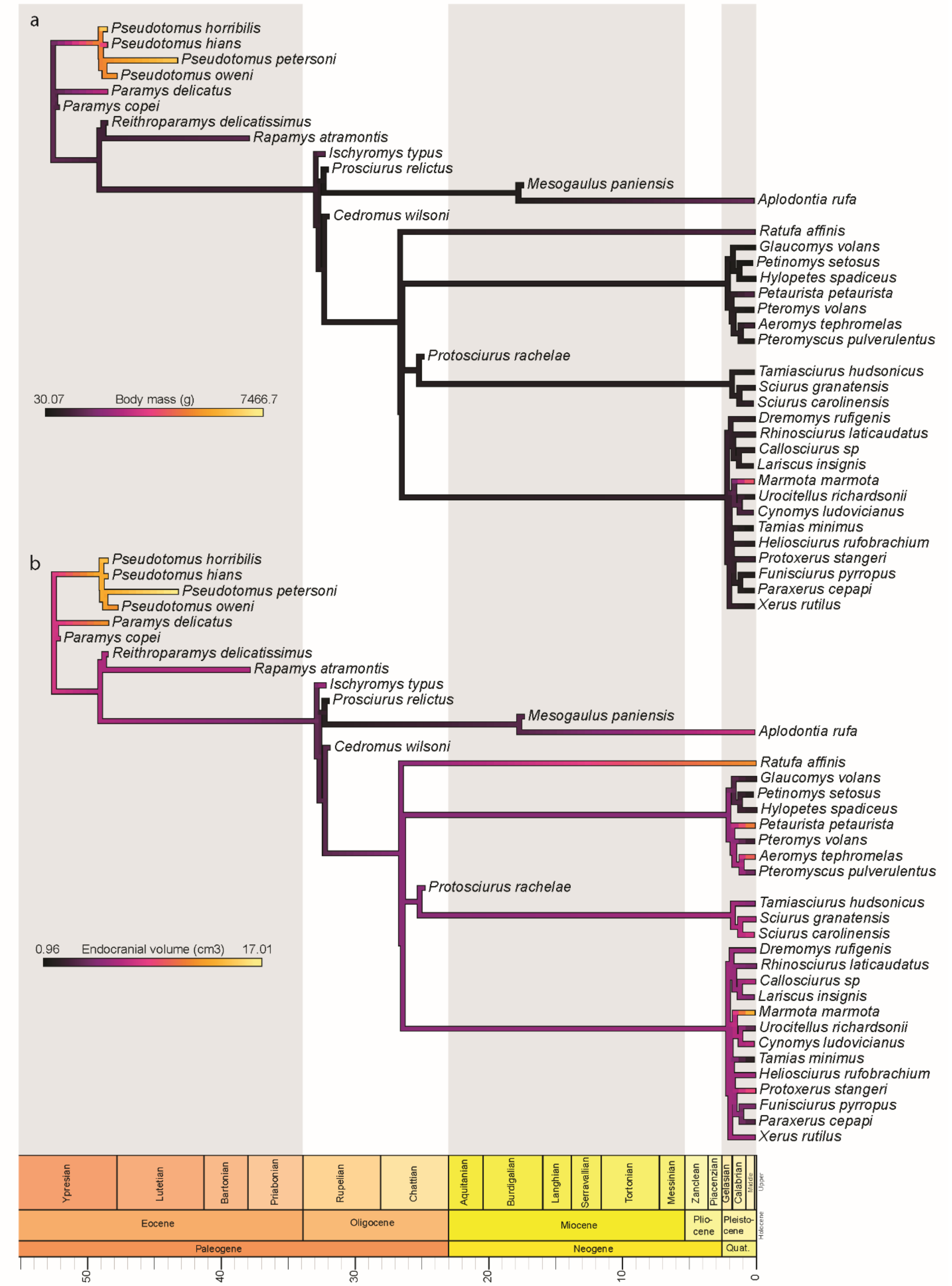

Supplementary Fig. 1 Ancestral state reconstructions with a geological timescale. **a.** Body mass in g; **b.** Endocranial volume (cm^3). Values are nodes are available in Table S4.

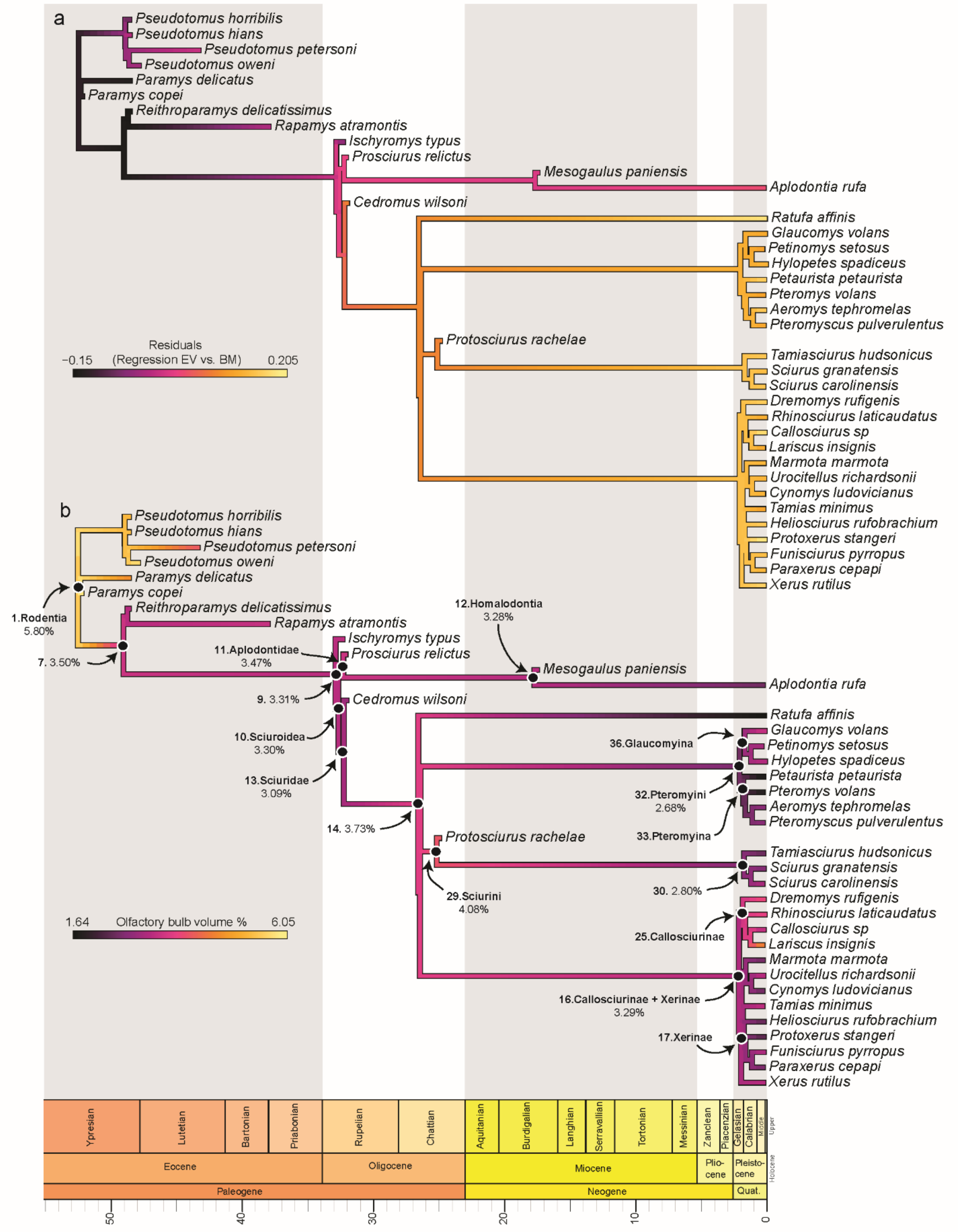

Supplementary Fig. 2 Ancestral state reconstructions with a geological timescale. **a.** Residuals from the regression Log Endocranial volume against Body mass; **b.** Olfactory bulb volume percentage. **Abbreviations:** BM, Body mass; EV, Endocranial volume.

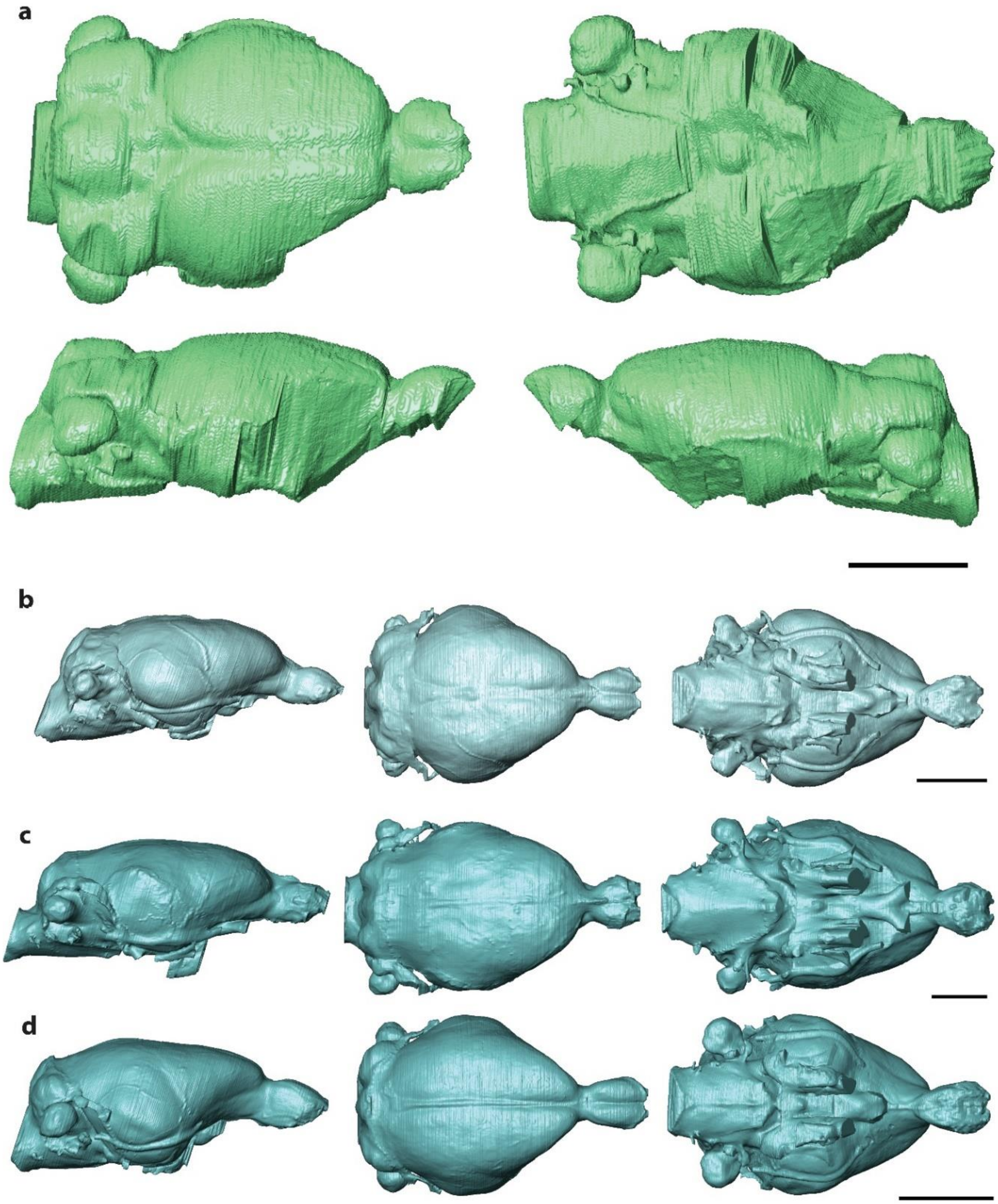

Supplementary Fig. 3 Modified and new virtual endocasts. **a.** Virtual endocast of *Reithroparamys delicatissimus* (AMNH 12561) obtained after re-segmentation of the front part of the endocast (previous version in Bertrand et al.[1]); **b.** *Cynomys ludovicianus* (AMNH114522); **c.** *Marmota marmota* (AMNH 146619); **d.** *Urocitellus richardsonii* (AMNH 15062). Scale = 10 mm.

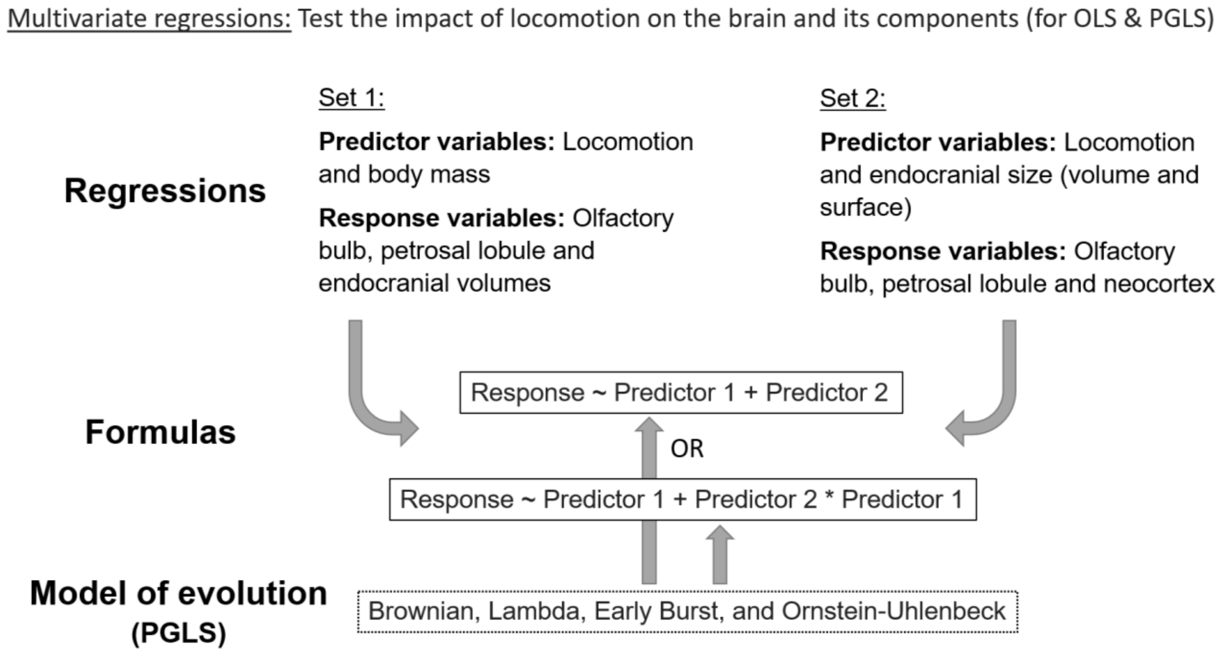

Supplementary Fig. 4 Summary of the steps for the multivariate regression analyses. For each regression, one of the formulas and model of evolution are chosen over the others using the lowest AIC value. We obtained a total of six regressions (three from Set 1 and three from Set 2).

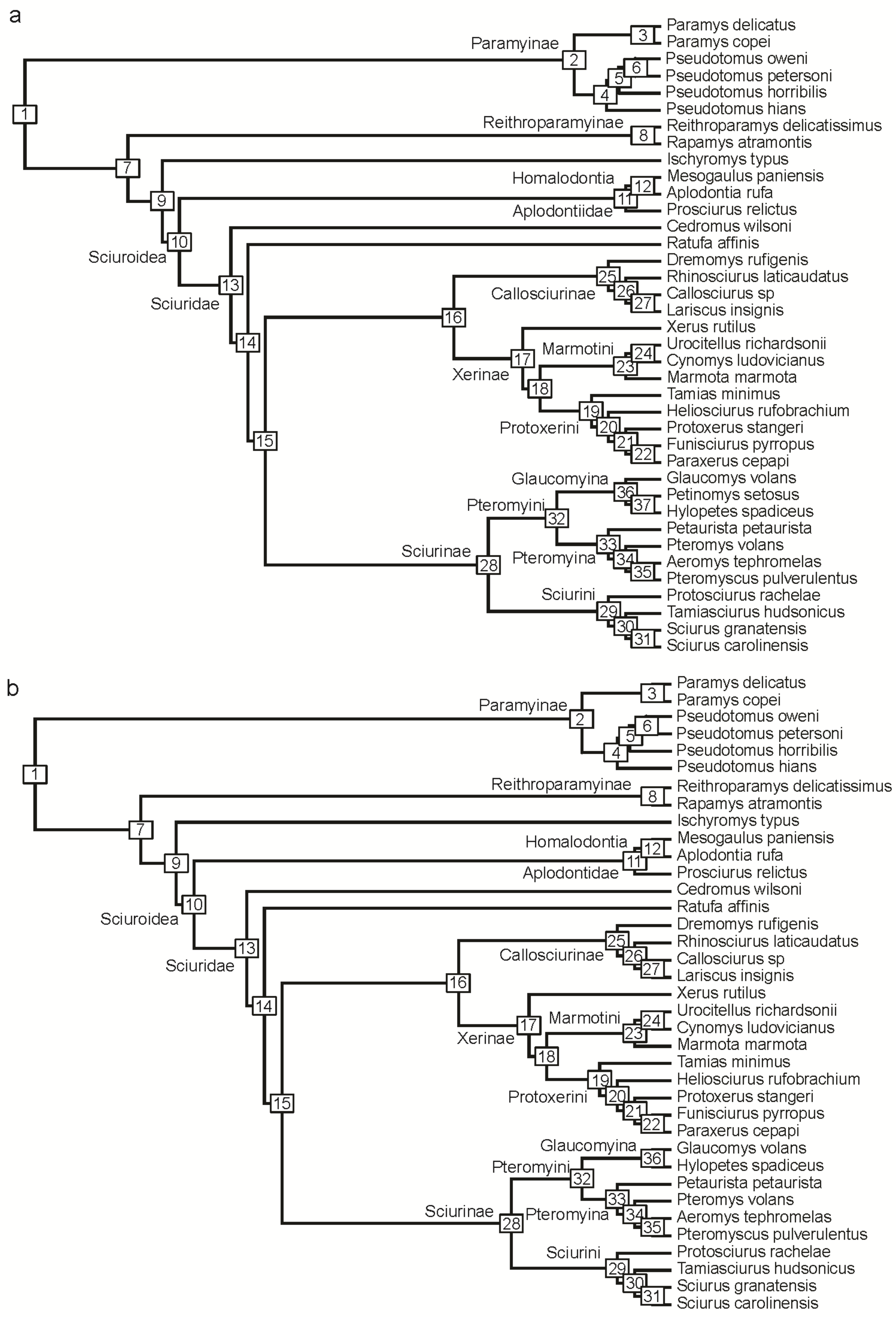

Supplementary Fig. 5 Cladograms with node numbers. **a.** including *Petinomys setosus* (USNM 488674), and **b.** without *Petinomys setosus* for the petrosal lobule reconstruction only.

Supplementary Table 1. Results from the non-phylogenetic corrected ANOVA using Residual Randomization. **Abbreviations:** 1.EB, Endocranial volume ~ Body mass + Locomotion * Body mass; 1.PB, Petrosal lobule volume ~ Body mass + Locomotion*Body mass; 2.PE, Petrosal lobule volume ~ Endocranial volume + Locomotion; 2.NE, Neocortex surface area ~ Endocranial surface area + Locomotion; 1.OB, Olfactory bulb volume ~ Body mass + Locomotion; 2.OE, Olfactory bulb volume ~ Endocranial volume + Locomotion; Df, degrees of freedom; MS, Mean squares; SS, Sum of squares; R2, coefficient of determination.

**Regression**		**Df**	**SS**	**MS**	**R2**	**F**	**Z**	**Pr(>F)**
1.EB	Body mass	1	3.126	3.126	0.891	855.504	3.626	**0.001**
	Locomotion	4	0.169	0.042	0.048	11.555	3.1313	**0.001**
	Body mass * Locomotion	4	0.1119	0.02798	0.03189	7.6565	2.8044	**0.001**
	Residuals	28	0.1023	0.00365	0.02916			
	Total	37	3.509					
1.PB	Body mass	1	0.894	0.894	0.361	55.196	2.322	**0.001**
	Locomotion	4	0.812	0.203	0.328	12.543	3.169	**0.001**
	Body mass * Locomotion	4	0.33514	0.08378	0.13522	5.1743	2.1692	**0.004**
	Residuals	27	0.43719	0.01619	0.17639			
	Total	36	2.47851					
2.PE	Endocranial volume	1	1.401	1.401	0.565	76.141	2.412	**0.001**
	Locomotion	4	0.507	0.127	0.205	6.891	2.5979	**0.001**
	Residuals	31	0.57039	0.0184	0.23013			
	Total	36	2.47851					
2.NE	Endocranial volume	1	1.064	1.064	0.763	183.742	2.841	**0.001**
	Locomotion	4	0.145	0.036	0.104	6.276	2.4975	**0.004**
	Residuals	32	0.18525	0.00579	0.13286			
	Total	37	1.39425					
1.OB	Body mass	1	3.965	3.965	0.879	247.811	2.977	**0.001**
	Locomotion	4	0.033	0.008	0.007	0.523	-0.5232	0.725
	Residuals	32	0.512	0.016	0.11352			
	Total	37	4.51					
2.OE	Endocranial volume	1	3.831	3.831	0.849	260.108	3.035	**0.001**
	Locomotion	4	0.208	0.052	0.046	3.524	1.6933	**0.019**
	Residuals	32	0.4713	0.0147	0.10451			
	Total	37	4.51					

Supplementary Table 2. Ancestral state reconstruction for the different variables with associated node numbers (see Supplementary Fig. 5). The nodes 5 and 6 are the resolved nodes estimated during the generation of the calibrated tree, *Pseudotomus oweni* and *P. petersoni* are considered to the most derived (see ancestral reconstructed figures).

Node names	Node #	PEQ	Residuals	Body mass (g)	Brain volume (cm3)	Brain surface (mm2)	Neocortex surface (mm2)	Petrosal lobule volume (mm3)	Olfactory bulb volume (mm3)	Neocortex surface area percentage (%)	Petrosal lobule percentage (%)	Petrosal lobule percentage (%) Body mass	Olfactory bulb percentage (%)	Olfactory bulb percentage (%) Body mass
**Rodentia**	1	0.39	-0.12	1356.64	8.03	3530.17	647.56	98.34	463.96	18.28	1.25	0.01	5.80	0.04
**Paramyinae**	2	0.39	-0.12	1356.64	8.03	3530.17	647.56	98.34	463.96	18.28	1.25	0.01	5.8	0.04
	3	0.39	-0.13	1192.71	7.82	3465.08	630.47	93.99	461.33	18.18	1.22	0.01	5.93	0.04
Pseudotomus	4	0.33	-0.05	5238.47	13.97	5235.84	1091.65	144.77	756.44	20.99	1.03	0.00	5.44	0.02
	5	0.33	-0.04	5060.94	13.9	5193.17	1095.16	142.42	754.02	21.23	1.02	0.00	5.45	0.02
	6	0.32	-0.04	5196.51	13.56	5107.17	1089.89	123.44	743.38	21.45	0.90	0.00	5.52	0.01
	7	0.37	-0.14	912.3	6.55	3193.89	562.92	141.6	232.9	17.69	2.19	0.02	3.50	0.02
**Reithroparamyinae**	8	0.37	-0.15	877.11	6.47	3184.67	556.35	146.37	214.55	17.49	2.27	0.02	3.3	0.02
	9	0.32	0.00	724.94	4.21	2117.15	519.87	87.68	138.53	26.00	2.48	0.04	3.31	0.05
**Sciuroidea**	10	0.34	0.02	486.64	3.40	1829.14	486.13	80.83	110.33	27.86	2.79	0.06	3.30	0.06
**Aplodontiidae**	11	0.33	0.03	169.58	1.72	1148.38	323.44	46.88	57.63	29.11	3.17	0.09	3.47	0.09
**Homalodontia**	12	0.37	0.01	278.74	3.50	1639.76	439.81	46.48	114.24	26.83	1.35	0.02	3.28	0.04
**Sciuridae**	13	0.37	0.07	342.73	3.58	1981.9	595.1	103.95	109.97	30.32	3.03	0.05	3.09	0.05
	14	0.46	0.10	394.54	5.15	2241.02	714.85	145.8	186.38	31.79	2.92	0.05	3.73	0.06
	15	0.46	0.10	392.29	5.15	2239.59	714.15	146.34	188.12	31.79	2.92	0.05	3.76	0.06
	16	0.47	0.16	716.56	6.07	2251.02	822.13	103.2	189.78	36.69	1.87	0.03	3.29	0.05
**Xerinae**	17	0.47	0.16	778.66	6.2	2278.81	831.3	100.46	184.53	36.72	1.81	0.03	3.14	0.05
	18	0.47	0.16	804.97	6.23	2286.65	832.78	99.48	182.89	36.68	1.79	0.03	3.1	0.05
**Protoxerini**	19	0.48	0.16	706.02	5.97	2207.68	809.29	98.37	171.75	36.83	1.83	0.03	3.04	0.06
	20	0.49	0.16	669.25	5.99	2213.67	815.24	100.5	169.65	36.94	1.83	0.03	2.99	0.05
	21	0.49	0.16	625.78	6.02	2220.19	823.63	103.43	167.39	37.11	1.83	0.03	2.93	0.05
	22	0.48	0.16	408.3	4.83	1920.57	710.94	86.33	138.09	37.01	1.82	0.03	2.92	0.05
**Marmotini**	23	0.43	0.15	1315.32	7.35	2594.17	917.8	95.49	205.37	35.93	1.55	0.02	2.98	0.04
	24	0.41	0.15	970.53	6.34	2360.53	835.62	79.91	176.41	35.8	1.45	0.02	2.95	0.04
**Callosciurinae**	25	0.47	0.16	614.64	5.87	2203.9	807.77	107.38	198.72	36.71	1.96	0.03	3.53	0.05
	26	0.47	0.16	567.57	5.73	2173.83	795.75	108.12	200.95	36.58	2.00	0.03	3.64	0.05
	27	0.49	0.16	479.63	5.81	2197.03	809.59	112.78	214.53	36.72	2.03	0.03	3.83	0.06
**Sciurinae**	28	0.46	0.10	391.39	5.15	2239.25	713.86	146.52	188.51	31.79	2.93	0.05	3.77	0.06
**Sciurini**	29	0.47	0.09	355.86	5.12	2245.45	704.29	156.99	208.03	31.35	3.08	0.05	4.08	0.06
	30	0.57	0.16	384.62	6.36	2322.91	848.57	137.45	181.23	36.58	2.19	0.04	2.8	0.05
	31	0.58	0.16	431.58	6.83	2440.05	887.1	143.3	199.55	36.38	2.12	0.04	2.88	0.05
**Pteromyini**	32	0.49	0.14	358.77	5.03	1848.31	667.65	81.54	117.91	36.24	1.46	0.03	2.68	0.06
**Pteromyina**	33	0.51	0.14	446.18	5.96	2100.59	757.04	94.52	133.62	36.05	1.49	0.03	2.48	0.05
	34	0.5	0.14	428.59	5.83	2078.91	747.7	91.07	135.12	35.93	1.49	0.03	2.46	0.05
	35	0.53	0.15	493.12	6.75	2348.84	841.37	99.87	178.28	35.67	1.44	0.03	2.67	0.05
**Glaucomyina**	36	0.47	0.14	238.63	3.75	1496.77	544.58	62.72	95.27	36.57	1.39	0.03	2.93	0.08
	37	0.45	0.13	149.23	2.76	1218.28	447.43	-	76.03	36.87	-	-	3.06	0.09

Supplementary Table 3. Settings of the CT scanner for the tree new scans. The interslice distance is different for several endocasts because only every second slice was used to build the endocast; consequently, the interslice distance was doubled for these specific cases.

Subfamily	Species	Collection number	Source-object Distance (mm)	Energy settings		Interslice spacing /interpixel distance (mm)	Interslice distance (endocast) (mm)
				kv	uA		
Paramyinae	*Paramys copei*	AMNH 4756	158.79	170.0	180.0	0.03905	-
Paramyinae	*Paramys delicatus*	AMNH 12506	144.74	170.0	180.0	0.03559	-
Paramyinae	*Pseudotomus horribilis*	USNM 17159	318.52	220.0	194.0	0.06304	-
Paramyinae	*Pseudotomus oweni*	USNM 17161	265.08	165.0	88.0	0.05695	-
Paramyinae	*Pseudotomus petersoni*	AMNH 2018	313.45	140.0	240.0	0.07708	-
Paramyinae	*Pseudotomus hians*	AMNH 5025	214.79	190.0	220.0	0.05289	-
Reithroparamyinae	*Reithroparamys delicatissimus*	AMNH 12561	176.94	180.0	240.0	0.04351	-
Reithroparamyinae	*Rapamys atramontis*	AMNH 128706	121.34	160.0	180.0	0.02984	-
Reithroparamyinae	*Rapamys atramontis*	AMNH 128704	175.87	170.0	180.0	0.04325	-
Ischyromyinae	*Ischyromys typus*	ROMV 1007	229.08	155.0	186.0	0.03946	-
Ischyromyinae	*Ischyromys typus*	AMNH 12252	90.67	170.0	100.0	0.02291	-
Ischyromyinae	*Ischyromys typus*	AMNH F: AM 144638	166.75	170.0	220.0	0.04106	-
Cedromurinae	*Cedromus wilsoni*	USNM 256584	113.87	165.0	88.0	0.02613	-
Sciurinae (Sciurini)	*Protosciurus* cf. *rachelae*	YPM 14736	111.98	170.0	100.0	0.02829	-
Sciurinae (Sciurini)	*Protosciurus* cf. *rachelae*	YPM 14737	106.87	170.0	100.0	0.02700	-
Sciurinae (Sciurini)	*Sciurus carolinensis*	AMNH 258346	187.80	120.0	175.0	0.03235	-
Sciurinae (Sciurini)	*Sciurus granatensis*	USNM 441999	131.12	150.0	98.0	0.02823	-
Sciurinae (Sciurini)	*Tamiasciurus hudsonicus*	USNM 549146	119.96	150.0	98.0	0.02583	-
Sciurinae (Pteromyini)	*Aeromys tephromelas*	USNM 481190	170.24	225.0	55.0	0.03689	-
Sciurinae (Pteromyini)	*Glaucomys volans*	AMNH 240290	107.46	105.0	165.0	0.01851	-
Sciurinae (Pteromyini)	*Petaurista petaurista*	USNM 589079	170.24	225.0	55.0	0.03689	-
Sciurinae (Pteromyini)	*Hylopetes spadiceus*	USNM 488639	93.89	160.0	119.0	0.02021	-
Sciurinae (Pteromyini)	*Petinomys setosus*	USNM 488674	82.87	150.0	98.0	0.01784	-
Sciurinae (Pteromyini)	*Pteromyscus pulverulentus*	USNM 481178	113.53	150.0	98.0	0.02444	-
Sciurinae (Pteromyini)	*Pteromys volans*	USNM 172622	96.69	160.0	119.0	0.02082	-
Xerinae	*Tamias minimus*	USNM 298500	74.42	160.0	119.0	0.01602	-
Xerinae	*Funisciurus pyrropus*	USNM 294865	122.09	255.0	55.0	0.02645	-
Xerinae	*Heliosciurus rufobrachium*	USNM 378091	130.89	225.0	55.0	0.02836	-
Xerinae	*Paraxerus cepapi*	USNM 367956	102.24	150.0	98.0	0.02201	-
Xerinae	*Protoxerus stangeri*	USNM 435027	158.79	225.0	55.0	0.03440	-
Xerinae	*Xerus rutilus*	AMNH 179092	67.16	60.4	0.1	0.03500	-
Xerinae	*Marmota marmota*	AMNH 146619	90.06	60.4	82.1	0.04003	0.08005
Xerinae	*Cynomys ludovicianus*	AMNH114522	134.75	60.6	82.1	0.04902	-
Xerinae	*Urocitellus richardsonii*	AMNH 15062	66.37	60.4	80.5	0.03501	-
Callosciurinae	*Rhinosciurus laticaudatus*	USNM 488511	143.25	150.0	98.0	0.03084	-
Callosciurinae	*Callosciurus* sp.	USNM 294865	144.07	150.0	98.0	0.03102	-
Callosciurinae	*Lariscus insignis*	USNM 488570	132.25	150.0	980.0	0.02847	-
Callosciurinae	*Dremomys rufigenis*	USNM 488602	140.03	150.0	98.0	0.03015	-
Ratufinae	*Ratufa affinis*	USNM 488104	170.24	225.0	55.0	0.03689	0.07400
Aplodontinae	*Aplodontia rufa*	AMNH 42389	239.82	145.0	160.0	0.04131	0.08263
Mylagaulinae	*Mesogaulus paniensis*	AMNH F:AM 65511	196.29	220.0	150.0	0.04833	-
Prosciurinae	*Prosciurus relictus*	USNM 437793	77.34	165.0	98.0	0.01619	-

Supplementary Table 4. Sample of rodents used for the different analyses with associated body mass based on cranial length, status and locomotor behaviour. The cranial length of AMNH 128706, ROMV 1007, YPM 14736 were used to determine the body mass of *Rapamys atramontis*, *Ischyromys typus*, and *Protosciurus* cf. *rachelae* respectively.

**Subfamily**	**Species**	**Specimens**	**Status**	**Body mass (g)**	**Locomotion**	**References (locomotion)**
Paramyinae	*Paramys copei*	AMNH 4756	Extinct	1029.89	Scansorial	2
Paramyinae	*Paramys delicatus*	AMNH 12506	Extinct	2913.82	Scansorial	2
Paramyinae	*Pseudotomus horribilis*	USNM 17159	Extinct	7466.70	Fossorial	3, 4
Paramyinae	*Pseudotomus oweni*	USNM 17161	Extinct	5396.00	Fossorial	3, 4
Paramyinae	*Pseudotomus petersoni*	AMNH 2018	Extinct	6644.56	Fossorial	3, 4
Paramyinae	*Pseudotomus hians*	AMNH 5025	Extinct	3153.50	Fossorial	3, 4
Reithroparamyinae	*Reithroparamys delicatissimus*	AMNH 12561	Extinct	856.23	Scansorial	2, 3
Reithroparamyinae	*Rapamys atramontis*	AMNH 128706; AMNH 128704	Extinct	1307.61	Scansorial	7
Ischyromyinae	*Ischyromys typus*	ROMV 1007; AMNH 12252; AMNH F: AM 144638	Extinct	1342.23	Fossorial	6
Cedromurinae	*Cedromus wilsoni*	USNM 256584	Extinct	268.89	Arboreal	5
Sciurinae (Sciurini)	*Protosciurus* cf. *rachelae*	YPM 14736; YPM 14737	Extinct	349.62	Arboreal	7
Sciurinae (Sciurini)	*Sciurus carolinensis*	AMNH 258346	Extant	592.55	Arboreal	8
Sciurinae (Sciurini)	*Sciurus granatensis*	USNM 441999	Extant	336.99	Arboreal	9, 10, 11
Sciurinae (Sciurini)	*Tamiasciurus hudsonicus*	USNM 549146	Extant	256.61	Arboreal	8
Xerinae	*Tamias minimus*	USNM 298500	Extant	37.05	Scansorial	8
Xerinae	*Funisciurus pyrropus*	USNM 294865	Extant	301.15	Scansorial	12
Xerinae	*Heliosciurus rufobrachium*	USNM 378091	Extant	354.98	Arboreal	12
Xerinae	*Paraxerus cepapi*	USNM 367956	Extant	138.13	Scansorial	12
Xerinae	*Protoxerus stangeri*	USNM 435027	Extant	767.23	Arboreal	12, 13
Xerinae	*Xerus rutilus*	AMNH 179092	Extant	353.90	Terrestrial	12, 13
Xerinae	*Marmota marmota*	AMNH 146619	Extant	4546.97	Terrestrial	14
Xerinae	*Cynomys ludovicianus*	AMNH114522	Extant	938.70	Terrestrial	14
Xerinae	*Urocitellus richardsonii*	AMNH 15062	Extant	245.78	Terrestrial	14
Sciurinae (Pteromyini)	*Aeromys tephromelas*	USNM 481190	Extant	904.59	Glider	13
Sciurinae (Pteromyini)	*Glaucomys volans*	AMNH 240290	Extant	63.97	Glider	8
Sciurinae (Pteromyini)	*Petaurista petaurista*	USNM 589079	Extant	1096.65	Glider	15, 16, 13
Sciurinae (Pteromyini)	*Hylopetes spadiceus*	USNM 488639	Extant	84.22	Glider	14
Sciurinae (Pteromyini)	*Petinomys setosus*	USNM 488674	Extant	41.86	Glider	14
Sciurinae (Pteromyini)	*Pteromyscus pulverulentus*	USNM 481178	Extant	195.44	Glider	14
Sciurinae (Pteromyini)	*Pteromys volans*	USNM 172622	Extant	106.37	Glider	13
Callosciurinae	*Rhinosciurus laticaudatus*	USNM 488511	Extant	507.38	Terrestrial	14, 15
Callosciurinae	*Callosciurus* sp.	USNM 294865	Extant	437.35	Arboreal	14
Callosciurinae	*Lariscus insignis*	USNM 488570	Extant	324.71	Terrestrial	15, 13
Callosciurinae	*Dremomys rufigenis*	USNM 488602	Extant	418.43	Scansorial	15, 14
Ratufinae	*Ratufa affinis*	USNM 488104	Extant	1074.27	Arboreal	13
Aplodontinae	*Aplodontia rufa*	AMNH 42389	Extant	1475.86	Fossorial	17
Mylagaulinae	*Mesogaulus paniensis*	AMNH F:AM 65511	Extinct	266.48	Fossorial	17
Prosciurinae	*Prosciurus relictus*	USNM 437793	Extinct	30.07	Arboreal	5

Supplementary Table 5. Dataset based on various studies and new specimens (see material and method section for more information). PEQ values were determined using the PGLS equation of the regression Endocranial volume vs. Body mass. Newly generated data are in blue. **Abbreviation:** PEQ, phylogenetic encephalization quotient.

Subfamily/tribe	Species	Collection number	Body mass (g)	Brain volume (cm3)	Brain surface (mm2)	Neocortex surface (mm2)	Petrosal lobule volume (mm3)	Olfactory bulb volume (mm3)	Neocortex surface area percentage (%)	Petrosal lobule percentage (%)	Petrosal lobule percentage (%) Body mass	Olfactory bulb percentage (%)	Olfactory bulb percentage (%) Body mass	Predicted (Brain vs. Body size)	Residuals (Brain vs. Body size)	PEQ	Locomotion
Paramyinae	*Paramys_copei*	AMNH 4756	1029.89	7.53	3378.48	612.69	89.99	455.45	18.14	1.20	0.01	6.05	0.04	2.92	-0.13	0.39	Scansorial
Paramyinae	*Paramys_delicatus*	AMNH 12506	2913.82	12.57	4864.86	836.31	129.30	595.51	17.19	1.03	0.00	4.74	0.02	3.06	-0.14	0.38	Scansorial
Paramyinae	*Pseudotomus_horribilis*	USNM 17159	7466.70	15.19	5842.80	1095.61	173.80	808.92	18.75	1.14	0.00	5.33	0.01	3.13	-0.09	0.28	Fossorial
Paramyinae	*Pseudotomus_oweni*	USNM 17161	5396.00	12.06	4744.38	1038.73	74.80	717.06	21.89	0.62	0.00	5.94	0.01	3.05	-0.03	0.26	Fossorial
Paramyinae	*Pseudotomus_petersoni*	AMNH 2018	6644.56	17.01	5843.70	1337.58	65.80	704.34	22.89	0.39	0.00	4.14	0.01	3.13	0.00	0.33	Fossorial
Paramyinae	*Pseudotomus_hians*	AMNH 5025	3153.50	13.68	4878.63	1136.08	142.40	743.20	23.29	1.04	0.00	5.43	0.02	3.06	0.00	0.40	Fossorial
Reithroparamyinae	*Reithroparamys_delicatissimus*	AMNH 12561	856.23	**6.43**	**3178.90**	**550.88**	148.86	**206.31**	**17.33**	**2.31**	**0.02**	**3.21**	**0.02**	2.89	-0.15	0.37	Scansorial
Reithroparamyinae	*Rapamys_atramontis*	AMNH 128706; AMNH 128704	1307.61	6.56	3321.41	763.65	115.56	225.34	22.99	1.76	0.01	3.44	0.02	2.91	-0.03	0.30	Scansorial
Ischyromyinae	*Ischyromys_typus*	ROMV 1007; AMNH 12252; AMNH F:AM 144638	1342.23	6.26	2835.36	606.72	103.85	209.22	21.40	1.66	0.01	3.34	0.02	2.85	-0.06	0.29	Fossorial
Cedromurinae	*Cedromus_wilsoni*	USNM 256584	268.89	3.61	2048.70	645.15	113.96	106.97	31.49	3.16	0.04	2.96	0.04	2.72	0.09	0.39	Arboreal
Sciurini	*Protosciurus_rachelae*	YPM 14736; YPM 14737	349.62	5.10	2245.80	701.39	158.93	211.55	31.23	3.11	0.05	4.15	0.06	2.75	0.09	0.47	Arboreal
Sciurini	*Sciurus_carolinensis*	AMNH 258346	592.55	8.05	2720.59	990.64	163.26	255.92	36.41	2.03	0.03	3.18	0.04	2.83	0.17	0.57	Arboreal
Sciurini	*Sciurus_granatensis*	USNM 441999	336.99	6.32	2340.36	841.62	131.63	169.88	35.96	2.08	0.04	2.69	0.05	2.77	0.15	0.60	Arboreal
Sciurini	*Tamiasciurus_hudsonicus*	USNM 549146	256.61	5.15	2003.97	751.24	120.02	128.89	37.49	2.33	0.05	2.50	0.05	2.71	0.17	0.56	Arboreal
Xerinae	*Tamias_minimus*	USNM 298500	37.05	1.52	890.54	325.31	34.80	51.19	36.53	2.29	0.09	3.36	0.14	2.39	0.12	0.46	Scansorial
Xerinae	*Funisciurus_pyrropus*	USNM 294865	301.15	4.55	1881.42	695.25	99.54	146.78	36.95	2.19	0.03	3.22	0.05	2.68	0.16	0.46	Scansorial
Xerinae	*Heliosciurus_rufobrachium*	USNM 378091	354.98	6.08	2271.66	850.16	113.61	149.15	37.42	1.87	0.03	2.45	0.04	2.76	0.17	0.56	Arboreal
Xerinae	*Paraxerus_cepapi*	USNM 367956	138.13	3.05	1445.98	533.37	44.66	79.88	36.89	1.46	0.03	2.62	0.06	2.58	0.15	0.46	Scansorial
Xerinae	*Protoxerus_stangeri*	USNM 435027	767.23	9.75	3138.06	1233.50	183.74	225.57	39.31	1.89	0.02	2.31	0.03	2.89	0.20	0.60	Arboreal
Xerinae	*Xerus_rutilus*	AMNH 179092	**353.90**	**6.00**	**2249.69**	**866.66**	**111.95**	**196.97**	**38.52**	**1.87**	**0.03**	**3.28**	**0.06**	2.76	0.18	0.55	Terrestrial
Xerinae	*Marmota_marmota*	AMNH 146619	**4546.97**	**15.20**	**4622.40**	**1533.62**	**127.40**	**390.88**	**33.18**	**0.84**	**0.00**	**2.57**	**0.01**	3.04	0.15	0.36	Terrestrial
Xerinae	*Cynomys_ludovicianus*	AMNH114522	**938.70**	**7.22**	**2666.16**	**928.61**	**70.97**	**182.73**	**34.83**	**0.98**	**0.01**	**2.53**	**0.02**	2.82	0.15	0.40	Terrestrial
Xerinae	*Urocitellus_richardsonii*	AMNH 15062	**245.78**	**3.16**	**1514.13**	**553.66**	**55.33**	**105.75**	**36.57**	**1.75**	**0.02**	**3.35**	**0.04**	2.60	0.14	0.35	Terrestrial
Pteromyini	*Aeromys_tephromelas*	USNM 481190	904.59	11.46	3528.53	1276.75	166.10	326.77	36.18	1.45	0.02	2.85	0.04	2.93	0.17	0.64	Glider
Pteromyini	*Glaucomys_volans*	AMNH 240290	63.97	2.01	1040.25	383.61	33.73	70.08	36.88	1.68	0.05	3.49	0.11	2.45	0.13	0.46	Glider
Pteromyini	*Petaurista_petaurista*	USNM 589079	1096.65	12.32	3673.24	1331.61	199.90	201.76	36.25	1.62	0.02	1.64	0.02	2.95	0.18	0.62	Glider
Pteromyini	*Hylopetes_spadiceus*	USNM 488639	84.22	2.12	1078.40	410.01	17.93	69.95	38.02	0.85	0.02	3.30	0.08	2.47	0.15	0.42	Glider
Pteromyini	*Petinomys_setosus*	USNM 488674	41.86	1.51	836.52	305.69	NA	46.96	36.54	NA	NA	3.10	0.11	2.37	0.12	0.43	Glider
Pteromyini	*Pteromyscus_pulverulentus*	USNM 481178	195.44	3.62	1627.40	565.88	49.50	101.74	34.77	1.37	0.03	2.81	0.05	2.63	0.13	0.46	Glider
Pteromyini	*Pteromys_volans*	USNM 172622	106.37	2.33	1205.34	427.98	37.53	40.79	35.51	1.61	0.04	1.75	0.04	2.51	0.12	0.41	Glider
Callosciurinae	*Rhinosciurus_laticaudatus*	USNM 488511	507.38	4.38	1851.63	650.59	97.36	170.11	35.14	2.22	0.02	3.88	0.03	2.68	0.13	0.33	Terrestrial
Callosciurinae	*Callosciurus_sp*	USNM 294865	437.35	7.01	2479.31	974.42	123.91	229.77	39.30	1.77	0.03	3.28	0.05	2.79	0.20	0.58	Arboreal
Callosciurinae	*Lariscus_insignis*	USNM 488570	324.71	4.88	1993.80	691.54	113.00	230.63	34.68	2.32	0.03	4.73	0.07	2.71	0.13	0.47	Terrestrial
Callosciurinae	*Dremomys_rufigenis*	USNM 488602	418.43	5.87	2185.23	826.93	125.71	232.42	37.84	2.14	0.03	3.96	0.06	2.74	0.17	0.50	Scansorial
Ratufinae	*Ratufa_affinis*	USNM 488104	1074.27	12.31	3725.22	1407.87	234.90	201.44	37.79	1.91	0.02	1.64	0.02	2.95	0.19	0.63	Arboreal
Aplodontinae	*Aplodontia_rufa*	AMNH 42389	1475.86	7.89	2878.28	779.01	64.41	203.71	27.07	0.82	0.00	2.58	0.01	2.85	0.04	0.34	Fossorial
Mylagaulinae	*Mesogaulus_paniensis*	AMNH F:AM 65511	266.48	3.47	1632.28	437.51	46.27	113.99	26.80	1.33	0.02	3.29	0.04	2.63	0.01	0.37	Fossorial
Prosciurinae	*Prosciurus_relictus*	USNM 437793	30.07	0.96	845.90	**251.16**	32.08	34.00	**29.69**	3.35	0.11	3.55	0.11	2.37	0.03	0.33	Arboreal

Supplementary Table 6. Matrices used to create a time-scaled phylogenetic tree. The full method is described by Bapst[18].

Time intervals	start_time	end_time
Wasatchian7	53	52.1
Bridgerian2	49.25	48.6
Bridgerian3	48.6	47.9
Uintan2	45.3	42.8
Duchesnean	39.8	37.8
Orellan	33.9	32.05
Orellan1	33.1	32.05
Arikareean	28	23.8
Hemingfordian	18.8	17.5
Pleistocene	2.59	0

Taxon	first_int	last_int
Paramys_copei	1	1
Paramys_delicatus	2	2
Pseudotomus_horribilis	2	2
Pseudotomus_oweni	3	3
Pseudotomus_petersoni	4	4
Pseudotomus_hians	2	2
Reithroparamys_delicatissimus	2	2
Rapamys_atramontis	5	5
Ischyromys_typus	6	6
Cedromus_wilsoni	7	7
Protosciurus_rachelae	8	8
Sciurus_carolinensis	10	10
Sciurus_granatensis	10	10
Tamiasciurus_hudsonicus	10	10
Eutamias_minimus	10	10
Funisciurus_pyrropus	10	10
Heliosciurus_rufobrachium	10	10
Paraxerus_cepapi	10	10
Protoxerus_stangeri	10	10
Xerus_rutilus	10	10
Marmota_marmota	10	10
Cynomys_ludovicianus	10	10
Urocitellus_richardsonii	10	10
Aeromys_tephromelas	10	10
Glaucomys_volans	10	10
Petaurista_petaurista	10	10
Hylopetes_spadiceus	10	10
Petinomys_setosus	10	10
Pteromyscus_pulverulentus	10	10
Pteromys_volans	10	10
Rhinosciurus_laticaudatus	10	10
Callosciurus_sp	10	10
Lariscus_insignis	10	10
Dremomys_rufigenis	10	10
Ratufa_affinis	10	10
Aplodontia_rufa	10	10
Mesogaulus_paniensis	9	9
Prosciurus_relictus	7	7

References for supplementary figures and tables

1 Bertrand, O. C., Amador-Mughal, F., Lang, M. M. & Silcox, M. T. New Virtual Endocasts of Eocene Ischyromyidae and Their Relevance in Evaluating Neurological Changes Occurring Through Time in Rodentia. *J. Mamm. Evol.* **26**, 345-371, doi:10.1007/s10914-017-9425-6 (2019).

2 Rose, K. D. & Chinnery, B. J. The Postcranial Skeleton of Early Eocene Rodents. *Bulletin of Carnegie Museum of Natural History* **36**, 211-244, doi:10.2992/0145-9058(2004)36[211:Tpsoee]2.0.Co;2 (2004).

3 Wood, A. E. The early Tertiary rodents of the family Paramyidae. *Transactions of the American Philosophical Society* **52**, 3-261 (1962).

4 Dunn, R. H. & Rasmussen, D. T. Skeletal morphology and locomotor behavior of Pseudotomus eugenei (Rodentia, Paramyinae) from the Uinta Formation, Utah. *J. Vert. Paleontol.* **27**, 987-1006, doi:10.1671/0272-4634(2007)27[987:Smalbo]2.0.Co;2 (2007).

5 Bhagat, R., Bertrand, O. C. & Silcox, M. T. Evolution of arboreality and fossoriality in squirrels and aplodontid rodents: insights from the semicircular canals of fossil rodents. *J. Anat.* (2020).

6 Scott, W. B., Jepsen, G. L. & Wood, A. E. The Mammalian Fauna of the White River Oligocene: Part II. Rodentia. *Transactions of the American Philosophical Society* **28**, 155-269, doi:10.2307/1005501 (1937).

7 Korth, W. W. & Samuels, J. X. New Rodent Material from the John Day Formation (Arikareean, Middle Oligocene to Early Miocene) of Oregon. *Annals of Carnegie Museum* **83**, 19-84, 66 (2015).

8 Reid, F. *A Field Guide to Mammals of North America, North of Mexico*. (Houghton Mifflin, 2006).

9 Emmons, L. & Feer, F. *Neotropical rainforest mammals : a field guide*. (University of Chicago Press, 1990).

10 Eisenberg, J. F. *Mammals of the Neotropics: The Northern Neotropics. Volume 1. Panama, Colombia, Venezuela, Guyana, Suriname, French Guiana*. Vol. 1 (Univ. Chicago Press, 1989).

11 Nitikman, L. Z. Sciurus granatensis. *Mammalian Species*, 1-8, doi:10.2307/3503822 (1985).

12 Kingdon, J. *East African Mammals. An Atlas of Evolution in Africa, Vol. II, Part B (Hares and Rodents)*. (University of Chicago Press, 1974).

13 Thorington, R. W., Koprowski, J. L., Steele, M. A. & Whatton, J. F. *Squirrels of the World*. (Johns Hopkins University Press, 2012).

14 Nowak, R. M. *Walker's Mammals of the World*. Vol. 2 1732 pp (1999).

15 Francis, C. *Field Guide to the Mammals of South-east Asia*. 2nd edn, (Bloomsbury Publishing, 2019).

16 Smith, A. T. & Xie, Y. *Mammals of China*. (Princeton University Press, 2013).

17 Hopkins, S. S. Causes of lineage decline in the Aplodontidae: testing for the influence of physical and biological change. *Palaeogeogr., Palaeoclimatol., Palaeoecol.* **246**, 331-353 (2007).

18 Bapst, D. W. in *Modern Phylogenetic Comparative Methods and Their Application in Evolutionary Biology: Concepts and Practice* (ed László Zsolt Garamszegi) 515-544 (Springer Berlin Heidelberg, 2014).

Supplementary data - Code

The impact of locomotion on the brain evolution of squirrels and close relatives

Ornella C. Bertrand[1], Hans P. Püschel[1], Julia A. Schwab[1], Mary T. Silcox[2] and Stephen L. Brusatte[1]

[1]School of GeoSciences, University of Edinburgh, Grant Institute, Edinburgh, Scotland, UK

[2]Dept. of Anthropology, University of Toronto Scarborough, 1265 Military Trail Scarborough, Toronto, ON, Canada M1C 1A4

Content:

1. Calibrated phylogeny (p. 3)

2. Permutation tests (p. 6)

3. PGLS regression for endocranial volume vs. body mass (p. 15)

4. PGLS regression for olfactory bulb volume vs. body mass (p. 27)

5. PGLS regression for petrosal lobule volume vs. body mass (p. 39)

6. PGLS regression for olfactory bulb vs. endocranial volume (p. 51)

7. PGLS regression for petrosal lobule vs. endocranial volume (p. 63)

8. PGLS regression for neocortical surface area vs. endocranial surface area (p. 76)

9. Ancestral state reconstruction (p. 89)

```r
#1. Calibrated phylogeny

library("paleotree")

## Loading required package: ape

library("phytools")

## Loading required package: maps

#directory
setwd("~/Desktop/Squirrel_June_8_2020/Code")

#Import tree
tree_squirrel<-read.nexus("squirrel-tree_Meng.nex")

#### bin_cal3TimePaleoPhy method

#taxa range for age
taxon.times<-read.csv("taxon_times3.csv",row.names=1)
int.times<-read.csv("int_times3.csv",row.names=1)

squirrel.range<-list(int.times,taxon.times)

#bin_cal3TimePaleoPhy method
likFun<-make_durationFreqDisc(squirrel.range)

spRes<-optim(parInit(likFun),likFun,lower=parLower(likFun),upper=parUpper(lik
Fun),
             method="L-BFGS-B",control=list(maxit=1000000))

#sampling PROBABILITY per bin
sProb <- spRes[[1]][2]

#calculate meanInt. We need to use an average int.length (intervals not the s
ame duration)
intLength<--apply(squirrel.range[[1]],1,diff)
hist(intLength)
```

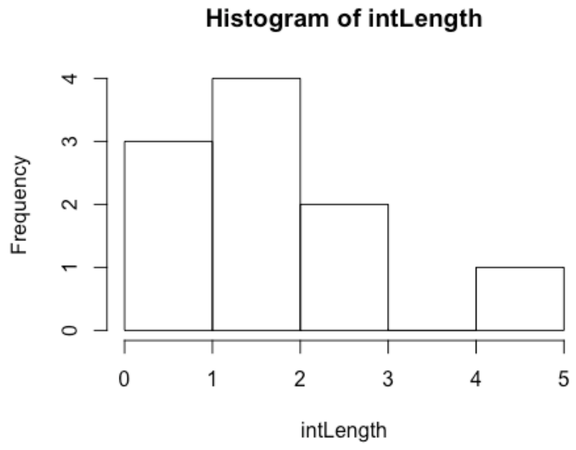


```
meanInt <--mean(apply(squirrel.range[[1]], 1, diff)) #close to 1.8 Million ye
ars

sRate <- sProb2sRate(sProb,int.length = meanInt)
# we also need extinction rate and branching rate (see above)
# need to divide by int.length...

divRate <- spRes[[1]][1]/meanInt

#calibrated tree
tree_squirrel1 <- bin_cal3TimePaleoPhy(tree_squirrel, squirrel.range,brRate =
divRate, extRate = divRate,
                                        sampRate = sRate, ntrees = 100, plot =
FALSE)
multiDiv(tree_squirrel1)
```

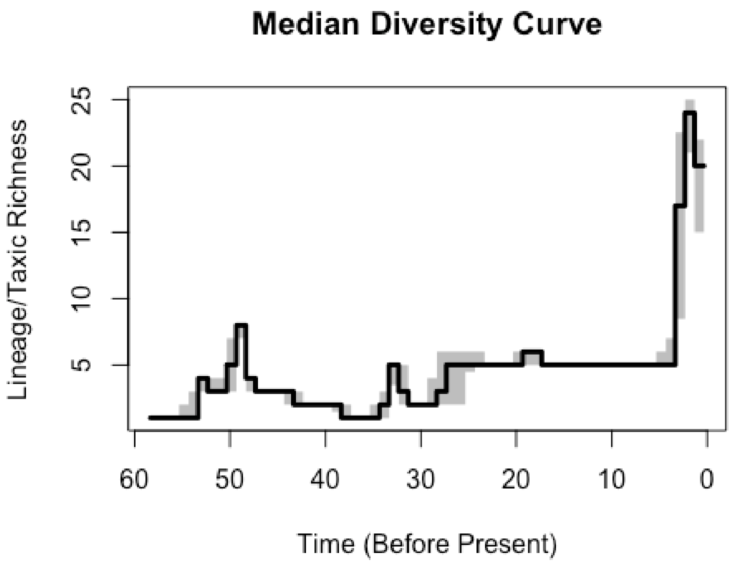

```r
#Average tree - takes about ~7 mins
tree_squirrel3<-averageTree(tree_squirrel1, method="quadratic.path.difference
")

##
##   Function is attempting to find the phylogeny with
##   minimum distance to all trees in set under the
##   "quadratic path difference" criterion...

##   Best SS so far = 171537.104050971

##
##   Solution found after 1 set of nearest neighbor interchanges.

#Meng tree
tree_squirrel2<-root(tree_squirrel3,outgroup=c("Paramys_copei","Paramys_delic
atus",
                                               "Pseudotomus_horribilis","Pseu
dotomus_oweni",
                                               "Pseudotomus_petersoni","Pseud
otomus_hians"),
                                                resolve.root=TRUE)

############ Save trees

write.tree(tree_squirrel2, file = "Calibrated_tree_meng")
tree_squirrel2<-read.newick("Calibrated_tree_meng")
```

```r
#2. Permutation test

library(coin) #one_way test

## Loading required package: survival

library(rcompanion) #pairwisePermutationTest
library(ggpubr) #ggboxplot

## Loading required package: ggplot2

library(car) #Levene's test

## Loading required package: carData

#directory
setwd("~/Desktop/Squirrel_June_8_2020/Code")

#Import squirrel data
squirrel.data1<-read.csv("squirrels_PEQ_res.csv", header=T)
squirrel.data<-squirrel.data1[, c("PEQ_Meng", "Ecology")]
squirrel.data2<-squirrel.data1[, c("OB_percentage", "Ecology")]
squirrel.data3<-squirrel.data1[, c("PL_percentage", "Ecology")]
squirrel.data4<-squirrel.data1[, c("Neocortex_surface_percentage", "Ecology")
]

comp<- list(c("Arboreal", "Fossorial"),c("Arboreal", "Scansorial"),
            c("Arboreal", "Terrestrial"),c("Fossorial", "Glider"),c("Fossoria
l", "Scansorial"))

###############PEQ permutation test

##ggplot - boxplot - PEQ
ggboxplot(squirrel.data1,x="Ecology", y="PEQ_Meng", fill="Ecology", palette=c
("chartreuse3", "chocolate4", "steelblue1","firebrick2","yellow"))+
  geom_point() +
  geom_text(aes(label = abbreviation ), hjust = 0, nudge_x = 0.05)+
  labs(x='Locomotion', y='Phylogenetic encephalization quotient')
```

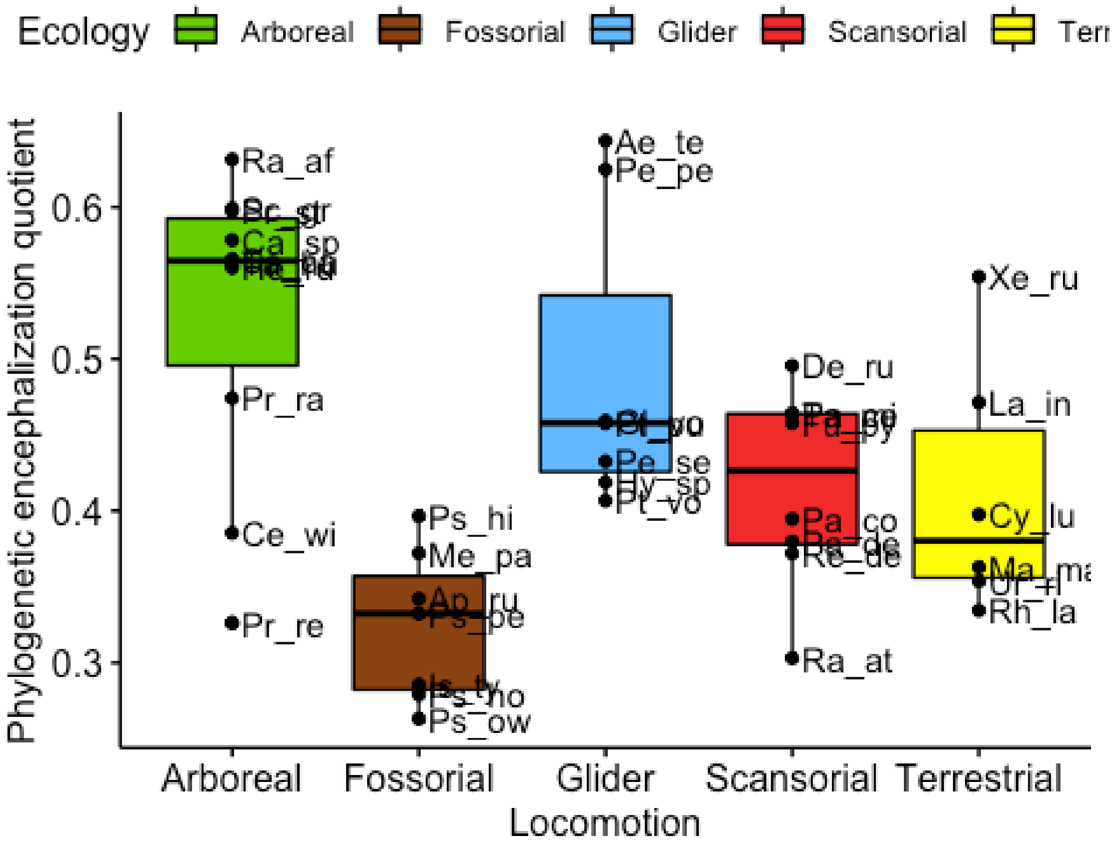


```r
#Fisher-Pitman permutation test
oneway_test(PEQ_Meng~Ecology,data=squirrel.data)

##
##  Asymptotic K-Sample Fisher-Pitman Permutation Test
##
## data:  PEQ_Meng by
##   Ecology (Arboreal, Fossorial, Glider, Scansorial, Terrestrial)
## chi-squared = 17.166, df = 4, p-value = 0.001794

squirrel.data$Ecology = factor(squirrel.data$Ecology, levels = c("Arboreal","
Fossorial","Glider",
                      "Scansorial","Terrestrial"))

PT_PEQ<-pairwisePermutationTest(PEQ_Meng~Ecology,data=squirrel.data,method="f
dr")
PT_PEQ

##                       Comparison   Stat  p.value p.adjust
## 1      Arboreal - Fossorial = 0  3.143 0.001672  0.01672
## 2         Arboreal - Glider = 0 0.7458   0.4558  0.50640
## 3     Arboreal - Scansorial = 0  2.319   0.0204  0.05100
## 4    Arboreal - Terrestrial = 0  2.065  0.03889  0.07778
## 5        Fossorial - Glider = 0 -2.721 0.006506  0.03253
```

```
## 6    Fossorial - Scansorial = 0 -2.414   0.0158  0.05100
## 7   Fossorial - Terrestrial = 0 -1.987  0.04687  0.07812
## 8       Glider - Scansorial = 0   1.65  0.09894  0.14130
## 9      Glider - Terrestrial = 0  1.459   0.1445  0.18060
## 10 Scansorial - Terrestrial = 0  0.106   0.9156  0.91560

#Test if data normally distributed (Normally distributed)
shapiro.test(squirrel.data$PEQ_Meng)

##
##  Shapiro-Wilk normality test
##
## data:  squirrel.data$PEQ_Meng
## W = 0.95275, p-value = 0.1097

# Bartlett test when data are normally distrubuted (YES there is homogeneity
of variances)
bartlett.test(PEQ_Meng ~ Ecology, data = squirrel.data)

##
##  Bartlett test of homogeneity of variances
##
## data:  PEQ_Meng by Ecology
## Bartlett's K-squared = 3.8936, df = 4, p-value = 0.4206

############Olfactory bulb permutation test

##ggplot - boxplot - OB
ggboxplot(squirrel.data1,x="Ecology", y="OB_percentage", fill="Ecology", pale
tte=c("chartreuse3", "chocolate4", "steelblue1","firebrick2","yellow"))+
  geom_point() +
  geom_text(aes(label = abbreviation ), hjust = 0, nudge_x = 0.05)+
  labs(x='Locomotion', y='Olfactory bulb volume percentage')
```

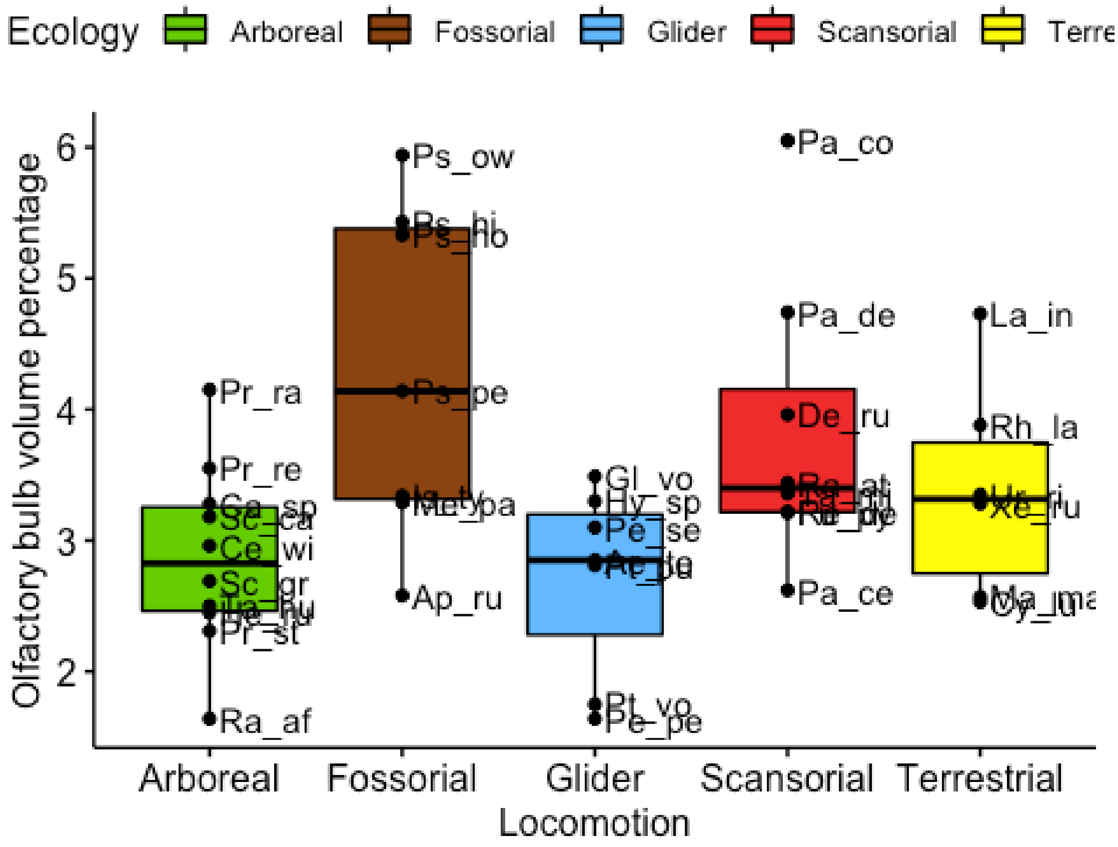


```
#Fisher-Pitman permutation test
oneway_test(OB_percentage~Ecology,data=squirrel.data2)

##
##  Asymptotic K-Sample Fisher-Pitman Permutation Test
##
## data:  OB_percentage by
##   Ecology (Arboreal, Fossorial, Glider, Scansorial, Terrestrial)
## chi-squared = 11.424, df = 4, p-value = 0.02219

squirrel.data2$Ecology = factor(squirrel.data2$Ecology, levels = c("Arboreal"
,"Fossorial","Glider",
                                                                 "Scansorial"
,"Terrestrial"))

PT_OB<-pairwisePermutationTest(OB_percentage~Ecology,data=squirrel.data2,meth
od="fdr")
PT_OB

##                      Comparison   Stat p.value p.adjust
## 1      Arboreal - Fossorial = 0 -2.416  0.0157   0.1117
## 2         Arboreal - Glider = 0 0.4785  0.6323   0.6323
## 3     Arboreal - Scansorial = 0 -2.014 0.04404   0.1117
## 4    Arboreal - Terrestrial = 0 -1.296  0.1949   0.2784
```

```
## 5        Fossorial - Glider = 0  2.284 0.02238   0.1117
## 6    Fossorial - Scansorial = 0 0.7732  0.4394   0.4882
## 7   Fossorial - Terrestrial = 0  1.403  0.1605   0.2675
## 8       Glider - Scansorial = 0 -2.007  0.0447   0.1117
## 9      Glider - Terrestrial = 0  -1.49  0.1363   0.2675
## 10 Scansorial - Terrestrial = 0 0.8222   0.411   0.4882

#Test if data normally distributed (not normally distributed)
shapiro.test(squirrel.data2$OB_percentage)

## 
##  Shapiro-Wilk normality test
## 
## data:  squirrel.data2$OB_percentage
## W = 0.92484, p-value = 0.01385

# Levene's test when data are not normally distrubuted (YES there is homogene
ity of variances)
leveneTest(OB_percentage ~ Ecology, data = squirrel.data2)

## Levene's Test for Homogeneity of Variance (center = median)
##       Df F value Pr(>F)
## group  4  0.9921 0.4256
##       33

#############Petrosal lobule permutation test

##ggplot - boxplot - Petrosal lobules
ggboxplot(squirrel.data1,x="Ecology", y="PL_percentage", fill="Ecology", pale
tte=c("chartreuse3", "chocolate4", "steelblue1","firebrick2","yellow"))+
  geom_point() +
  geom_text(aes(label = abbreviation ), hjust = 0, nudge_x = 0.05)+
  labs(x='Locomotion', y='Petrosal lobule volume percentage')

## Warning: Removed 1 rows containing non-finite values (stat_boxplot).

## Warning: Removed 1 rows containing missing values (geom_point).

## Warning: Removed 1 rows containing missing values (geom_text).
```

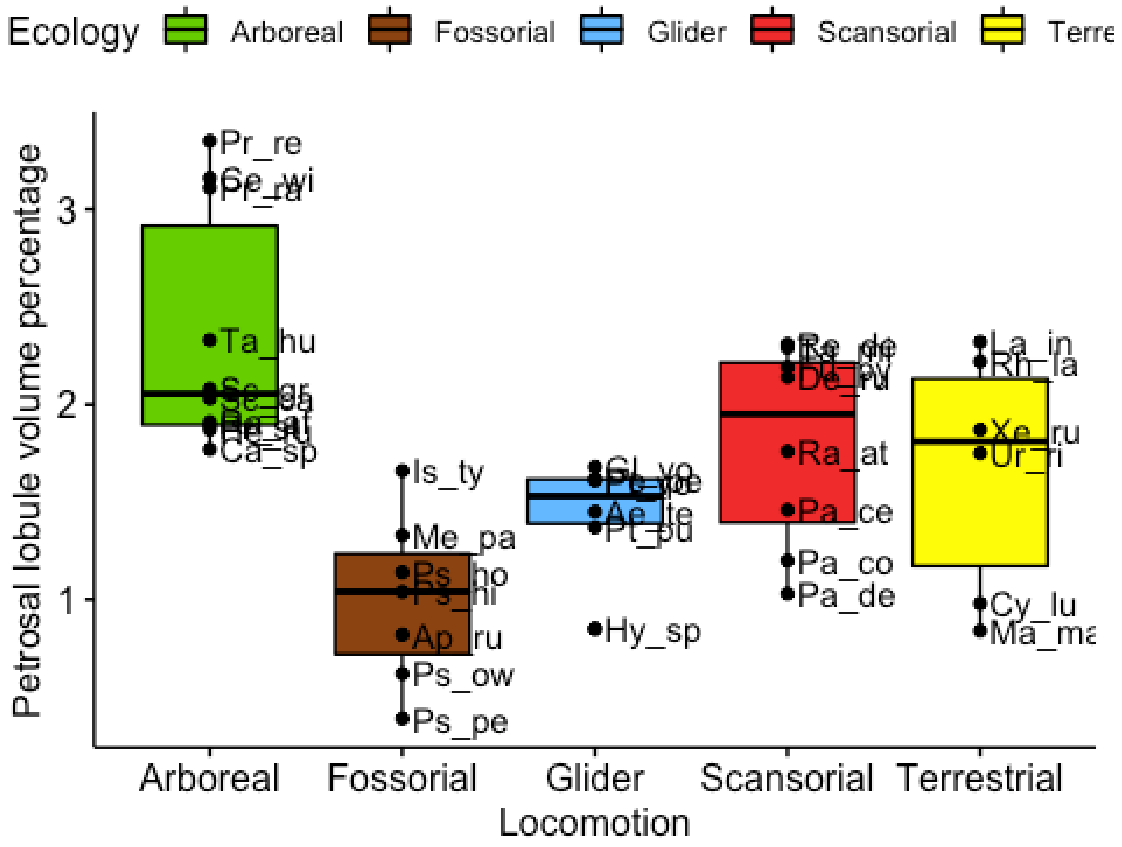


```
#Fisher-Pitman permutation test
oneway_test(PL_percentage~Ecology,data=squirrel.data3)

##
##  Asymptotic K-Sample Fisher-Pitman Permutation Test
##
## data:  PL_percentage by
##   Ecology (Arboreal, Fossorial, Glider, Scansorial, Terrestrial)
## chi-squared = 17.39, df = 4, p-value = 0.001623

squirrel.data3$Ecology = factor(squirrel.data3$Ecology, levels = c("Arboreal"
,"Fossorial","Glider",
                                                                 "Scansorial"
,"Terrestrial"))

PT_PL<-pairwisePermutationTest(PL_percentage~Ecology,data=squirrel.data3,meth
od="fdr")
PT_PL

##                       Comparison   Stat  p.value p.adjust
## 1      Arboreal - Fossorial = 0  3.163 0.001561  0.01561
## 2         Arboreal - Glider = 0  2.603 0.009242  0.04163
## 3     Arboreal - Scansorial = 0  1.873   0.0611  0.09999
## 4    Arboreal - Terrestrial = 0  1.935  0.05303  0.09999
```

```
## 5        Fossorial - Glider = 0 -1.812  0.06999  0.09999
## 6    Fossorial - Scansorial = 0 -2.498  0.01249  0.04163
## 7   Fossorial - Terrestrial = 0 -1.953  0.05082  0.09999
## 8       Glider - Scansorial = 0 -1.474   0.1406  0.17580
## 9      Glider - Terrestrial = 0 -0.836   0.4032  0.44800
## 10 Scansorial - Terrestrial = 0 0.4573   0.6474  0.64740

#Test if data normally distributed (Normally distributed)
shapiro.test(squirrel.data3$PL_percentage)

## 
##  Shapiro-Wilk normality test
## 
## data:  squirrel.data3$PL_percentage
## W = 0.97045, p-value = 0.4206

# Bartlett test when data are normally distrubuted (YES there is homogeneity
of variances)
bartlett.test(PL_percentage ~ Ecology, data = squirrel.data3)

## 
##  Bartlett test of homogeneity of variances
## 
## data:  PL_percentage by Ecology
## Bartlett's K-squared = 3.0935, df = 4, p-value = 0.5423

############ Neocortex permutation test

##ggplot - boxplot - Neocortex
ggboxplot(squirrel.data1,x="Ecology", y="Neocortex_surface_percentage", fill=
"Ecology", palette=c("chartreuse3", "chocolate4", "steelblue1","firebrick2","
yellow"))+
  geom_point() +
  geom_text(aes(label = abbreviation ), hjust = 0, nudge_x = 0.05)+
  labs(x='Locomotion', y='Neocortical surface area percentage')
```

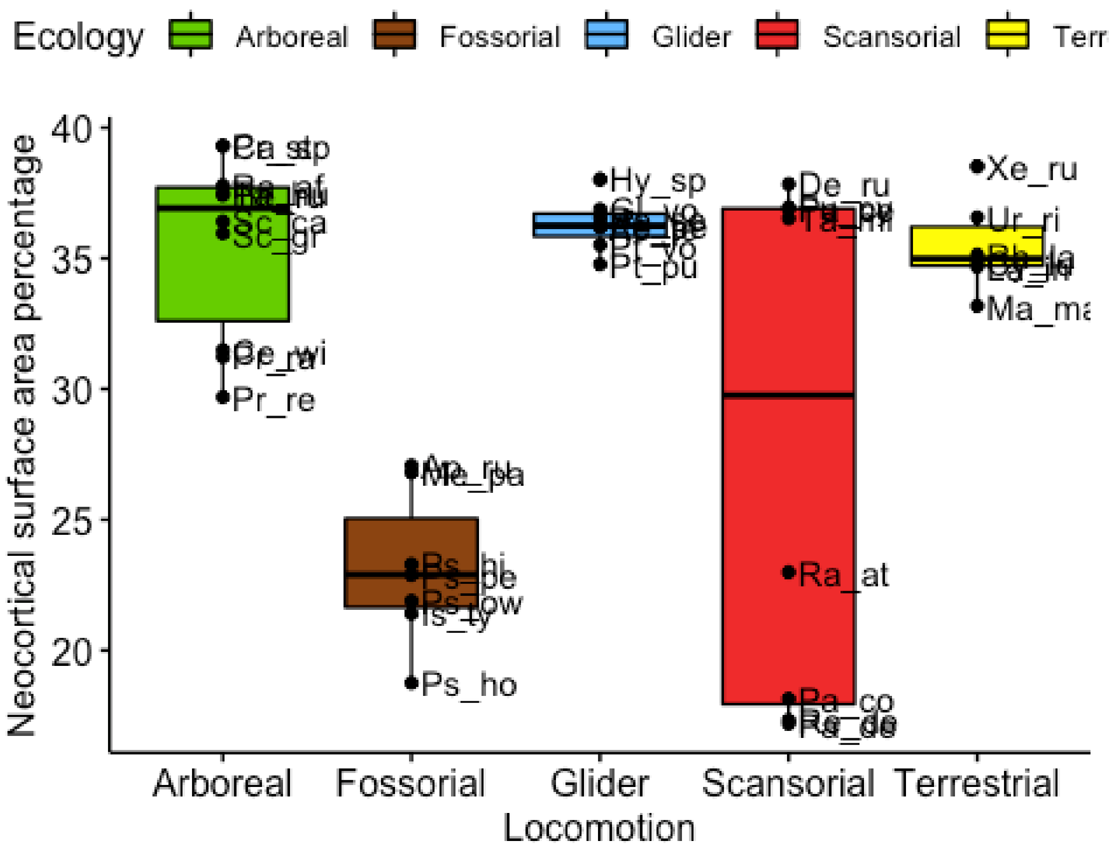


```
#Fisher-Pitman permutation test
oneway_test(Neocortex_surface_percentage~Ecology,data=squirrel.data4)

##
##  Asymptotic K-Sample Fisher-Pitman Permutation Test
##
## data:  Neocortex_surface_percentage by
##   Ecology (Arboreal, Fossorial, Glider, Scansorial, Terrestrial)
## chi-squared = 19.879, df = 4, p-value = 0.0005277

#independence_test(Neocortex_surface_percentage~Ecology,data=squirrel.data)

squirrel.data4$Ecology = factor(squirrel.data4$Ecology, levels = c("Arboreal"
,"Fossorial","Glider",
                                                                 "Scansorial"
,"Terrestrial"))

PT_Neo<-pairwisePermutationTest(Neocortex_surface_percentage~Ecology,data=squ
irrel.data4,method="fdr")
PT_Neo

##                      Comparison    Stat   p.value p.adjust
## 1      Arboreal - Fossorial = 0   3.569 0.0003586 0.002894
## 2         Arboreal - Glider = 0 -0.5191    0.6037 0.670800
```

```
## 3     Arboreal - Scansorial = 0    2.045   0.04088 0.101000
## 4    Arboreal - Terrestrial = 0 0.08116    0.9353 0.935300
## 5        Fossorial - Glider = 0  -3.441 0.0005787 0.002894
## 6    Fossorial - Scansorial = 0  -1.217    0.2234 0.319100
## 7   Fossorial - Terrestrial = 0  -3.242  0.001188 0.003960
## 8       Glider - Scansorial = 0   1.956   0.05048 0.101000
## 9      Glider - Terrestrial = 0   1.014    0.3108 0.388500
## 10 Scansorial - Terrestrial = 0  -1.678    0.0934 0.155700

#Test if data normally distributed (Not normally distributed)
shapiro.test(squirrel.data4$Neocortex_surface_percentage)

##
##  Shapiro-Wilk normality test
##
## data:  squirrel.data4$Neocortex_surface_percentage
## W = 0.81965, p-value = 2.696e-05

# Levene's test when data are not normally distrubuted (NO there is not homog
eneity of variances)
leveneTest(Neocortex_surface_percentage ~ Ecology, data = squirrel.data4)

## Levene's Test for Homogeneity of Variance (center = median)
##       Df F value    Pr(>F)
## group  4  20.898 1.158e-08 ***
##       33
## ---
## Signif. codes:  0 '***' 0.001 '**' 0.01 '*' 0.05 '.' 0.1 ' ' 1

### END
```

```
#3. PGLS regression for endocranial volume vs. body mass

#Can body mass and locomotion predict endocranial size?

library(phytools) #open tree

## Loading required package: ape

## Loading required package: maps

library(ggplot2) #plots
library(nlme) # GLS analysis
library(RRPP) #pairwise comparisons


#directory
setwd("~/Desktop/Squirrel_June_8_2020/Code")

#Import squirrel data
squirrel.data<-read.csv("squirrels_PEQ_res.csv", header=T)

#Import tree
tree_squirrel<-read.newick("Calibrated_tree_meng")

#Transform data to log10
squirrel.data$Brain_volume_cm3<-log10(squirrel.data$Brain_volume_cm3)
names(squirrel.data)[names(squirrel.data) == "Brain_volume_cm3"] <- "Brain"

squirrel.data$Body_mass_g<-log10(squirrel.data$Body_mass_g)
names(squirrel.data)[names(squirrel.data) == "Body_mass_g"] <- "Body"

#Select other variables
Locomotion<-squirrel.data$Locomotion
abbreviation<-squirrel.data$abbreviation

##########################  Analyses -- OLS ###############################

# Look at the correlation among data point by Family
ggplot(squirrel.data, aes(Body, Brain, color = Family)) +
  theme_light() + theme(legend.position = "top") +
  geom_point(data = dplyr::filter(squirrel.data, Family == "Sciuridae"), shap
e = 16, size = 3,
             aes(color = "#4DBBD5FF")) +
  geom_point(data = dplyr::filter(squirrel.data, Family == "Aplodontidae"), s
hape = 16, size = 3,
             aes(color = "#E64B35FF")) +
  geom_point(data = dplyr::filter(squirrel.data, Family == "Ischyromyidae"),
shape = 16, size = 3,
             aes(color = "#3C5488FF")) +
  scale_color_manual(name = "", values = c("#3C5488FF","#4DBBD5FF","#E64B35FF
"),labels = c("Ischyromyidae","Sciuridae", "Aplodontidae")) +
```

```
  labs(x = "log(Body mass)", y = "log(Endocranial volume)") +
  geom_text(data = dplyr::filter(squirrel.data, Family == "Sciuridae"), color
= "#4DBBD5FF",
            aes(label = abbreviation), hjust = -0.3, vjust = 1.1) +
  geom_text(data = dplyr::filter(squirrel.data, Family == "Ischyromyidae"), c
olor = "#3C5488FF",
            aes(label = abbreviation), hjust = -0.3, vjust = 1.1) +
  geom_text(data = dplyr::filter(squirrel.data, Family == "Aplodontidae"), co
lor = "#E64B35FF",
            aes(label = abbreviation), hjust = -0.3, vjust = 1.1)
```

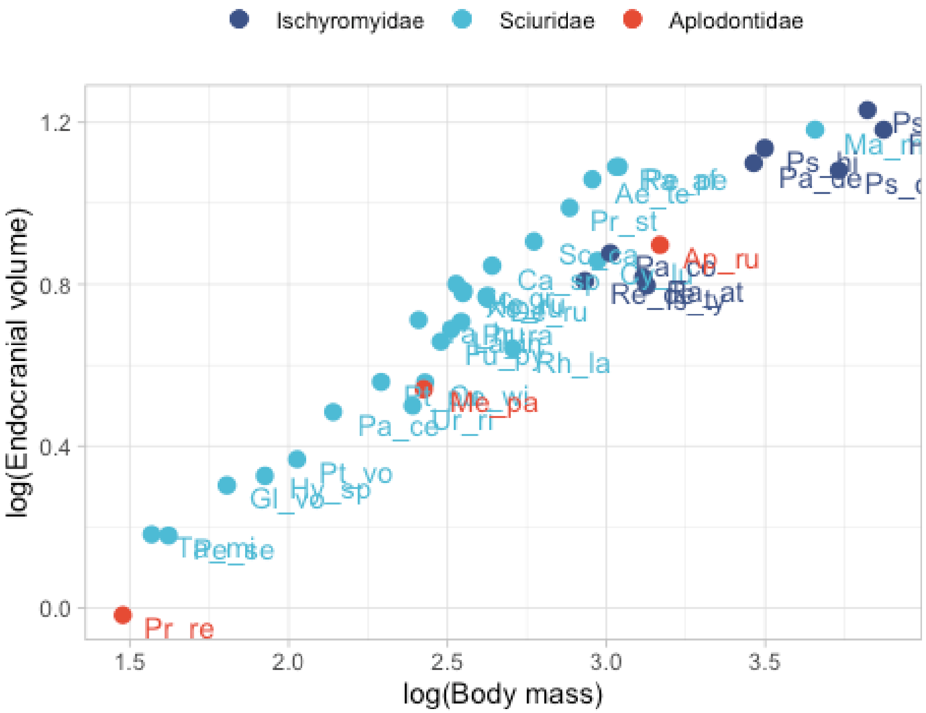


```
#Model using OLS (no phylogeny)
m.ols<-gls(Brain ~ Body + Locomotion, data=squirrel.data, method="ML")
m.ols.body<-gls(Brain ~ Body + Locomotion*Body, data=squirrel.data, method="M
L")
m.ols.loco<-gls(Brain ~ Body*Locomotion, data=squirrel.data, method="ML") #sa
me as above

#Chosing the model to use
anova(m.ols, m.ols.body, m.ols.loco) # m.ols.body (and m.ols.loco) with lowes
t AIC

##            Model df       AIC       BIC   logLik   Test  L.Ratio p-value
## m.ols          1  4 -66.04939 -59.49904 37.02469
## m.ols.body     2  5 -69.94684 -61.75891 39.97342 1 vs 2 5.897457  0.0152
## m.ols.loco     3  5 -69.94684 -61.75891 39.97342
```

```r
summary(m.ols.body) # Body mass alone + Interaction between Body mass and Loc
omotion have a siginificant effect on brain size

## Generalized least squares fit by maximum likelihood
##   Model: Brain ~ Body + Locomotion * Body
##   Data: squirrel.data
##         AIC       BIC   logLik
##   -69.94684 -61.75891 39.97342
##
## Coefficients:
##                      Value  Std.Error   t-value p-value
## (Intercept)     -0.9281245 0.18655820 -4.974986  0.0000
## Body             0.6557285 0.07061631  9.285794  0.0000
## Locomotion       0.1105096 0.06006813  1.839738  0.0746
## Body:Locomotion -0.0518547 0.02170400 -2.389176  0.0226
##
##  Correlation:
##                 (Intr) Body   Locmtn
## Body            -0.985
## Locomotion      -0.927  0.900
## Body:Locomotion  0.933 -0.934 -0.984
##
## Standardized residuals:
##           Min            Q1           Med            Q3           Max
## -2.3233180077 -0.7903876637 -0.0004586674  0.7542860629  2.0952026247
##
## Residual standard error: 0.08451125
## Degrees of freedom: 38 total; 34 residual

#Residuals vs fitted plot
plot(fitted(m.ols.body), residuals(m.ols.body))
abline(0,0) #pattern visible so not good to use OLS
```

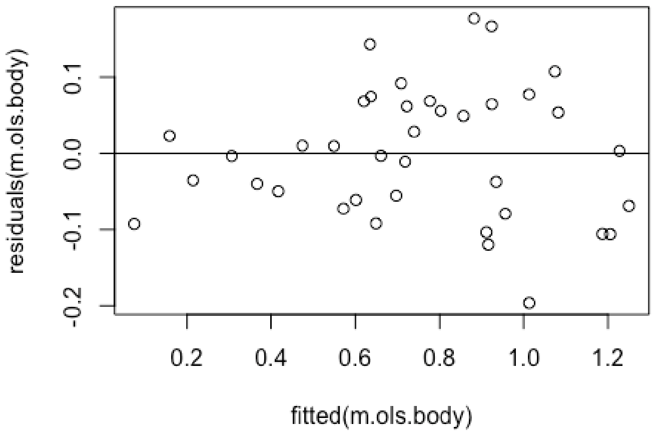

```r
# Plot residuals ordered "by phylogeny" (Goldbogen et al., 2019; Supp. p.58)
is_tip <- tree_squirrel$edge[,2]<=length(tree_squirrel$tip.label)
ordered_tips <- tree_squirrel$edge[is_tip,2] # extract the order of tree tips
oj <- residuals(m.ols.body)
tl <- tree_squirrel$tip.label[ordered_tips] # check order in tree to put them
in same order in csv. file

#Plot residuals against phylogeny
Spe<-squirrel.data$Sp
ggplot(squirrel.data, aes(x=Spe, y = oj, color = Family))+
  theme_light() + theme(legend.position = "top") +
  labs(x = "Species index", y = "OLS residuals") +
  geom_point() +
  geom_abline(intercept = 0, slope = 0) ## pattern, i.e., red ones are very l
ow so need PGLS
```

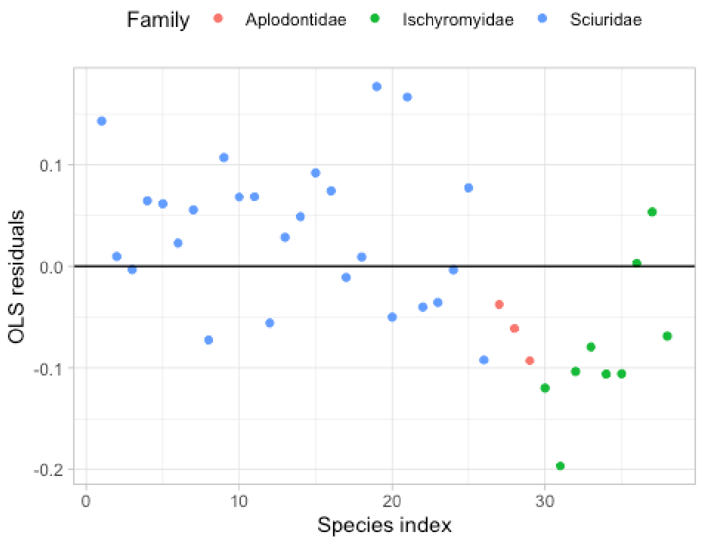


```r
##########################  Analyses -- PGLS ###############################

### Select PGLS model
# Model with locomotion only
Lambda<-gls(Brain ~ Body + Locomotion, data=squirrel.data,
            correlation=corPagel(value=1,phy=tree_squirrel), method="ML")

Brownian<-gls(Brain ~ Body + Locomotion, data=squirrel.data,
            correlation=corBrownian(1,phy=tree_squirrel), method="ML")

OU<-gls(Brain ~ Body + Locomotion, data=squirrel.data,
            correlation=corMartins(1,phy=tree_squirrel), method="ML")

Blomberg<-gls(Brain ~ Body + Locomotion, data=squirrel.data,
            correlation=corBlomberg(1.5,phy=tree_squirrel,fixed = TRUE), meth
od="ML") #if 1, this is a Brownian model
```

```
# Model with interaction betwenn Locomotion and Body mass
Lambda1<-gls(Brain ~ Body + Locomotion*Body, data=squirrel.data,
             correlation=corPagel(value=1,phy=tree_squirrel), method="ML")

Brownian1<-gls(Brain ~ Body + Locomotion*Body, data=squirrel.data,
             correlation=corBrownian(1,phy=tree_squirrel), method="ML")

OU1<-gls(Brain ~ Body + Locomotion*Body, data=squirrel.data,
             correlation=corMartins(1,phy=tree_squirrel, fixed = TRUE), metho
d="ML")

Blomberg1<-gls(Brain ~ Body + Locomotion*Body, data=squirrel.data,
             correlation=corBlomberg(1.5,phy=tree_squirrel,fixed = TRUE), met
hod="ML") #if 1, this is a Brownian model

#Chosing the best model using AIC
anova(Lambda,Brownian,OU, Blomberg) # Lambda is the best

##            Model df       AIC       BIC   logLik   Test   L.Ratio p-value
## Lambda         1  5 -75.31051 -67.12258 42.65526
## Brownian       2  4 -64.27501 -57.72467 36.13750 1 vs 2 13.035501  0.0003
## OU             3  5 -71.27298 -63.08505 40.63649 2 vs 3  8.997968  0.0027
## Blomberg       4  4 -61.88394 -55.33360 34.94197 3 vs 4 11.389035  0.0007

anova(Lambda1,Brownian1,OU1, Blomberg1) # Lambda1 is the best

##             Model df       AIC       BIC   logLik   Test  L.Ratio p-value
## Lambda1         1  6 -79.08012 -69.25461 45.54006
## Brownian1       2  5 -65.33614 -57.14821 37.66807 1 vs 2 15.74399   1e-04
## OU1             3  5 -72.03287 -63.84494 41.01644
## Blomberg1       4  5 -62.95271 -54.76478 36.47635

anova(Lambda,Lambda1) # Lambda1 is the best model overall

##           Model df       AIC       BIC   logLik   Test  L.Ratio p-value
## Lambda        1  5 -75.31051 -67.12258 42.65526
## Lambda1       2  6 -79.08012 -69.25461 45.54006 1 vs 2 5.769614  0.0163

summary(Lambda1) #Final model

## Generalized least squares fit by maximum likelihood
##   Model: Brain ~ Body + Locomotion * Body
##   Data: squirrel.data
##         AIC       BIC   logLik
##   -79.08012 -69.25461 45.54006
##
## Correlation Structure: corPagel
##  Formula: ~1
##  Parameter estimate(s):
##    lambda
## 0.7224339
##
```

```
## Coefficients:
##                      Value  Std.Error   t-value p-value
## (Intercept)     -1.0746318 0.16037510 -6.700740  0.0000
## Body             0.6913857 0.05683257 12.165307  0.0000
## Locomotion       0.0772706 0.04862054  1.589258  0.1213
## Body:Locomotion -0.0424188 0.01788268 -2.372059  0.0235
##
##  Correlation:
##                 (Intr) Body   Locmtn
## Body            -0.953
## Locomotion      -0.841  0.870
## Body:Locomotion  0.854 -0.908 -0.984
##
## Standardized residuals:
##        Min         Q1        Med         Q3        Max
## -1.4906549  0.1173624  0.8827299  1.2176829  2.3801169
##
## Residual standard error: 0.1028405
## Degrees of freedom: 38 total; 34 residual

#Lambda = 0.72 (brain size shows some phylogenetic signal)
#Coefficients: p-value interpretation:
#Locomotion alone does not predict brain size (p-value = 0.12)
#Body mass alone and the interaction between Body mass and locomotion
#can predict brain size (p-value = 0 and 0.02)

################# post ad-hoc test on locomotion ################

#tutorial RRPP: https://cran.r-project.org/web/packages/RRPP/vignettes/Using.
RRPP.html

#Import squirrel data - to run pairwise test
squirrel.data<-read.csv("squirrels_PEQ_res.csv", header=T)
Brain<-log10(squirrel.data$Brain_volume_cm3)
Body<-log10(squirrel.data$Body_mass_g)
Ecology<-squirrel.data$Ecology

#Pairwise comparisons - non-phylogenetic! (Weisbecker et al., 2019; line 552
in Analyses)
interaction_frame <- rrpp.data.frame(brain=Brain,body=Body,loco=Ecology)
fit<-lm.rrpp(brain~body+loco*body,SS.type = c("I"),data=interaction_frame)

summary(fit, formula = FALSE)

##
## Linear Model fit with lm.rrpp
##
## Number of observations: 38
## Number of dependent variables: 1
## Data space dimensions: 1
```

```
## Sums of Squares and Cross-products: Type I
## Number of permutations: 1000
##
## Full Model Analysis of Variance
##
##     Df Residual Df       SS Residual SS      Rsq        F Z (from F)
## fit  9          28 3.406654   0.1023074 0.970844 103.5945   7.983139
##           Pr(>F)
## fit 0.0006666667
##
##
## Redundancy Analysis (PCA on fitted values and residuals)
##
##                 Trace Proportion Rank
## Fitted    0.09207173   0.970844    1
## Residuals 0.00276506   0.029156    1
## Total     0.09483679   1.000000    1
##
## Eigenvalues
##
##                   PC1
## Fitted    0.09207173
## Residuals 0.00276506
## Total     0.09483679
```

```
anova(fit) # When phylogeny is NOT taken into account, all predict brain size
```

```
##
## Analysis of Variance, using Residual Randomization
## Permutation procedure: Randomization of null model residuals
## Number of permutations: 1000
## Estimation method: Ordinary Least Squares
## Sums of Squares and Cross-products: Type I
## Effect sizes (Z) based on F distributions
##
##           Df     SS      MS     Rsq        F      Z Pr(>F)
## body       1 3.1259 3.12587 0.89083 855.5041 3.6262  0.001 **
## loco       4 0.1689 0.04222 0.04813  11.5550 3.1313  0.001 **
## body:loco  4 0.1119 0.02798 0.03189   7.6565 2.8044  0.001 **
## Residuals 28 0.1023 0.00365 0.02916
## Total     37 3.5090
## ---
## Signif. codes:  0 '***' 0.001 '**' 0.01 '*' 0.05 '.' 0.1 ' ' 1
##
## Call: lm.rrpp(f1 = brain ~ body + loco * body, SS.type = c("I"), data = in
teraction_frame)
```

```
Interactions <- pairwise(fit,covariate=interaction_frame$body,
                            groups=interaction_frame$loco)
```

```r
summary(Interactions, test.type="dist") # shows which locomotor modes are sig
nificantly different

##
## Pairwise comparisons
##
## Groups: Arboreal Fossorial Glider Scansorial Terrestrial
##
## RRPP: 1000 permutations
##
## Slopes (vectors of variate change per one unit of covariate change, by gro
up):
## Vectors hidden (use show.vectors = TRUE to view)
##
## Slope vector lengths
##    Arboreal   Fossorial      Glider  Scansorial Terrestrial
##   0.7183124   0.4686482   0.6646996   0.4504875   0.4712924
##
## Pairwise absolute difference (d) between vector lengths, plus statistics
##                                   d UCL (95%)          Z Pr > d
## Arboreal:Fossorial     0.249664161 0.1605955  3.6571261  0.001
## Arboreal:Glider        0.053612811 0.1567525 -0.2762728  0.523
## Arboreal:Scansorial    0.267824847 0.1507500  4.5125421  0.001
## Arboreal:Terrestrial   0.247019959 0.1893927  2.9057389  0.009
## Fossorial:Glider       0.196051351 0.1523771  2.8304821  0.011
## Fossorial:Scansorial   0.018160685 0.1509679 -0.9359826  0.806
## Fossorial:Terrestrial  0.002644202 0.1806930 -1.3131130  0.980
## Glider:Scansorial      0.214212036 0.1405612  3.6072137  0.003
## Glider:Terrestrial     0.193407149 0.1708897  2.3111329  0.020
## Scansorial:Terrestrial 0.020804887 0.1666478 -0.9538154  0.803

#Ex: Arboreal rodents have significantly different brain size compared to Fos
sorial
#Scansorial, and Terrestrial when accounting for body mass but not phylogeny

# Based on the fit model, how does brain size predicted to vary for each loco
motor category? (what I understand this does)
sizeDF <- data.frame(loco = c("Arboreal", "Scansorial","Glider","Terrestrial"
,"Fossorial"))
rownames(sizeDF) <- c("Arboreal", "Scansorial","Glider","Terrestrial","Fossor
ial")
sizePreds <- predict(fit,sizeDF)

##
## Warning: Not all variables in model accounted for in newdata.
## Missing variables will be averaged from observed data for prediction.

plot(sizePreds) # The brain size of Arboreal and Glider are more similar than
with the other locomotor categories
```

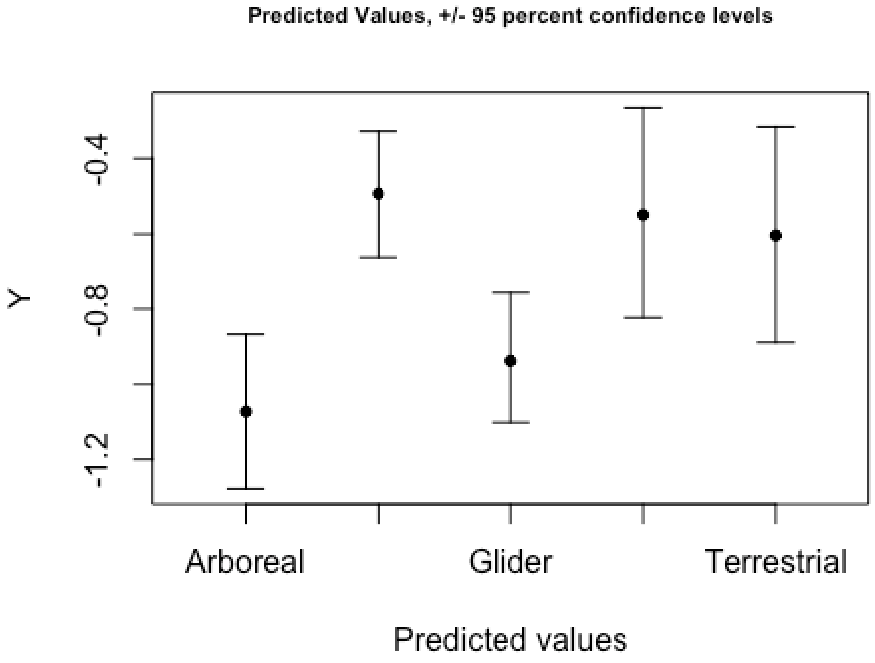


```
################ Make graph with PGLS regression lines for each locomotion ##
#########

#Import squirrel data
squirrel.data<-read.csv("squirrels_PEQ_res.csv", header=T)
squirrel.data$Brain_volume_cm3<-log10(squirrel.data$Brain_volume_cm3)
names(squirrel.data)[names(squirrel.data) == "Brain_volume_cm3"] <- "Brain"
squirrel.data$Body_mass_g<-log10(squirrel.data$Body_mass_g)
names(squirrel.data)[names(squirrel.data) == "Body_mass_g"] <- "Body"

#subset dataset per locomotion to use in model
Arb<- squirrel.data[ which(squirrel.data$Ecology=='Arboreal'), ]
Fos<- squirrel.data[ which(squirrel.data$Ecology=='Fossorial'), ]
Gli<- squirrel.data[ which(squirrel.data$Ecology=='Glider'), ]
Sca<- squirrel.data[ which(squirrel.data$Ecology=='Scansorial'), ]
Ter<- squirrel.data[ which(squirrel.data$Ecology=='Terrestrial'), ]

#Find taxa with specific locomotion
Arb_sp<-subset(squirrel.data, Ecology=="Arboreal", select=Species)
Fos_sp<-subset(squirrel.data, Ecology=="Fossorial", select=Species)
Gli_sp<-subset(squirrel.data, Ecology=="Glider", select=Species)
Sca_sp<-subset(squirrel.data, Ecology=="Scansorial", select=Species)
Ter_sp<-subset(squirrel.data, Ecology=="Terrestrial", select=Species)

#Select taxa per locomotion for the tree
Arb_species<-c("Protoxerus_stangeri","Heliosciurus_rufobrachium","Callosciuru
s_sp",
```

```
               "Sciurus_carolinensis","Sciurus_granatensis","Tamiasciurus_hud
sonicus",
               "Protosciurus_rachelae","Ratufa_affinis","Cedromus_wilsoni",
               "Prosciurus_relictus")

Fos_species<-c("Aplodontia_rufa","Mesogaulus_paniensis","Ischyromys_typus",
               "Pseudotomus_oweni","Pseudotomus_petersoni","Pseudotomus_hians
",
               "Pseudotomus_horribilis")

Gli_species<-c("Pteromyscus_pulverulentus","Aeromys_tephromelas","Pteromys_vo
lans",
               "Petaurista_petaurista","Hylopetes_spadiceus","Petinomys_setos
us",
               "Glaucomys_volans")

Sca_species<-c("Paraxerus_cepapi","Funisciurus_pyrropus","Tamias_minimus",
               "Dremomys_rufigenis","Rapamys_atramontis","Reithroparamys_deli
catissimus",
               "Paramys_copei","Paramys_delicatus")

Ter_species<-c("Xerus_rutilus","Cynomys_ludovicianus","Urocitellus_richardson
ii",
               "Marmota_marmota","Lariscus_insignis","Rhinosciurus_laticaudat
us")

#subset tree per locomotion
Arb_Tree<-drop.tip(tree_squirrel,tree_squirrel$tip.label[-match(Arb_species,
tree_squirrel$tip.label)])
Fos_Tree<-drop.tip(tree_squirrel,tree_squirrel$tip.label[-match(Fos_species,
tree_squirrel$tip.label)])
Gli_Tree<-drop.tip(tree_squirrel,tree_squirrel$tip.label[-match(Gli_species,
tree_squirrel$tip.label)])
Sca_Tree<-drop.tip(tree_squirrel,tree_squirrel$tip.label[-match(Sca_species,
tree_squirrel$tip.label)])
Ter_Tree<-drop.tip(tree_squirrel,tree_squirrel$tip.label[-match(Ter_species,
tree_squirrel$tip.label)])

#Create model PGLS regression line for each locomotion -- same warning
Arbline_Br_B <-gls(Brain ~ Body, correlation=corPagel (1,phy=Arb_Tree), data=
Arb)

Fosline_Br_B <-gls(Brain ~ Body, correlation=corPagel (1,phy=Fos_Tree), data=
Fos)

Gliline_Br_B <-gls(Brain ~ Body, correlation=corPagel (1,phy=Gli_Tree), data=
Gli)

Scaline_Br_B <-gls(Brain ~ Body, correlation=corPagel (1,phy=Sca_Tree), data=
Sca)
```

```
Terline_Br_B <-gls(Brain ~ Body, correlation=corPagel (1,phy=Ter_Tree), data=
Ter)

#Prepare PGLS for each locomotor mode
pgls.fit.Arb <- predict(Arbline_Br_B) #predict values for brain size
predframe.Arb <- with(Arb, data.frame(Species, Ecology, Body, Brain = pgls.fi
t.Arb))

pgls.fit.Fos <- predict(Fosline_Br_B) #predict values for brain size
predframe.Fos <- with(Fos, data.frame(Species, Ecology, Body, Brain = pgls.fi
t.Fos))

pgls.fit.Gli <- predict(Gliline_Br_B) #predict values for brain size
predframe.Gli <- with(Gli, data.frame(Species, Ecology, Body, Brain = pgls.fi
t.Gli))

pgls.fit.Sca <- predict(Scaline_Br_B) #predict values for brain size
predframe.Sca <- with(Sca, data.frame(Species, Ecology, Body, Brain = pgls.fi
t.Sca))

pgls.fit.Ter <- predict(Terline_Br_B) #predict values for brain size
predframe.Ter <- with(Ter, data.frame(Species, Ecology, Body, Brain = pgls.fi
t.Ter))

#Make graph with PGLS corrected regressions
ggplot(squirrel.data, aes(Body, Brain, color = Ecology)) +
  geom_point(data = dplyr::filter(squirrel.data, Ecology == "Arboreal"),
             size = 2, aes(color = "#0EAF28")) +
  geom_point(data = dplyr::filter(squirrel.data, Ecology == "Fossorial"),
             size = 2, aes(color = "#975822")) +
  geom_point(data = dplyr::filter(squirrel.data, Ecology == "Glider"),
             size = 2, aes(color = "#73DAF3")) +
  geom_point(data = dplyr::filter(squirrel.data, Ecology == "Scansorial"),
             size = 2, aes(color = "#F31616")) +
  geom_point(data = dplyr::filter(squirrel.data, Ecology == "Terrestrial"),
             size = 2, aes(color = "#F3B116")) +
  geom_line(data = dplyr::filter(predframe.Arb, Ecology == "Arboreal"), color
= "#0EAF28",
            linetype = 1.5) +
  geom_line(data = dplyr::filter(predframe.Fos, Ecology == "Fossorial"), colo
r = "#975822",
            linetype = 1.5) +
  geom_line(data = dplyr::filter(predframe.Gli, Ecology == "Glider"), color =
"#73DAF3",
            linetype = 1.5) +
  geom_line(data = dplyr::filter(predframe.Sca, Ecology == "Scansorial"), col
or = "#F31616",
            linetype = 1.5) +
  geom_line(data = dplyr::filter(predframe.Ter, Ecology == "Terrestrial"), co
lor = "#F3B116",
```

```
            linetype = 1.5) +
  theme_minimal() +
  #theme(legend.position = "top") +
  scale_color_manual(name = "", values = c("#0EAF28","#73DAF3","#975822", "#F
31616", "#F3B116"),labels = c("Arboreal",
            "Glider","Fossorial", "Scansorial", "Terrestrial")) +
  labs(x = "log(Body mass)", y = "log(Endocranial volume)") +
  theme(axis.text = element_text(size = 12), axis.title = element_text(size =
12),
            face = "bold")) +
  geom_text(data = dplyr::filter(squirrel.data, Ecology == "Arboreal"), color
= "#0EAF28",
          aes(label = abbreviation), hjust = -0.3, vjust = 1.1)+
  geom_text(data = dplyr::filter(squirrel.data, Ecology == "Fossorial"), colo
r = "#975822",
            aes(label = abbreviation), hjust = -0.3, vjust = 1.1) +
  geom_text(data = dplyr::filter(squirrel.data, Ecology == "Glider"), color =
"#73DAF3",
            aes(label = abbreviation), hjust = -0.3, vjust = 1.1) +
  geom_text(data = dplyr::filter(squirrel.data, Ecology == "Scansorial"), col
or = "#F31616",
            aes(label = abbreviation), hjust = -0.3, vjust = 1.1) +
  geom_text(data = dplyr::filter(squirrel.data, Ecology == "Terrestrial"), co
lor = "#F3B116",
            aes(label = abbreviation), hjust = -0.3, vjust = 1.1)
```

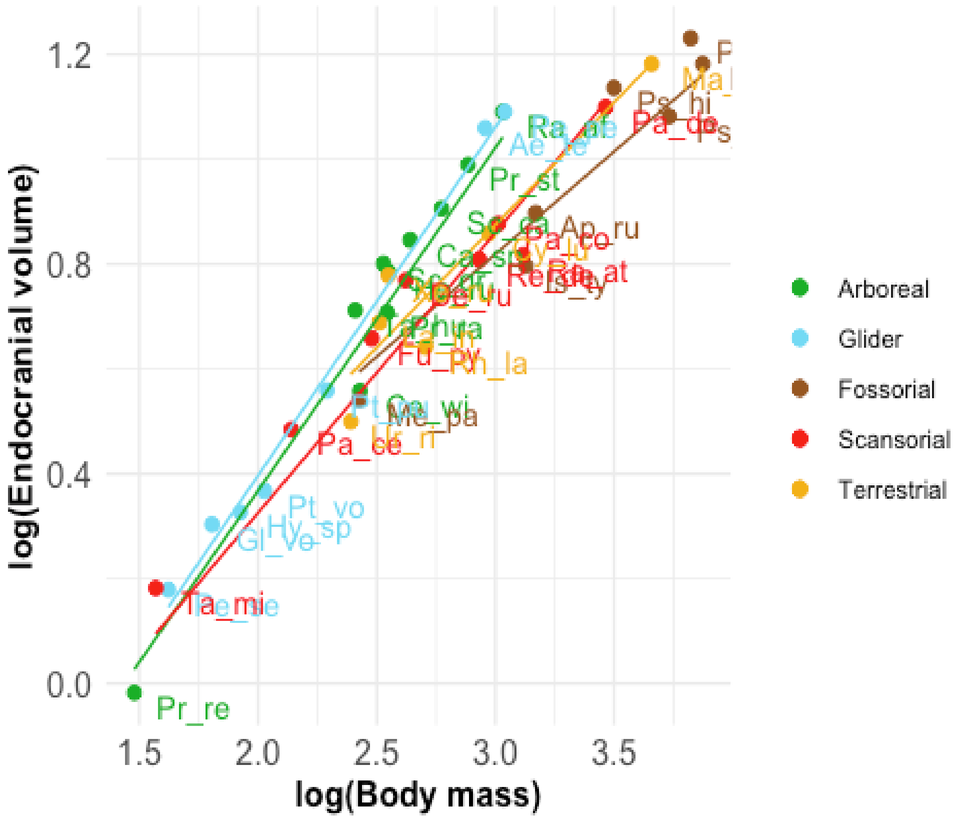

```
#4. PGLS regression for olfactory bulb volume vs. body mass

#Can body mass and locomotion predict olfactory bulb size?

library(phytools) #open tree

## Loading required package: ape

## Loading required package: maps

library(ggplot2) #plots
library(nlme) # GLS analysis
library(RRPP) #pairwise comparisons

#directory
setwd("~/Desktop/Squirrel_June_8_2020/Code")

#Import squirrel data
squirrel.data<-read.csv("squirrels_PEQ_res.csv", header=T)

#Import tree
tree_squirrel<-read.newick("Calibrated_tree_meng")

#Transform data to log10
squirrel.data$Olfactory_bulb_volume_mm3<-log10(squirrel.data$Olfactory_bulb_v
olume_mm3)
names(squirrel.data)[names(squirrel.data) == "Olfactory_bulb_volume_mm3"] <-
"OB"

squirrel.data$Body_mass_mg<-log10(squirrel.data$Body_mass_mg)
names(squirrel.data)[names(squirrel.data) == "Body_mass_mg"] <- "Body.mg"

#Select other variables
Locomotion<-squirrel.data$Locomotion
abbreviation<-squirrel.data$abbreviation

##########################  Analyses -- OLS ###############################

# Look at the correlation among data point by Family
ggplot(squirrel.data, aes(Body.mg, OB, color = Family)) +
  theme_light() + theme(legend.position = "top") +
  geom_point(data = dplyr::filter(squirrel.data, Family == "Sciuridae"), shap
e = 16, size = 3,
             aes(color = "#4DBBD5FF")) +
  geom_point(data = dplyr::filter(squirrel.data, Family == "Aplodontidae"), s
hape = 16, size = 3,
             aes(color = "#E64B35FF")) +
  geom_point(data = dplyr::filter(squirrel.data, Family == "Ischyromyidae"),
shape = 16, size = 3,
             aes(color = "#3C5488FF")) +
  scale_color_manual(name = "", values = c("#3C5488FF","#4DBBD5FF","#E64B35FF
```

```
"),labels = c("Ischyromyidae","Sciuridae", "Aplodontidae")) +
  labs(x ="log(Body mass)", y = "log(Olfactory bulb volume)") +
  geom_text(data = dplyr::filter(squirrel.data, Family == "Sciuridae"), color
= "#4DBBD5FF",
            aes(label = abbreviation), hjust = -0.3, vjust = 1.1) +
  geom_text(data = dplyr::filter(squirrel.data, Family == "Ischyromyidae"), c
olor = "#3C5488FF",
            aes(label = abbreviation), hjust = -0.3, vjust = 1.1) +
  geom_text(data = dplyr::filter(squirrel.data, Family == "Aplodontidae"), co
lor = "#E64B35FF",
            aes(label = abbreviation), hjust = -0.3, vjust = 1.1)
```

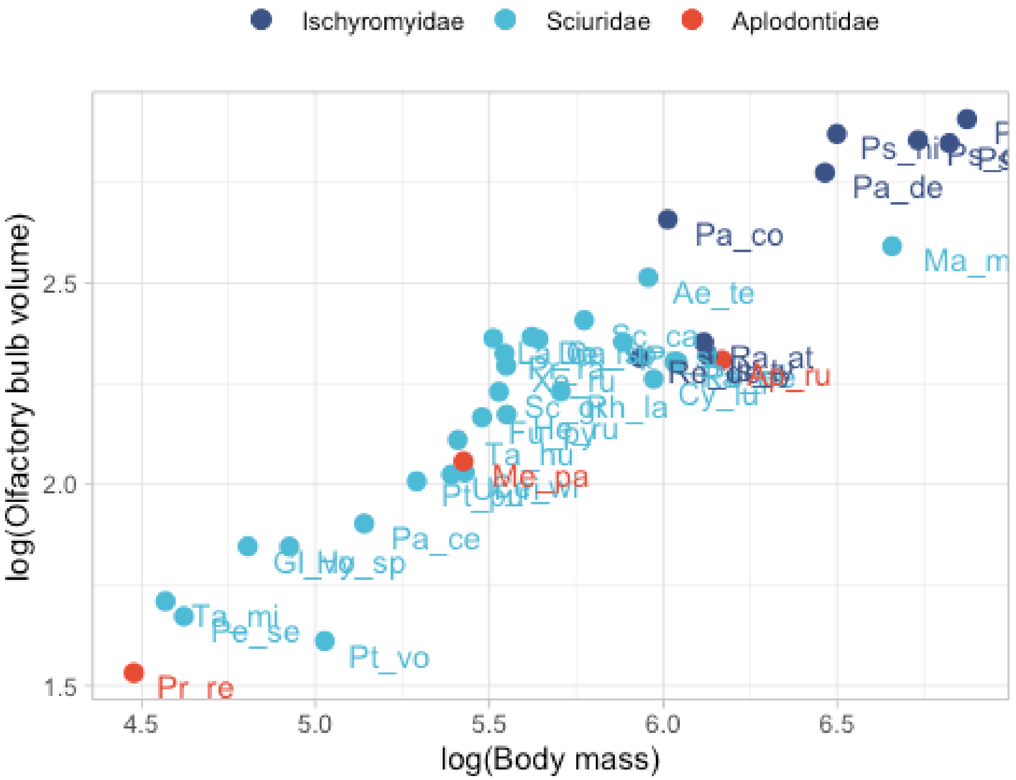


```
#Model using OLS (no phylogeny)
m.ols<-gls(OB ~ Body.mg + Locomotion, data=squirrel.data, method="ML")
m.ols.body<-gls(OB ~ Body.mg + Locomotion*Body.mg, data=squirrel.data, method
="ML")
m.ols.loco<-gls(OB ~ Body.mg*Locomotion, data=squirrel.data, method="ML") #sa
me as above

#Chosing the model to use
anova(m.ols, m.ols.body, m.ols.loco)

##              Model df       AIC       BIC   logLik   Test  L.Ratio p-value
## m.ols            1  4 -46.57807 -40.02773 27.28904
```

```
## m.ols.body     2  5 -45.61834 -37.43041 27.80917 1 vs 2 1.040269  0.3078
## m.ols.loco     3  5 -45.61834 -37.43041 27.80917
```

```
summary(m.ols)
```

```
## Generalized least squares fit by maximum likelihood
##   Model: OB ~ Body.mg + Locomotion
##   Data: squirrel.data
##         AIC       BIC   logLik
##   -46.57807 -40.02773 27.28904
##
## Coefficients:
##                  Value  Std.Error   t-value p-value
## (Intercept) -0.8215747 0.19092351 -4.303162  0.0001
## Body.mg      0.5474079 0.03471194 15.770018  0.0000
## Locomotion  -0.0152451 0.01468867 -1.037879  0.3064
##
##  Correlation:
##            (Intr) Bdy.mg
## Body.mg    -0.974
## Locomotion  0.096 -0.297
##
## Standardized residuals:
##          Min           Q1          Med           Q3          Max
## -2.320820800 -0.699495778  0.004454115  0.713435727  2.065083522
##
## Residual standard error: 0.118
## Degrees of freedom: 38 total; 35 residual
```

```
#Residuals vs fitted plot
plot(fitted(m.ols), residuals(m.ols))
abline(0,0) #pattern visible so not good to use OLS
```

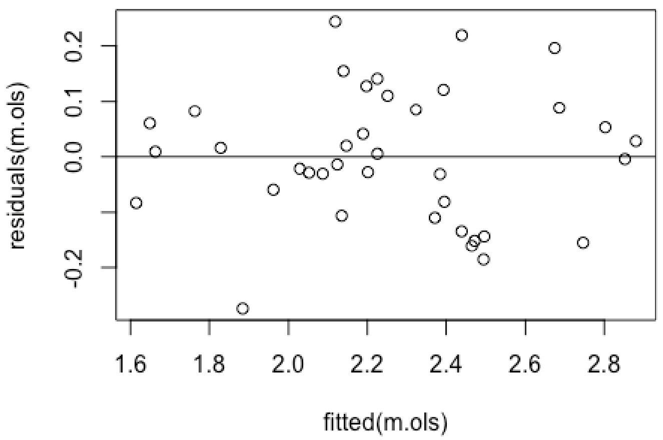

```
# Plot residuals ordered "by phylogeny" (Goldbogen et al., 2019; Supp. p.58)
is_tip <- tree_squirrel$edge[,2]<=length(tree_squirrel$tip.label)
ordered_tips <- tree_squirrel$edge[is_tip,2] # extract the order of tree tips
oj <- residuals(m.ols)
tl <- tree_squirrel$tip.label[ordered_tips] # check order in tree to put them
in same order in csv. file

#Plot residuals against phylogeny
Spe<-squirrel.data$Sp
ggplot(squirrel.data, aes(x=Spe, y = oj, color = Family))+
  theme_light() + theme(legend.position = "top") +
  labs(x = "Species index", y = "OLS residuals") +
  geom_point() +
  geom_abline(intercept = 0, slope = 0)
```

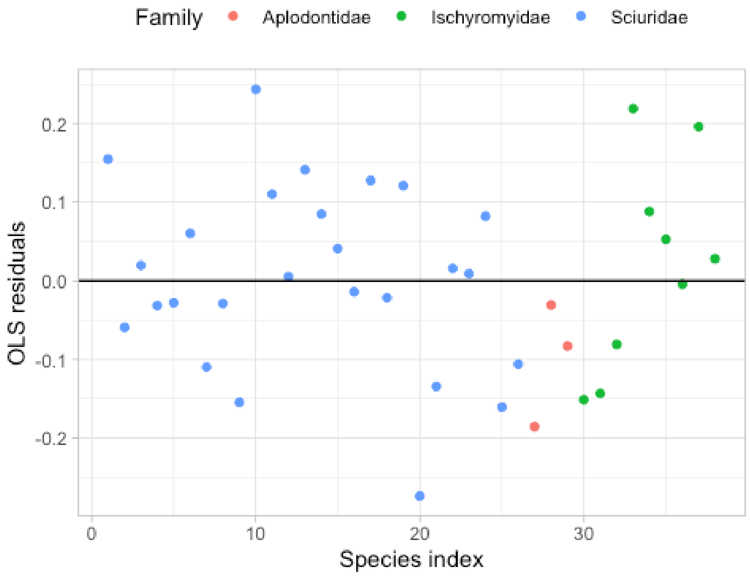


```
#########################  Analyses -- PGLS ##############################

### Select PGLS model
# Model with locomotion only
Lambda<-gls(OB ~ Body.mg + Locomotion, data=squirrel.data,
            correlation=corPagel(value=0.3,phy=tree_squirrel), method="ML") #
if value >0.3, need fixed value for lambda and at 1 = brownian

Brownian<-gls(OB ~ Body.mg + Locomotion, data=squirrel.data,
              correlation=corBrownian(1,phy=tree_squirrel), method="ML")

OU<-gls(OB ~ Body.mg + Locomotion, data=squirrel.data,
        correlation=corMartins(1,phy=tree_squirrel), method="ML")

Blomberg<-gls(OB ~ Body.mg + Locomotion, data=squirrel.data,
              correlation=corBlomberg(1.5,phy=tree_squirrel,fixed = TRUE), me
thod="ML") #if 1, this is a Brownian model
```

```
# Model with interaction betwenn Locomotion and Body mass
Lambda1<-gls(OB ~ Body.mg + Locomotion*Body.mg, data=squirrel.data,
             correlation=corPagel(value=0.3,phy=tree_squirrel), method="ML")
#if value >0.3, need fixed value for lambda and at 1 = brownian

Brownian1<-gls(OB ~ Body.mg + Locomotion*Body.mg, data=squirrel.data,
               correlation=corBrownian(1,phy=tree_squirrel), method="ML")

OU1<-gls(OB ~ Body.mg + Locomotion*Body.mg, data=squirrel.data,
         correlation=corMartins(1,phy=tree_squirrel), method="ML")

Blomberg1<-gls(OB ~ Body.mg + Locomotion*Body.mg, data=squirrel.data,
               correlation=corBlomberg(1.5,phy=tree_squirrel,fixed = TRUE), m
ethod="ML") #if 1, this is a Brownian model

#Chosing the best model using AIC
anova(Lambda,Brownian,OU, Blomberg) # OU is the best but not significantly di
fferent from Lambda

##            Model df       AIC       BIC   logLik   Test  L.Ratio p-value
## Lambda         1  5 -44.94728 -36.75935 27.47364
## Brownian       2  4 -23.25690 -16.70656 15.62845 1 vs 2 23.69038  <.0001
## OU             3  5 -47.93039 -39.74246 28.96520 2 vs 3 26.67349  <.0001
## Blomberg       4  4 -20.68741 -14.13707 14.34370 3 vs 4 29.24298  <.0001

anova(Lambda1,Brownian1,OU1, Blomberg1) # OU1 is the best but not significant
ly different from Lambda1

##             Model df       AIC       BIC   logLik   Test  L.Ratio p-value
## Lambda1         1  6 -45.28023 -35.45472 28.64012
## Brownian1       2  5 -25.11653 -16.92859 17.55826 1 vs 2 22.16371  <.0001
## OU1             3  6 -47.71008 -37.88456 29.85504 2 vs 3 24.59355  <.0001
## Blomberg1       4  5 -22.58109 -14.39316 16.29055 3 vs 4 27.12899  <.0001

anova(OU,OU1) # not significantly different but OU chosen because less comple
x

##      Model df       AIC       BIC   logLik   Test  L.Ratio p-value
## OU       1  5 -47.93039 -39.74246 28.96520
## OU1      2  6 -47.71008 -37.88456 29.85504 1 vs 2 1.779688  0.1822

summary(OU) #Final model

## Generalized least squares fit by maximum likelihood
##   Model: OB ~ Body.mg + Locomotion
##   Data: squirrel.data
##         AIC       BIC  logLik
##   -47.93039 -39.74246 28.9652
##
## Correlation Structure: corMartins
##  Formula: ~1
##  Parameter estimate(s):
```

```
##     alpha
## 0.4310211
##
## Coefficients:
##                  Value  Std.Error   t-value p-value
## (Intercept) -0.6246146 0.20249991 -3.084518  0.0040
## Body.mg      0.5105187 0.03621466 14.097016  0.0000
## Locomotion  -0.0144270 0.01467002 -0.983431  0.3321
##
##  Correlation:
##            (Intr) Bdy.mg
## Body.mg    -0.973
## Locomotion  0.151 -0.328
##
## Standardized residuals:
##         Min          Q1         Med          Q3         Max
## -2.35438345 -0.69503125 -0.05674445  0.82440425  2.01172349
##
## Residual standard error: 0.1222553
## Degrees of freedom: 38 total; 35 residual

#Coefficients: p-value interpretation:
#Body mass alone predicts but Locomotion cannot predict olfactory bulb size (
p-value > 0.05)

#To obtain lambda
summary(Lambda)

## Generalized least squares fit by maximum likelihood
##   Model: OB ~ Body.mg + Locomotion
##   Data: squirrel.data
##         AIC       BIC   logLik
##   -44.94728 -36.75935 27.47364
##
## Correlation Structure: corPagel
##  Formula: ~1
##  Parameter estimate(s):
##    lambda
## 0.2928719
##
## Coefficients:
##                  Value  Std.Error   t-value p-value
## (Intercept) -0.6057751 0.23134189 -2.618527  0.0130
## Body.mg      0.5147091 0.03912356 13.155988  0.0000
## Locomotion  -0.0160310 0.01452097 -1.103989  0.2771
##
##  Correlation:
##            (Intr) Bdy.mg
## Body.mg    -0.970
## Locomotion  0.117 -0.287
```

```
##
## Standardized residuals:
##        Min         Q1        Med         Q3        Max
## -2.5580613 -0.9596796 -0.3512270  0.4749398  1.6795793
##
## Residual standard error: 0.1262393
## Degrees of freedom: 38 total; 35 residual

#Lambda = 0.29 (olfactory bulb size shows little phylogenetic signal)

################# post ad-hoc test on locomotion ################

#tutorial RRPP: https://cran.r-project.org/web/packages/RRPP/vignettes/Using.
RRPP.html

#Import squirrel data - to run pairwise test
squirrel.data<-read.csv("squirrels_PEQ_res.csv", header=T)
OB<-log10(squirrel.data$Olfactory_bulb_volume_mm3)
Body<-log10(squirrel.data$Body_mass_g)
Ecology<-squirrel.data$Ecology

#Pairwise comparisons - non-phylogenetic! (Weisbecker et al., 2019; line 552
in Analyses)
interaction_frame <- rrpp.data.frame(ob=OB,body=Body,loco=Ecology)
fit<-lm.rrpp(ob~body+loco,SS.type = c("I"),data=interaction_frame)

summary(fit, formula = FALSE)

##
## Linear Model fit with lm.rrpp
##
## Number of observations: 38
## Number of dependent variables: 1
## Data space dimensions: 1
## Sums of Squares and Cross-products: Type I
## Number of permutations: 1000
##
## Full Model Analysis of Variance
##
##     Df Residual Df       SS Residual SS       Rsq        F Z (from F)  Pr(
>F)
## fit  5          32 3.998087    0.511958 0.8864849 49.98019   5.471913 0.00
075
##
##
## Redundancy Analysis (PCA on fitted values and residuals)
##
##                Trace Proportion Rank
## Fitted    0.1080564   0.886485    1
## Residuals 0.0138367   0.113515    1
```

```
## Total     0.1218931   1.000000    1
##
## Eigenvalues
##
##                 PC1
## Fitted    0.1080564
## Residuals 0.0138367
## Total     0.1218931
```

```
anova(fit) # When phylogeny is NOT taken into account, locomotion does not pr
edict olfactory bulb size
```

```
##
## Analysis of Variance, using Residual Randomization
## Permutation procedure: Randomization of null model residuals
## Number of permutations: 1000
## Estimation method: Ordinary Least Squares
## Sums of Squares and Cross-products: Type I
## Effect sizes (Z) based on F distributions
##
##           Df     SS     MS     Rsq        F        Z Pr(>F)
## body       1 3.9646 3.9646 0.87907 247.8108  2.97706  0.001 **
## loco       4 0.0334 0.0084 0.00741   0.5225 -0.52322  0.725
## Residuals 32 0.5120 0.0160 0.11352
## Total     37 4.5100
## ---
## Signif. codes:  0 '***' 0.001 '**' 0.01 '*' 0.05 '.' 0.1 ' ' 1
##
## Call: lm.rrpp(f1 = ob ~ body + loco, SS.type = c("I"), data = interaction_
frame)
```

```
#No pairwise performed because p-value <0.05 for locomotion

# Based on the fit model, how does brain size predicted to vary for each loco
motor category? (what I understand this does)
sizeDF <- data.frame(loco = c("Arboreal", "Scansorial","Glider","Terrestrial"
,"Fossorial"))
rownames(sizeDF) <- c("Arboreal", "Scansorial","Glider","Terrestrial","Fossor
ial")
sizePreds <- predict(fit,sizeDF)
```

```
##
## Warning: Not all variables in model accounted for in newdata.
## Missing variables will be averaged from observed data for prediction.
```

```
plot(sizePreds)
```

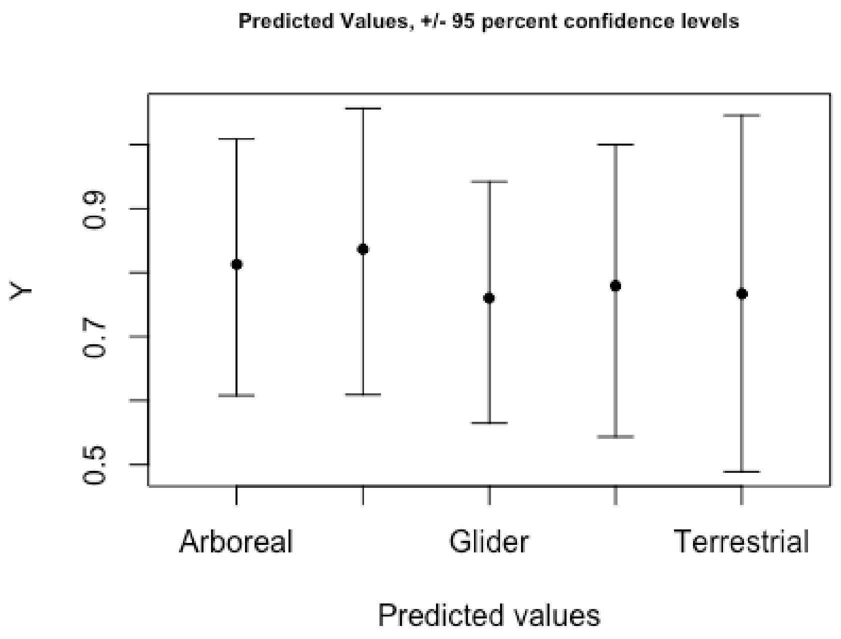


```
################ Make graph with PGLS regression lines for each locomotion ##
#########

#Import squirrel data
squirrel.data<-read.csv("squirrels_PEQ_res.csv", header=T)

#Select data
squirrel.data$Olfactory_bulb_volume_mm3<-log10(squirrel.data$Olfactory_bulb_v
olume_mm3)
names(squirrel.data)[names(squirrel.data) == "Olfactory_bulb_volume_mm3"] <-
"OB"
squirrel.data$Body_mass_mg<-log10(squirrel.data$Body_mass_mg)
names(squirrel.data)[names(squirrel.data) == "Body_mass_mg"] <- "Body.mg"

#subset dataset per locomotion to use in model
Arb<- squirrel.data[ which(squirrel.data$Ecology=='Arboreal'), ]
Fos<- squirrel.data[ which(squirrel.data$Ecology=='Fossorial'), ]
Gli<- squirrel.data[ which(squirrel.data$Ecology=='Glider'), ]
Sca<- squirrel.data[ which(squirrel.data$Ecology=='Scansorial'), ]
Ter<- squirrel.data[ which(squirrel.data$Ecology=='Terrestrial'), ]

#Find taxa with specific locomotion
Arb_sp<-subset(squirrel.data, Ecology=="Arboreal", select=Species)
Fos_sp<-subset(squirrel.data, Ecology=="Fossorial", select=Species)
Gli_sp<-subset(squirrel.data, Ecology=="Glider", select=Species)
Sca_sp<-subset(squirrel.data, Ecology=="Scansorial", select=Species)
Ter_sp<-subset(squirrel.data, Ecology=="Terrestrial", select=Species)
```

```r
#Select taxa per locomotion for the tree
Arb_species<-c("Protoxerus_stangeri","Heliosciurus_rufobrachium","Callosciuru
s_sp",
               "Sciurus_carolinensis","Sciurus_granatensis","Tamiasciurus_hud
sonicus",
               "Protosciurus_rachelae","Ratufa_affinis","Cedromus_wilsoni",
               "Prosciurus_relictus")

Fos_species<-c("Aplodontia_rufa","Mesogaulus_paniensis","Ischyromys_typus",
               "Pseudotomus_oweni","Pseudotomus_petersoni","Pseudotomus_hians
",
               "Pseudotomus_horribilis")

Gli_species<-c("Pteromyscus_pulverulentus","Aeromys_tephromelas","Pteromys_vo
lans",
               "Petaurista_petaurista","Hylopetes_spadiceus","Petinomys_setos
us",
               "Glaucomys_volans")

Sca_species<-c("Paraxerus_cepapi","Funisciurus_pyrropus","Tamias_minimus",
               "Dremomys_rufigenis","Rapamys_atramontis","Reithroparamys_deli
catissimus",
               "Paramys_copei","Paramys_delicatus")

Ter_species<-c("Xerus_rutilus","Cynomys_ludovicianus","Urocitellus_richardson
ii",
               "Marmota_marmota","Lariscus_insignis","Rhinosciurus_laticaudat
us")

#subset tree per locomotion
Arb_Tree<-drop.tip(tree_squirrel,tree_squirrel$tip.label[-match(Arb_species,
tree_squirrel$tip.label)])
Fos_Tree<-drop.tip(tree_squirrel,tree_squirrel$tip.label[-match(Fos_species,
tree_squirrel$tip.label)])
Gli_Tree<-drop.tip(tree_squirrel,tree_squirrel$tip.label[-match(Gli_species,
tree_squirrel$tip.label)])
Sca_Tree<-drop.tip(tree_squirrel,tree_squirrel$tip.label[-match(Sca_species,
tree_squirrel$tip.label)])
Ter_Tree<-drop.tip(tree_squirrel,tree_squirrel$tip.label[-match(Ter_species,
tree_squirrel$tip.label)])

#Create model PGLS regression line for each locomotion
Arbline_Br_B <-gls(OB ~ Body.mg, correlation=corMartins (1,phy=Arb_Tree), dat
a=Arb)

Fosline_Br_B <-gls(OB ~ Body.mg, correlation=corMartins (1,phy=Fos_Tree), dat
a=Fos)

Gliline_Br_B <-gls(OB ~ Body.mg, correlation=corMartins (1,phy=Gli_Tree), dat
a=Gli)
```

```
Scaline_Br_B <-gls(OB ~ Body.mg, correlation=corMartins (1,phy=Sca_Tree), dat
a=Sca)

Terline_Br_B <-gls(OB ~ Body.mg, correlation=corMartins (1,phy=Ter_Tree), dat
a=Ter)

#Prepare PGLS for each locomotor mode
pgls.fit.Arb <- predict(Arbline_Br_B)
predframe.Arb <- with(Arb, data.frame(Species, Ecology, Body.mg, OB = pgls.fi
t.Arb))

pgls.fit.Fos <- predict(Fosline_Br_B)
predframe.Fos <- with(Fos, data.frame(Species, Ecology, Body.mg, OB = pgls.fi
t.Fos))

pgls.fit.Gli <- predict(Gliline_Br_B)
predframe.Gli <- with(Gli, data.frame(Species, Ecology, Body.mg, OB = pgls.fi
t.Gli))

pgls.fit.Sca <- predict(Scaline_Br_B)
predframe.Sca <- with(Sca, data.frame(Species, Ecology, Body.mg, OB = pgls.fi
t.Sca))

pgls.fit.Ter <- predict(Terline_Br_B)
predframe.Ter <- with(Ter, data.frame(Species, Ecology, Body.mg, OB = pgls.fi
t.Ter))

#Make graph with PGLS corrected regressions
ggplot(squirrel.data, aes(Body.mg, OB, color = Ecology)) +
  geom_point(data = dplyr::filter(squirrel.data, Ecology == "Arboreal"),
             size = 2, aes(color = "#0EAF28")) +
  geom_point(data = dplyr::filter(squirrel.data, Ecology == "Fossorial"),
             size = 2, aes(color = "#975822")) +
  geom_point(data = dplyr::filter(squirrel.data, Ecology == "Glider"),
             size = 2, aes(color = "#73DAF3")) +
  geom_point(data = dplyr::filter(squirrel.data, Ecology == "Scansorial"),
             size = 2, aes(color = "#F31616")) +
  geom_point(data = dplyr::filter(squirrel.data, Ecology == "Terrestrial"),
             size = 2, aes(color = "#F3B116")) +
  geom_line(data = dplyr::filter(predframe.Arb, Ecology == "Arboreal"), color
= "#0EAF28",
            linetype = 1.5) +
  geom_line(data = dplyr::filter(predframe.Fos, Ecology == "Fossorial"), colo
r = "#975822",
            linetype = 1.5) +
  geom_line(data = dplyr::filter(predframe.Gli, Ecology == "Glider"), color =
"#73DAF3",
            linetype = 1.5) +
  geom_line(data = dplyr::filter(predframe.Sca, Ecology == "Scansorial"), col
or = "#F31616",
```

```
            linetype = 1.5) +
  geom_line(data = dplyr::filter(predframe.Ter, Ecology == "Terrestrial"), co
lor = "#F3B116",
            linetype = 1.5) +
  theme_minimal() +
  #theme(legend.position = "top") +
  scale_color_manual(name = "", values = c("#0EAF28","#73DAF3","#975822", "#F
31616", "#F3B116"),labels = c("Arboreal",
            "Glider","Fossorial", "Scansorial", "Terrestrial")) +
  theme(axis.text = element_text(size = 12), axis.title = element_text(size =
12,
  face = "bold")) +
  labs(x = "log(Body mass)", y = "log(Olfactory bulb volume)") +
  geom_text(data = dplyr::filter(squirrel.data, Ecology == "Arboreal"), color
= "#0EAF28",
            aes(label = abbreviation), hjust = -0.3, vjust = 1.1)+
  geom_text(data = dplyr::filter(squirrel.data, Ecology == "Fossorial"), colo
r = "#975822",
            aes(label = abbreviation), hjust = -0.3, vjust = 1.1) +
  geom_text(data = dplyr::filter(squirrel.data, Ecology == "Glider"), color =
"#73DAF3",
            aes(label = abbreviation), hjust = -0.3, vjust = 1.1) +
  geom_text(data = dplyr::filter(squirrel.data, Ecology == "Scansorial"), col
or = "#F31616",
            aes(label = abbreviation), hjust = -0.3, vjust = 1.1) +
  geom_text(data = dplyr::filter(squirrel.data, Ecology == "Terrestrial"), co
lor = "#F3B116",
            aes(label = abbreviation), hjust = -0.3, vjust = 1.1)
```

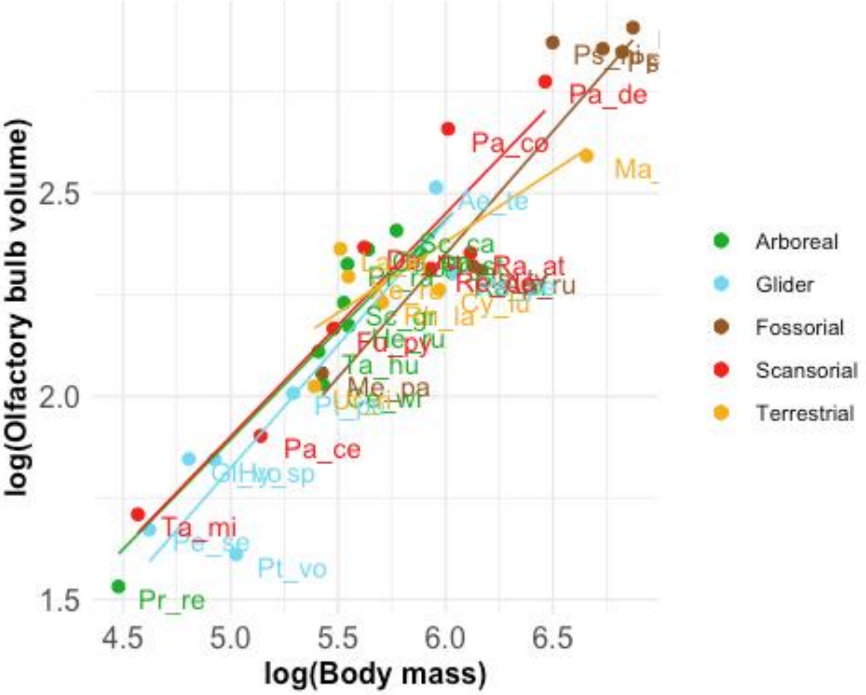

```r
# 5. PGLS regression for petrosal lobule volume vs. body mass

# Code - Can body mass and locomotion predict petrosal lobule size?

library(phytools) #open tree

## Loading required package: ape

## Loading required package: maps

library(ggplot2) #plots
library(nlme) # GLS analysis
library(RRPP) #pairwise comparisons



#directory
setwd("~/Desktop/Squirrel_June_8_2020/Code")

#Import squirrel data
squirrel.data1<-read.csv("squirrels_PEQ_res.csv", header=T)

#Delete a specific row and taxon in tree
squirrel.data<-squirrel.data1[-c(23),]

#Import tree
tree_squirrel1<-read.newick("Calibrated_tree_meng")

#delete taxa
tree_squirrel<-drop.tip(tree_squirrel1, c("Petinomys_setosus"))

#Transform data to log10
squirrel.data$Petrosal_lobule_volume_mm3<-log10(squirrel.data$Petrosal_lobule
_volume_mm3)
names(squirrel.data)[names(squirrel.data) == "Petrosal_lobule_volume_mm3"] <-
"PL"

squirrel.data$Body_mass_mg<-log10(squirrel.data$Body_mass_mg)
names(squirrel.data)[names(squirrel.data) == "Body_mass_mg"] <- "Body.mg"

#Select other variables
Locomotion<-squirrel.data$Locomotion
abbreviation<-squirrel.data$abbreviation

##########################  Analyses -- OLS ###############################

# Look at the correlation among data point by Family
ggplot(squirrel.data, aes(Body.mg, PL, color = Family)) +
  theme_light() + theme(legend.position = "top") +
  geom_point(data = dplyr::filter(squirrel.data, Family == "Sciuridae"), shap
e = 16, size = 3,
```

```
              aes(color = "#4DBBD5FF")) +
  geom_point(data = dplyr::filter(squirrel.data, Family == "Aplodontidae"), s
hape = 16, size = 3,
              aes(color = "#E64B35FF")) +
  geom_point(data = dplyr::filter(squirrel.data, Family == "Ischyromyidae"),
shape = 16, size = 3,
              aes(color = "#3C5488FF")) +
  scale_color_manual(name = "", values = c("#3C5488FF","#4DBBD5FF","#E64B35FF
"),labels = c("Ischyromyidae","Sciuridae", "Aplodontidae")) +
  labs(x = "log(Body mass)", y = "log(Petrosal lobule volume)") +
  geom_text(data = dplyr::filter(squirrel.data, Family == "Sciuridae"), color
= "#4DBBD5FF",
             aes(label = abbreviation), hjust = -0.3, vjust = 1.1) +
  geom_text(data = dplyr::filter(squirrel.data, Family == "Ischyromyidae"), c
olor = "#3C5488FF",
             aes(label = abbreviation), hjust = -0.3, vjust = 1.1) +
  geom_text(data = dplyr::filter(squirrel.data, Family == "Aplodontidae"), co
lor = "#E64B35FF",
             aes(label = abbreviation), hjust = -0.3, vjust = 1.1)
```

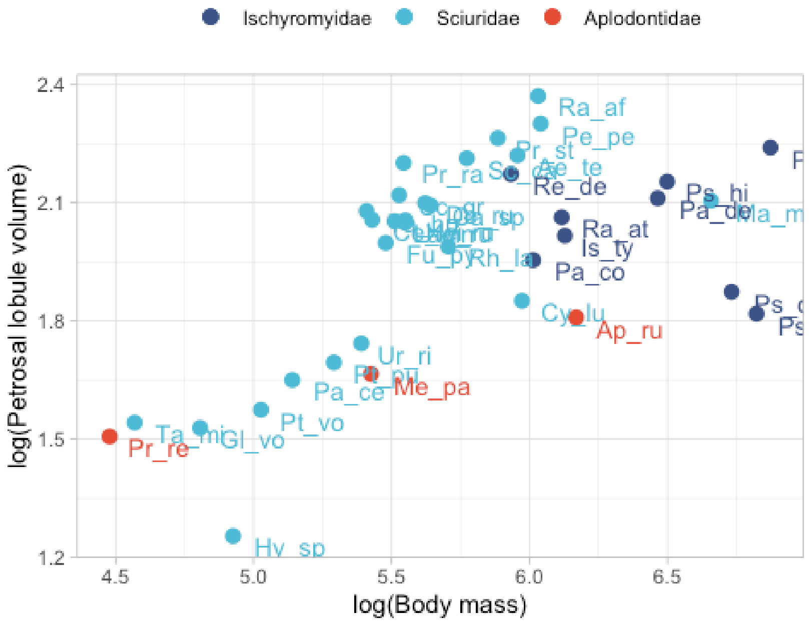


```
#Model using OLS (no phylogeny)
m.ols<-gls(PL ~ Body.mg + Locomotion, data=squirrel.data, method="ML")
m.ols.body<-gls(PL ~ Body.mg + Locomotion*Body.mg, data=squirrel.data, method
="ML")
m.ols.loco<-gls(PL ~ Body.mg*Locomotion, data=squirrel.data, method="ML") #sa
me as above

#Chosing the model to use
anova(m.ols, m.ols.body, m.ols.loco) # m.ols.body (and m.ols.loco) with Lowes
t AIC
```

```
##              Model df       AIC       BIC   logLik   Test L.Ratio p-value
## m.ols            1  4 -14.91217 -8.468495 11.45608
## m.ols.body       2  5 -17.28480 -9.230207 13.64240 1 vs 2 4.37263  0.0365
## m.ols.loco       3  5 -17.28480 -9.230207 13.64240
```

```
summary(m.ols.body)
```

```
## Generalized least squares fit by maximum likelihood
##   Model: PL ~ Body.mg + Locomotion * Body.mg
##   Data: squirrel.data
##        AIC       BIC   logLik
##   -17.2848 -9.230207 13.6424
##
## Coefficients:
##                         Value Std.Error   t-value p-value
## (Intercept)        -1.2004745 0.7889029 -1.521701  0.1376
## Body.mg             0.5930053 0.1403755  4.224422  0.0002
## Locomotion          0.4245065 0.2478528  1.712737  0.0961
## Body.mg:Locomotion -0.0877260 0.0431165 -2.034628  0.0500
##
##  Correlation:
##                    (Intr) Bdy.mg Locmtn
## Body.mg            -0.997
## Locomotion         -0.922  0.913
## Body.mg:Locomotion  0.930 -0.927 -0.996
##
## Standardized residuals:
##        Min         Q1        Med         Q3        Max
## -2.6530897 -0.8346858  0.2109274  0.6114770  1.6741931
##
## Residual standard error: 0.1673527
## Degrees of freedom: 37 total; 33 residual
```

```
#Residuals vs fitted plot
plot(fitted(m.ols.body), residuals(m.ols.body))
abline(0,0) #pattern visible so not good to use OLS
```

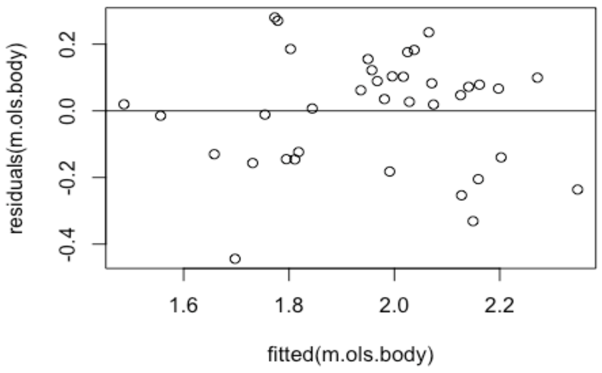

```
# Plot residuals ordered "by phylogeny" (Goldbogen et al., 2019; Supp. p.58)
is_tip <- tree_squirrel$edge[,2]<=length(tree_squirrel$tip.label)
ordered_tips <- tree_squirrel$edge[is_tip,2] # extract the order of tree tips
oj <- residuals(m.ols.body)
tl <- tree_squirrel$tip.label[ordered_tips] # check order in tree to put them
in same order in csv. file

#Plot residuals against phylogeny
Spe<-squirrel.data$Sp
ggplot(squirrel.data, aes(x=Spe, y = oj, color = Family))+
  theme_light() + theme(legend.position = "top") +
  labs(x = "Species index", y = "OLS residuals") +
  geom_point() +
  geom_abline(intercept = 0, slope = 0) ## pattern, i.e., green group lower c
ompared to blue group
```

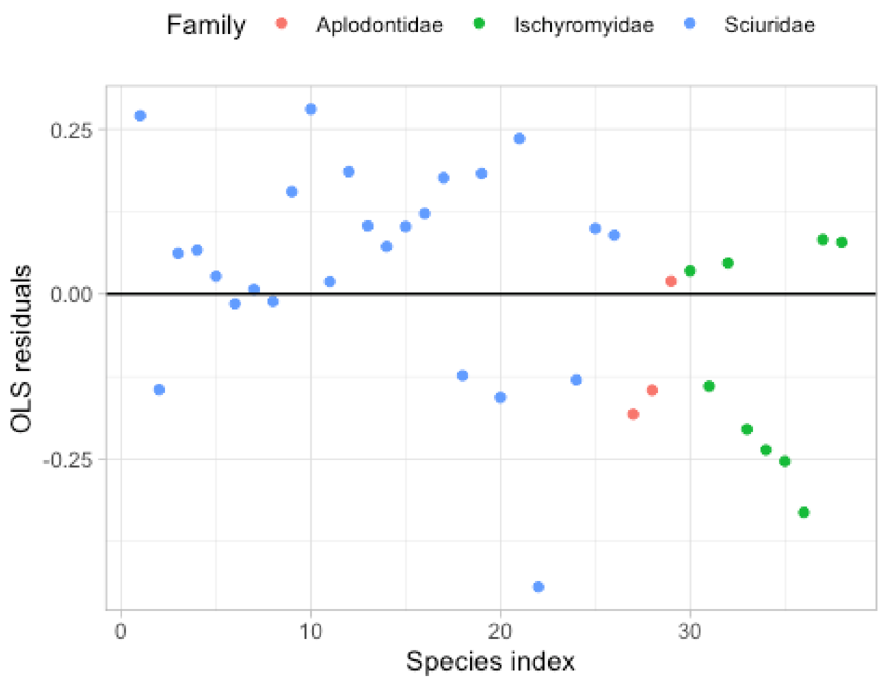


```
##########################  Analyses -- PGLS ###############################

### Select PGLS model
# Model with locomotion only
Lambda<-gls(PL ~ Body.mg + Locomotion, data=squirrel.data,
            correlation=corPagel(value=1,phy=tree_squirrel), method="ML")

Brownian<-gls(PL ~ Body.mg + Locomotion, data=squirrel.data,
              correlation=corBrownian(1,phy=tree_squirrel), method="ML")

OU<-gls(PL ~ Body.mg + Locomotion, data=squirrel.data,
        correlation=corMartins(1,phy=tree_squirrel), method="ML")
```

```
Blomberg<-gls(PL ~ Body.mg + Locomotion, data=squirrel.data,
              correlation=corBlomberg(1.5,phy=tree_squirrel,fixed = TRUE), me
thod="ML") #if 1, this is a Brownian model

# Model with interaction betwenn Locomotion and Body mass
Lambda1<-gls(PL ~ Body.mg + Locomotion*Body.mg, data=squirrel.data,
             correlation=corPagel(value=1,phy=tree_squirrel), method="ML")

Brownian1<-gls(PL ~ Body.mg + Locomotion*Body.mg, data=squirrel.data,
               correlation=corBrownian(1,phy=tree_squirrel), method="ML")

OU1<-gls(PL ~ Body.mg + Locomotion*Body.mg, data=squirrel.data,
         correlation=corMartins(1,phy=tree_squirrel, fixed = TRUE), method="M
L")

Blomberg1<-gls(PL ~ Body.mg + Locomotion*Body.mg, data=squirrel.data,
               correlation=corBlomberg(1.5,phy=tree_squirrel,fixed = TRUE), m
ethod="ML") #if 1, this is a Brownian model

#Chosing the best model using AIC
anova(Lambda,Brownian,OU, Blomberg) # Lambda is the best

##          Model df        AIC       BIC    logLik   Test  L.Ratio p-value
## Lambda       1  5 -16.899581 -8.844992 13.449791
## Brownian     2  4   2.141223  8.584895  2.929389 1 vs 2 21.04080  <.0001
## OU           3  5 -13.332192 -5.277603 11.666096 2 vs 3 17.47341  <.0001
## Blomberg     4  4   4.668787 11.112459  1.665606 3 vs 4 20.00098  <.0001

anova(Lambda1,Brownian1,OU1, Blomberg1) # Lambda1 is the best

##           Model df        AIC        BIC    logLik   Test  L.Ratio p-value
## Lambda1       1  6 -20.372279 -10.706771 16.186139
## Brownian1     2  5  -3.136322   4.918267  6.568161 1 vs 2 19.23596  <.0001
## OU1           3  5 -17.863287  -9.808697 13.931643
## Blomberg1     4  5  -0.546237   7.508352  5.273119

anova(Lambda,Lambda1) # Lambda1 is the best model overall

##         Model df       AIC        BIC   logLik   Test  L.Ratio p-value
## Lambda      1  5 -16.89958  -8.844992 13.44979
## Lambda1     2  6 -20.37228 -10.706771 16.18614 1 vs 2 5.472698  0.0193

summary(Lambda1) #Final model

## Generalized least squares fit by maximum likelihood
##   Model: PL ~ Body.mg + Locomotion * Body.mg
##   Data: squirrel.data
##         AIC       BIC   logLik
##   -20.37228 -10.70677 16.18614
##
## Correlation Structure: corPagel
##  Formula: ~1
```

```
##  Parameter estimate(s):
##    lambda
## 0.5351408
##
## Coefficients:
##                           Value Std.Error   t-value p-value
## (Intercept)          -1.9860939 0.7221237 -2.750351  0.0096
## Body.mg               0.7048144 0.1261742  5.586042  0.0000
## Locomotion            0.4462055 0.2253234  1.980290  0.0561
## Body.mg:Locomotion   -0.0912935 0.0394500 -2.314155  0.0270
##
##  Correlation:
##                      (Intr) Bdy.mg Locmtn
## Body.mg              -0.992
## Locomotion           -0.877  0.884
## Body.mg:Locomotion    0.886 -0.899 -0.996
##
## Standardized residuals:
##        Min         Q1        Med          Q3        Max
## -1.5771507  0.3136526  0.9124637  1.4065291  2.3278408
##
## Residual standard error: 0.1887506
## Degrees of freedom: 37 total; 33 residual

#Lambda = 0.54 (brain size shows some phylogenetic signal)
#Coefficients: p-value interpretation:
#Locomotion alone does not predict brain size (p-value = 0.056)
#Body mass alone and the interaction between Body mass and locomotion
#can predict brain size (p-value = 0 and 0.027)

################# post ad-hoc test on locomotion ################

#tutorial RRPP: https://cran.r-project.org/web/packages/RRPP/vignettes/Using.
RRPP.html

#Import squirrel data - to run the following
squirrel.data1<-read.csv("squirrels_PEQ_res.csv", header=T)
squirrel.data<-squirrel.data1[-c(23),]
tree_squirrel1<-read.newick("Calibrated_tree_meng")
tree_squirrel<-drop.tip(tree_squirrel1, c("Petinomys_setosus"))

PL<-log10(squirrel.data$Petrosal_lobule_volume_mm3)
Body<-log10(squirrel.data$Body_mass_mg)
Ecology<-squirrel.data$Ecology

#Pairwise comparisons - non-phylogenetic! (Weisbecker et al., 2019; line 552
in Analyses)
interaction_frame <- rrpp.data.frame(pl=PL,body=Body,loco=Ecology)
fit<-lm.rrpp(pl~body+loco*body,SS.type = c("I"),data=interaction_frame)
```

```r
summary(fit, formula = FALSE)
```

```
##
## Linear Model fit with lm.rrpp
##
## Number of observations: 37
## Number of dependent variables: 1
## Data space dimensions: 1
## Sums of Squares and Cross-products: Type I
## Number of permutations: 1000
##
## Full Model Analysis of Variance
##
##     Df Residual Df      SS Residual SS       Rsq        F Z (from F)
## fit  9          27 2.04132    0.437193 0.8236068 14.00745   4.772388
##            Pr(>F)
## fit 0.0006666667
##
##
## Redundancy Analysis (PCA on fitted values and residuals)
##
##                Trace Proportion Rank
## Fitted    0.05670335  0.8236068    1
## Residuals 0.01214425  0.1763932    1
## Total     0.06884760  1.0000001    1
##
## Eigenvalues
##
##                  PC1
## Fitted    0.05670335
## Residuals 0.01214425
## Total     0.06884760
```

```r
anova(fit)
```

```
##
## Analysis of Variance, using Residual Randomization
## Permutation procedure: Randomization of null model residuals
## Number of permutations: 1000
## Estimation method: Ordinary Least Squares
## Sums of Squares and Cross-products: Type I
## Effect sizes (Z) based on F distributions
##
##           Df      SS      MS     Rsq       F      Z Pr(>F)
## body       1 0.89376 0.89376 0.36060 55.1963 2.3221  0.001 **
## loco       4 0.81243 0.20311 0.32779 12.5434 3.1690  0.001 **
## body:loco  4 0.33514 0.08378 0.13522  5.1743 2.1692  0.004 **
## Residuals 27 0.43719 0.01619 0.17639
## Total     36 2.47851
## ---
```

```
## Signif. codes:  0 '***' 0.001 '**' 0.01 '*' 0.05 '.' 0.1 ' ' 1
##
## Call: lm.rrpp(f1 = pl ~ body + loco * body, SS.type = c("I"), data = inter
action_frame)

Interactions <- pairwise(fit,covariate=interaction_frame$body,
                         groups=interaction_frame$loco)

summary(Interactions, test.type="dist") # shows which locomotor modes are sig
nificantly different

##
## Pairwise comparisons
##
## Groups: Arboreal Fossorial Glider Scansorial Terrestrial
##
## RRPP: 1000 permutations
##
## Slopes (vectors of variate change per one unit of covariate change, by gro
up):
## Vectors hidden (use show.vectors = TRUE to view)
##
## Slope vector lengths
##    Arboreal   Fossorial      Glider  Scansorial Terrestrial
##   0.5395187   0.2297849   0.7407755   0.3259892   0.1311747
##
## Pairwise absolute difference (d) between vector lengths, plus statistics
##                                   d UCL (95%)          Z Pr > d
## Arboreal:Fossorial     0.30973389 0.3281145  1.7842155  0.064
## Arboreal:Glider        0.20125676 0.3280727  0.6536439  0.239
## Arboreal:Scansorial    0.21352954 0.2971918  0.9997935  0.166
## Arboreal:Terrestrial   0.40834408 0.3687880  2.3677856  0.026
## Fossorial:Glider       0.51099064 0.3320613  3.6173288  0.001
## Fossorial:Scansorial   0.09620434 0.2813910 -0.2446544  0.518
## Fossorial:Terrestrial  0.09861019 0.3387969 -0.4643836  0.606
## Glider:Scansorial      0.41478630 0.3023689  3.2475605  0.004
## Glider:Terrestrial     0.60960084 0.3718727  3.9951789  0.002
## Scansorial:Terrestrial 0.19481454 0.3281320  0.6124696  0.260

# Based on the fit model, how does brain size predicted to vary for each loco
motor category? (what I understand this does)
sizeDF <- data.frame(loco = c("Arboreal", "Scansorial","Glider","Terrestrial"
,"Fossorial"))
rownames(sizeDF) <- c("Arboreal", "Scansorial","Glider","Terrestrial","Fossor
ial")
sizePreds <- predict(fit,sizeDF)

##
## Warning: Not all variables in model accounted for in newdata.
## Missing variables will be averaged from observed data for prediction.
```

```
plot(sizePreds)
```

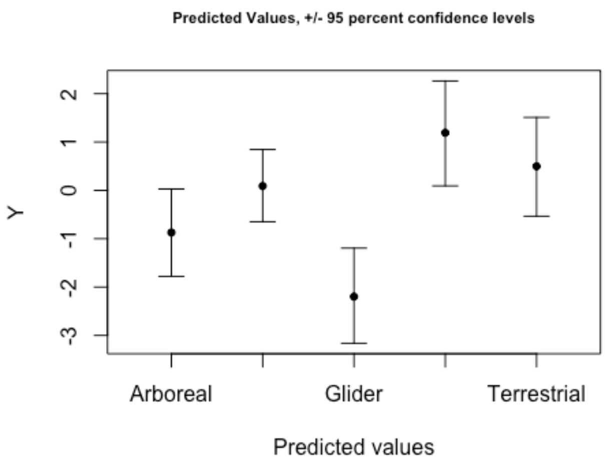


```
################ Make graph with PGLS regression lines for each locomotion ##
#########

#Import data
squirrel.data1<-read.csv("squirrels_PEQ_res.csv", header=T)
squirrel.data<-squirrel.data1[-c(23),]

#Import tree
tree_squirrel1<-read.newick("Calibrated_tree_meng")
tree_squirrel<-drop.tip(tree_squirrel1, c("Petinomys_setosus"))

#Transform data to log10
squirrel.data$Petrosal_lobule_volume_mm3<-log10(squirrel.data$Petrosal_lobule
_volume_mm3)
names(squirrel.data)[names(squirrel.data) == "Petrosal_lobule_volume_mm3"] <-
"PL"
squirrel.data$Body_mass_mg<-log10(squirrel.data$Body_mass_mg)
names(squirrel.data)[names(squirrel.data) == "Body_mass_mg"] <- "Body.mg"

#subset dataset per locomotion to use in model
Arb<- squirrel.data[ which(squirrel.data$Ecology=='Arboreal'), ]
Fos<- squirrel.data[ which(squirrel.data$Ecology=='Fossorial'), ]
Gli<- squirrel.data[ which(squirrel.data$Ecology=='Glider'), ]
Sca<- squirrel.data[ which(squirrel.data$Ecology=='Scansorial'), ]
Ter<- squirrel.data[ which(squirrel.data$Ecology=='Terrestrial'), ]

#Find taxa with specific locomotion
Arb_sp<-subset(squirrel.data, Ecology=="Arboreal", select=Species)
Fos_sp<-subset(squirrel.data, Ecology=="Fossorial", select=Species)
Gli_sp<-subset(squirrel.data, Ecology=="Glider", select=Species)
Sca_sp<-subset(squirrel.data, Ecology=="Scansorial", select=Species)
Ter_sp<-subset(squirrel.data, Ecology=="Terrestrial", select=Species)
```

```
#Select taxa per locomotion for the tree
Arb_species<-c("Protoxerus_stangeri","Heliosciurus_rufobrachium","Callosciuru
s_sp",
               "Sciurus_carolinensis","Sciurus_granatensis","Tamiasciurus_hud
sonicus",
               "Protosciurus_rachelae","Ratufa_affinis","Cedromus_wilsoni",
               "Prosciurus_relictus")

Fos_species<-c("Aplodontia_rufa","Mesogaulus_paniensis","Ischyromys_typus",
               "Pseudotomus_oweni","Pseudotomus_petersoni","Pseudotomus_hians
",
               "Pseudotomus_horribilis")

Gli_species<-c("Pteromyscus_pulverulentus","Aeromys_tephromelas","Pteromys_vo
lans",
               "Petaurista_petaurista","Hylopetes_spadiceus","Glaucomys_volan
s")

Sca_species<-c("Paraxerus_cepapi","Funisciurus_pyrropus","Tamias_minimus",
               "Dremomys_rufigenis","Rapamys_atramontis","Reithroparamys_deli
catissimus",
               "Paramys_copei","Paramys_delicatus")

Ter_species<-c("Xerus_rutilus","Cynomys_ludovicianus","Urocitellus_richardson
ii",
               "Marmota_marmota","Lariscus_insignis","Rhinosciurus_laticaudat
us")

#subset tree per locomotion
Arb_Tree<-drop.tip(tree_squirrel,tree_squirrel$tip.label[-match(Arb_species,
tree_squirrel$tip.label)])
Fos_Tree<-drop.tip(tree_squirrel,tree_squirrel$tip.label[-match(Fos_species,
tree_squirrel$tip.label)])
Gli_Tree<-drop.tip(tree_squirrel,tree_squirrel$tip.label[-match(Gli_species,
tree_squirrel$tip.label)])
Sca_Tree<-drop.tip(tree_squirrel,tree_squirrel$tip.label[-match(Sca_species,
tree_squirrel$tip.label)])
Ter_Tree<-drop.tip(tree_squirrel,tree_squirrel$tip.label[-match(Ter_species,
tree_squirrel$tip.label)])

#Create model PGLS regression line for each locomotion
Arbline_Br_B <-gls(PL ~ Body.mg, correlation=corPagel (1,phy=Arb_Tree), data=
Arb)

Fosline_Br_B <-gls(PL ~ Body.mg, correlation=corPagel (1,phy=Fos_Tree), data=
Fos)

Gliline_Br_B <-gls(PL ~ Body.mg, correlation=corPagel (1,phy=Gli_Tree), data=
Gli)
```

```r
Scaline_Br_B <-gls(PL ~ Body.mg, correlation=corPagel (1,phy=Sca_Tree), data=
Sca)

Terline_Br_B <-gls(PL ~ Body.mg, correlation=corPagel (1,phy=Ter_Tree), data=
Ter)

#Prepare PGLS for each locomotor mode
pgls.fit.Arb <- predict(Arbline_Br_B)
predframe.Arb <- with(Arb, data.frame(Species, Ecology, Body.mg, PL = pgls.fi
t.Arb))

pgls.fit.Fos <- predict(Fosline_Br_B)
predframe.Fos <- with(Fos, data.frame(Species, Ecology, Body.mg, PL = pgls.fi
t.Fos))

pgls.fit.Gli <- predict(Gliline_Br_B)
predframe.Gli <- with(Gli, data.frame(Species, Ecology, Body.mg, PL = pgls.fi
t.Gli))

pgls.fit.Sca <- predict(Scaline_Br_B)
predframe.Sca <- with(Sca, data.frame(Species, Ecology, Body.mg, PL = pgls.fi
t.Sca))

pgls.fit.Ter <- predict(Terline_Br_B)
predframe.Ter <- with(Ter, data.frame(Species, Ecology, Body.mg, PL = pgls.fi
t.Ter))

#Make graph with PGLS corrected regressions
ggplot(squirrel.data, aes(Body.mg, PL, color = Ecology)) +
  geom_point(data = dplyr::filter(squirrel.data, Ecology == "Arboreal"),
             size = 2, aes(color = "#0EAF28")) +
  geom_point(data = dplyr::filter(squirrel.data, Ecology == "Fossorial"),
             size = 2, aes(color = "#975822")) +
  geom_point(data = dplyr::filter(squirrel.data, Ecology == "Glider"),
             size = 2, aes(color = "#73DAF3")) +
  geom_point(data = dplyr::filter(squirrel.data, Ecology == "Scansorial"),
             size = 2, aes(color = "#F31616")) +
  geom_point(data = dplyr::filter(squirrel.data, Ecology == "Terrestrial"),
             size = 2, aes(color = "#F3B116")) +
  geom_line(data = dplyr::filter(predframe.Arb, Ecology == "Arboreal"), color
= "#0EAF28",
            linetype = 1.5) +
  geom_line(data = dplyr::filter(predframe.Fos, Ecology == "Fossorial"), colo
r = "#975822",
            linetype = 1.5) +
  geom_line(data = dplyr::filter(predframe.Gli, Ecology == "Glider"), color =
"#73DAF3",
            linetype = 1.5) +
  geom_line(data = dplyr::filter(predframe.Sca, Ecology == "Scansorial"), col
or = "#F31616",
```

```
            linetype = 1.5) +
  geom_line(data = dplyr::filter(predframe.Ter, Ecology == "Terrestrial"), co
lor = "#F3B116",
            linetype = 1.5) +
  theme_minimal() +
  #theme(legend.position = "top") +
  scale_color_manual(name = "", values = c("#0EAF28","#73DAF3","#975822", "#F
31616", "#F3B116"),labels = c("Arboreal",
            "Glider","Fossorial", "Scansorial", "Terrestrial")) +
  theme(axis.text = element_text(size = 12), axis.title = element_text(size =
12,
              face = "bold")) +
  labs(x = "log(Body mass)", y = "log(Petrosal lobule volume)") +
  geom_text(data = dplyr::filter(squirrel.data, Ecology == "Arboreal"), color
= "#0EAF28",
            aes(label = abbreviation), hjust = -0.3, vjust = 1.1)+
  geom_text(data = dplyr::filter(squirrel.data, Ecology == "Fossorial"), colo
r = "#975822",
            aes(label = abbreviation), hjust = -0.3, vjust = 1.1) +
  geom_text(data = dplyr::filter(squirrel.data, Ecology == "Glider"), color =
"#73DAF3",
            aes(label = abbreviation), hjust = -0.3, vjust = 1.1) +
  geom_text(data = dplyr::filter(squirrel.data, Ecology == "Scansorial"), col
or = "#F31616",
            aes(label = abbreviation), hjust = -0.3, vjust = 1.1) +
  geom_text(data = dplyr::filter(squirrel.data, Ecology == "Terrestrial"), co
lor = "#F3B116",
            aes(label = abbreviation), hjust = -0.3, vjust = 1.1)
```

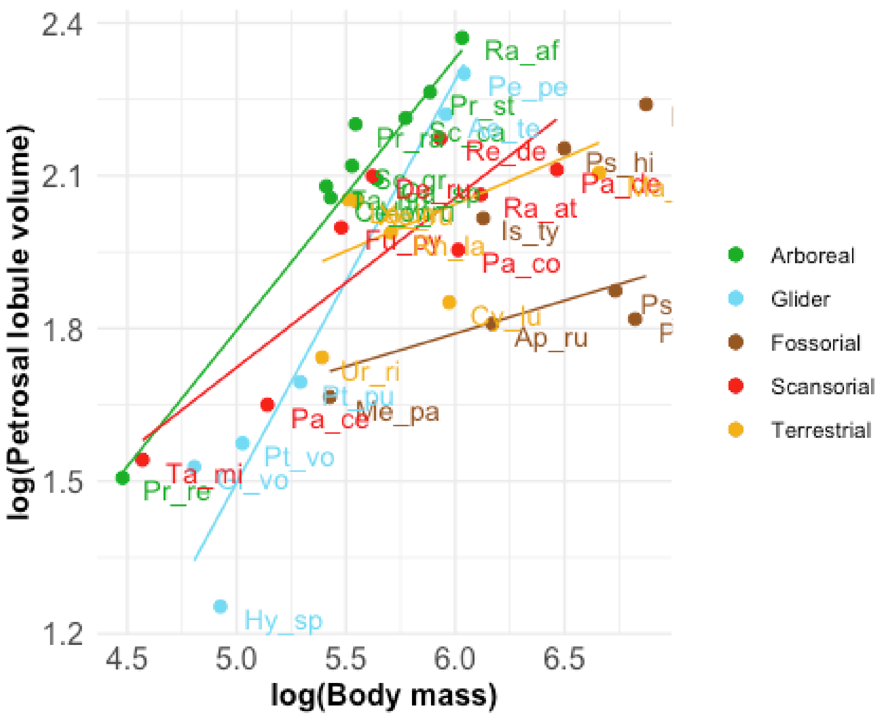

#6.PGLS regression for olfactory bulb vs. endocranial volume

```
# Can endocranial volume and locomotion predict olfactory bulb size?

library(phytools) #open tree

## Loading required package: ape

## Loading required package: maps

library(ggplot2) #plots
library(nlme) # GLS analysis
library(RRPP) #pairwise comparisons

#directory
setwd("~/Desktop/Squirrel_June_8_2020/Code")

#Import squirrel data
squirrel.data<-read.csv("squirrels_PEQ_res.csv", header=T)

#Import tree
tree_squirrel<-read.newick("Calibrated_tree_meng")

#Transform data to log10
squirrel.data$Olfactory_bulb_volume_mm3<-log10(squirrel.data$Olfactory_bulb_v
olume_mm3)
names(squirrel.data)[names(squirrel.data) == "Olfactory_bulb_volume_mm3"] <-
"OB"

squirrel.data$Brain_volume_mm3<-log10(squirrel.data$Brain_volume_mm3)
names(squirrel.data)[names(squirrel.data) == "Brain_volume_mm3"] <- "Brain.mg
"

#Select other variables
Locomotion<-squirrel.data$Locomotion
abbreviation<-squirrel.data$abbreviation

##########################  Analyses -- OLS ###############################

# Look at the correlation among data point by Family
ggplot(squirrel.data, aes(Brain.mg, OB, color = Family)) +
  theme_light() + theme(legend.position = "top") +
  geom_point(data = dplyr::filter(squirrel.data, Family == "Sciuridae"), shap
e = 16, size = 3,
             aes(color = "#4DBBD5FF")) +
  geom_point(data = dplyr::filter(squirrel.data, Family == "Aplodontidae"), s
hape = 16, size = 3,
             aes(color = "#E64B35FF")) +
  geom_point(data = dplyr::filter(squirrel.data, Family == "Ischyromyidae"),
shape = 16, size = 3,
             aes(color = "#3C5488FF")) +
```

```
  scale_color_manual(name = "", values = c("#3C5488FF","#4DBBD5FF","#E64B35FF
"),labels = c("Ischyromyidae","Sciuridae", "Aplodontidae")) +
  labs(x = "log(Endocranial volume)", y = "log(Olfactory bulb volume)") +
  geom_text(data = dplyr::filter(squirrel.data, Family == "Sciuridae"), color
= "#4DBBD5FF",
            aes(label = abbreviation), hjust = -0.3, vjust = 1.1) +
  geom_text(data = dplyr::filter(squirrel.data, Family == "Ischyromyidae"), c
olor = "#3C5488FF",
            aes(label = abbreviation), hjust = -0.3, vjust = 1.1) +
  geom_text(data = dplyr::filter(squirrel.data, Family == "Aplodontidae"), co
lor = "#E64B35FF",
            aes(label = abbreviation), hjust = -0.3, vjust = 1.1)
```

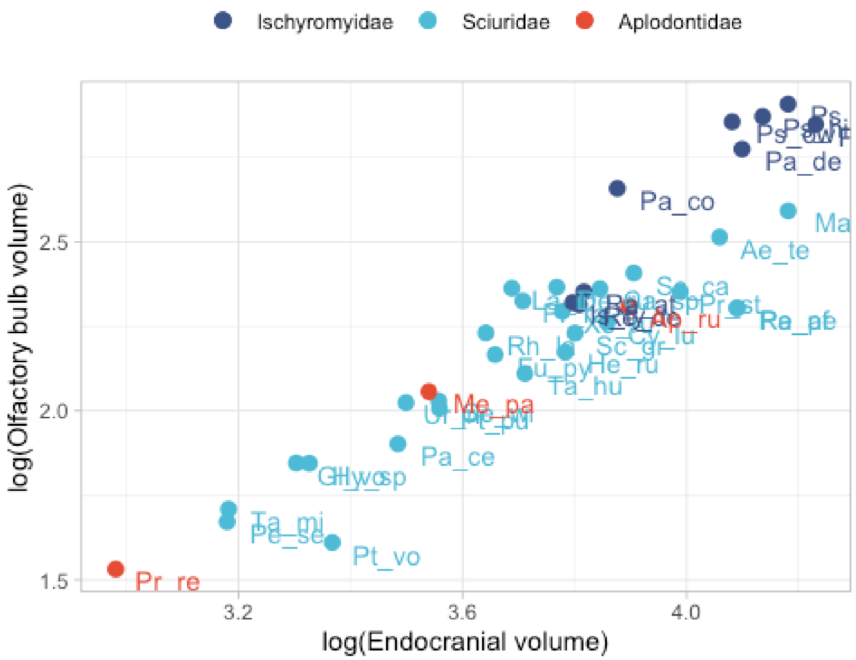


```
#Model using OLS (no phylogeny)
m.ols<-gls(OB ~ Brain.mg + Locomotion, data=squirrel.data, method="ML")
m.ols.body<-gls(OB ~ Brain.mg + Locomotion*Brain.mg, data=squirrel.data, meth
od="ML")
m.ols.loco<-gls(OB ~ Brain.mg*Locomotion, data=squirrel.data, method="ML") #s
ame as above

#Chosing the model to use
anova(m.ols, m.ols.loco) # m.ols with lowest AIC

##            Model df       AIC       BIC   logLik   Test L.Ratio p-value
## m.ols          1  4 -38.94520 -32.39485 23.47260
## m.ols.loco     2  5 -38.47277 -30.28484 24.23638 1 vs 2 1.52757  0.2165

summary(m.ols)
```

```
## Generalized least squares fit by maximum likelihood
##   Model: OB ~ Brain.mg + Locomotion
##   Data: squirrel.data
##        AIC       BIC  logLik
##   -38.9452 -32.39485 23.4726
##
## Coefficients:
##                  Value  Std.Error   t-value p-value
## (Intercept) -1.6621684 0.27298198 -6.088931   0.000
## Brain.mg     1.0304081 0.07340093 14.038080   0.000
## Locomotion   0.0206738 0.01568241  1.318279   0.196
##
##  Correlation:
##            (Intr) Brn.mg
## Brain.mg   -0.984
## Locomotion -0.008 -0.150
##
## Standardized residuals:
##         Min          Q1         Med          Q3         Max
## -2.37557349 -0.52184717  0.06185919  0.48722154  2.18277913
##
## Residual standard error: 0.1304665
## Degrees of freedom: 38 total; 35 residual
```

```r
#Residuals vs fitted plot
plot(fitted(m.ols), residuals(m.ols))
abline(0,0) #pattern visible so not good to use OLS
```

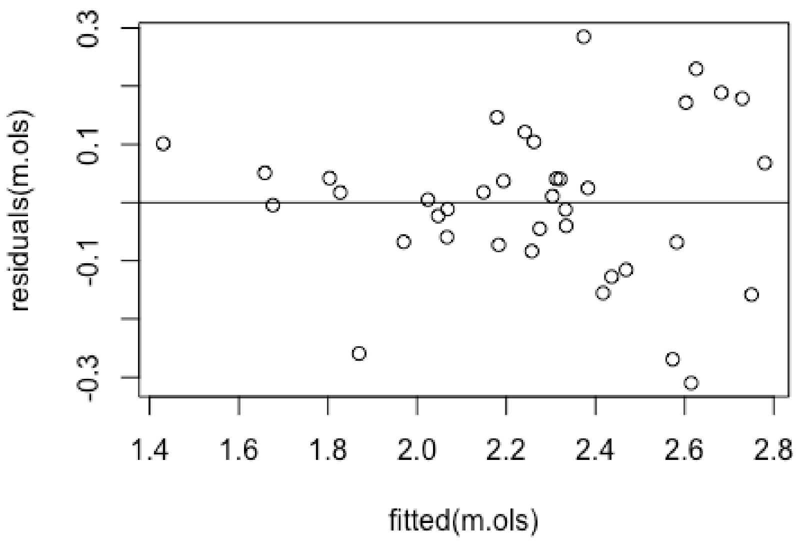

```r
# Plot residuals ordered “by phylogeny” (Goldbogen et al., 2019; Supp. p.58)
is_tip <- tree_squirrel$edge[,2]<=length(tree_squirrel$tip.label)
ordered_tips <- tree_squirrel$edge[is_tip,2] # extract the order of tree tips
oj <- residuals(m.ols)
tl <- tree_squirrel$tip.label[ordered_tips] # check order in tree to put them
in same order in csv. file

#Plot residuals against phylogeny
Spe<-squirrel.data$Sp
ggplot(squirrel.data, aes(x=Spe, y = oj, color = Family))+
  theme_light() + theme(legend.position = "top") +
  labs(x = "Species index", y = "OLS residuals") +
  geom_point() +
  geom_abline(intercept = 0, slope = 0) ## pattern, i.e., green ones are very
high so need PGLS
```

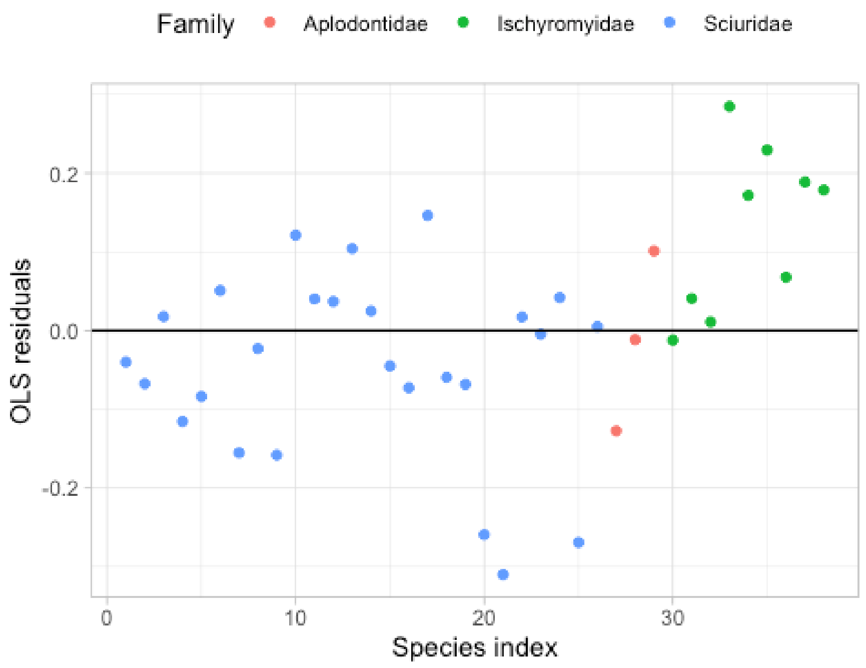


```r
##########################  Analyses -- PGLS ###############################

### Select PGLS model
# Model with locomotion only
Lambda<-gls(OB ~ Brain.mg + Locomotion, data=squirrel.data,
            correlation=corPagel(value=1,phy=tree_squirrel), method="ML")

Brownian<-gls(OB ~ Brain.mg + Locomotion, data=squirrel.data,
              correlation=corBrownian(1,phy=tree_squirrel), method="ML")

OU<-gls(OB ~ Brain.mg + Locomotion, data=squirrel.data,
        correlation=corMartins(1,phy=tree_squirrel,fixed = TRUE), method="ML"
)
```

```
Blomberg<-gls(OB ~ Brain.mg + Locomotion, data=squirrel.data,
              correlation=corBlomberg(1.5,phy=tree_squirrel,fixed = TRUE), me
thod="ML") #if 1, this is a Brownian model

# Model with interaction betwenn Locomotion and Body mass
Lambda1<-gls(OB ~ Brain.mg + Locomotion*Brain.mg, data=squirrel.data,
             correlation=corPagel(value=1,phy=tree_squirrel), method="ML")

Brownian1<-gls(OB ~ Brain.mg + Locomotion*Brain.mg, data=squirrel.data,
               correlation=corBrownian(1,phy=tree_squirrel), method="ML")

OU1<-gls(OB ~ Brain.mg + Locomotion*Brain.mg, data=squirrel.data,
         correlation=corMartins(1,phy=tree_squirrel, fixed = TRUE), method="M
L")

Blomberg1<-gls(OB ~ Brain.mg + Locomotion*Brain.mg, data=squirrel.data,
               correlation=corBlomberg(1.5,phy=tree_squirrel,fixed = TRUE), m
ethod="ML") #if 1, this is a Brownian model

#Chosing the best model using AIC
anova(Lambda,Brownian,OU, Blomberg) # Lambda is the best

##          Model df       AIC       BIC   logLik   Test  L.Ratio p-value
## Lambda       1  5 -50.47906 -42.29113 30.23953
## Brownian     2  4 -40.48840 -33.93805 24.24420 1 vs 2 11.99067   5e-04
## OU           3  4 -44.21400 -37.66366 26.10700
## Blomberg     4  4 -38.25413 -31.70379 23.12707

anova(Lambda1,Brownian1,OU1, Blomberg1)

##           Model df       AIC       BIC   logLik   Test L.Ratio p-value
## Lambda1       1  6 -48.54437 -38.71885 30.27219
## Brownian1     2  5 -39.26877 -31.08084 24.63439 1 vs 2 11.2756   8e-04
## OU1           3  5 -43.00670 -34.81877 26.50335
## Blomberg1     4  5 -37.06952 -28.88159 23.53476

anova(Lambda,Lambda1) #Final model - Lambda

##         Model df       AIC       BIC   logLik   Test    L.Ratio p-value
## Lambda      1  5 -50.47906 -42.29113 30.23953
## Lambda1     2  6 -48.54437 -38.71885 30.27219 1 vs 2 0.06530898  0.7983

summary(Lambda)

## Generalized least squares fit by maximum likelihood
##   Model: OB ~ Brain.mg + Locomotion
##   Data: squirrel.data
##         AIC       BIC   logLik
##   -50.47906 -42.29113 30.23953
##
## Correlation Structure: corPagel
##  Formula: ~1
```

```
##  Parameter estimate(s):
##    lambda
## 0.6898178
##
## Coefficients:
##                  Value  Std.Error   t-value p-value
## (Intercept) -0.9269307 0.24410708 -3.797230  0.0006
## Brain.mg     0.8624954 0.06046361 14.264703  0.0000
## Locomotion   0.0168615 0.01238109  1.361872  0.1819
##
##  Correlation:
##            (Intr) Brn.mg
## Brain.mg   -0.959
## Locomotion -0.016 -0.128
##
## Standardized residuals:
##        Min         Q1        Med         Q3        Max
## -2.8031279 -1.2018700 -0.8596863 -0.3058489  1.3969895
##
## Residual standard error: 0.1489613
## Degrees of freedom: 38 total; 35 residual

#Lambda = 0.69 (OB relative size shows some phylogenetic signal)
#Coefficients: p-value interpretation:
#Locomotion does not predict olfactory bulb size (p-value = 0.18)
#Endocranial volume alone can predict brain size (p-value = 0)

################# post ad-hoc test on locomotion ###############

#tutorial RRPP: https://cran.r-project.org/web/packages/RRPP/vignettes/Using.
RRPP.html

#Import squirrel data - to run pairwise test
squirrel.data<-read.csv("squirrels_PEQ_res.csv", header=T)
OB<-log10(squirrel.data$Olfactory_bulb_volume_mm3)
Brain<-log10(squirrel.data$Brain_volume_mm3)
Ecology<-squirrel.data$Ecology

#Pairwise comparisons - non-phylogenetic! (Weisbecker et al., 2019; line 552
in Analyses)
interaction_frame <- rrpp.data.frame(ob=OB,brain=Brain,loco=Ecology)
fit<-lm.rrpp(ob~brain+loco,SS.type = c("I"),data=interaction_frame)

summary(fit, formula = FALSE)

##
## Linear Model fit with lm.rrpp
##
## Number of observations: 38
## Number of dependent variables: 1
```

```
## Data space dimensions: 1
## Sums of Squares and Cross-products: Type I
## Number of permutations: 1000
##
## Full Model Analysis of Variance
##
##     Df Residual Df       SS Residual SS       Rsq        F Z (from F)  Pr(
>F)
## fit  5          32 4.038719   0.4713258 0.8954942 54.84063   5.509363 0.00
075
##
##
## Redundancy Analysis (PCA on fitted values and residuals)
##
##                Trace Proportion Rank
## Fitted    0.10915457  0.8954942    1
## Residuals 0.01273853  0.1045057    1
## Total     0.12189311  1.0000000    1
##
## Eigenvalues
##
##                  PC1
## Fitted    0.10915457
## Residuals 0.01273853
## Total     0.12189311
```

```
anova(fit) # When phylogeny is NOT taken into account, locomotion predict olf
actory bulb size
```

```
##
## Analysis of Variance, using Residual Randomization
## Permutation procedure: Randomization of null model residuals
## Number of permutations: 1000
## Estimation method: Ordinary Least Squares
## Sums of Squares and Cross-products: Type I
## Effect sizes (Z) based on F distributions
##
##           Df     SS     MS     Rsq        F      Z Pr(>F)
## brain      1 3.8311 3.8311 0.84946 260.1079 3.0347  0.001 **
## loco       4 0.2076 0.0519 0.04603   3.5238 1.6933  0.019 *
## Residuals 32 0.4713 0.0147 0.10451
## Total     37 4.5100
## ---
## Signif. codes:  0 '***' 0.001 '**' 0.01 '*' 0.05 '.' 0.1 ' ' 1
##
## Call: lm.rrpp(f1 = ob ~ brain + loco, SS.type = c("I"), data = interaction
_frame)
```

```
Interactions <- pairwise(fit,covariate=interaction_frame$body,
                         groups=interaction_frame$loco)
```

```r
summary(Interactions, test.type="dist") # shows which locomotor modes are sig
nificantly different

##
## Pairwise comparisons
##
## Groups: Arboreal Fossorial Glider Scansorial Terrestrial
##
## RRPP: 1000 permutations
##
## LS means:
## Vectors hidden (use show.vectors = TRUE to view)
##
## Pairwise distances between means, plus statistics
##                                  d UCL (95%)           Z Pr > d
## Arboreal:Fossorial     0.18080842 0.1333300  2.93455358  0.007
## Arboreal:Glider        0.03751823 0.1355304 -0.45088402  0.595
## Arboreal:Scansorial    0.12226531 0.1266051  1.83471037  0.063
## Arboreal:Terrestrial   0.07604064 0.1337366  0.48821431  0.280
## Fossorial:Glider       0.21832664 0.1640075  2.96251613  0.010
## Fossorial:Scansorial   0.05854311 0.1482756 -0.03505372  0.445
## Fossorial:Terrestrial  0.10476778 0.1579699  0.89226561  0.179
## Glider:Scansorial      0.15978353 0.1348696  2.38963829  0.020
## Glider:Terrestrial     0.11355886 0.1535414  1.07639225  0.160
## Scansorial:Terrestrial 0.04622467 0.1431204 -0.30057658  0.542

# Based on the fit model, how does brain size predicted to vary for each loco
motor category? (what I understand this does)
sizeDF <- data.frame(loco = c("Arboreal", "Scansorial","Glider","Terrestrial"
,"Fossorial"))
rownames(sizeDF) <- c("Arboreal", "Scansorial","Glider","Terrestrial","Fossor
ial")
sizePreds <- predict(fit,sizeDF)

##
## Warning: Not all variables in model accounted for in newdata.
## Missing variables will be averaged from observed data for prediction.

plot(sizePreds)
```

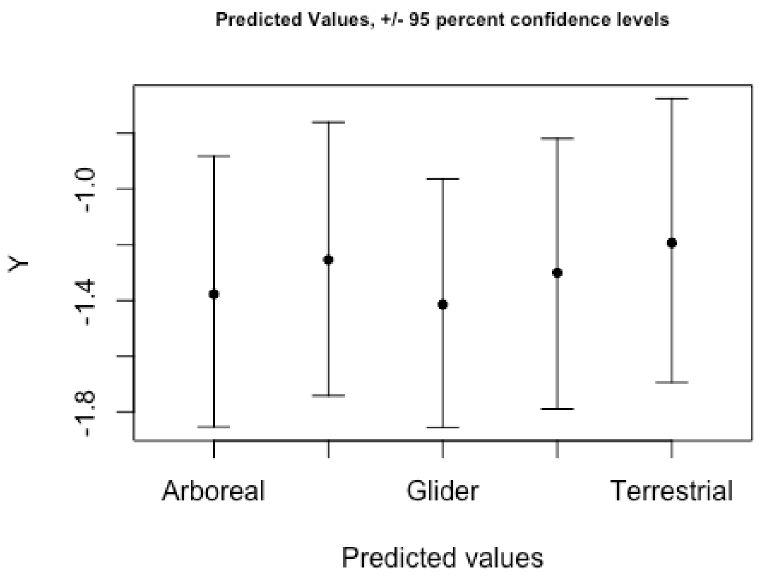


```
################ Make graph with PGLS regression lines for each locomotion ##
#########

#Import squirrel data
squirrel.data<-read.csv("squirrels_PEQ_res.csv", header=T)

#Transform data to log10
squirrel.data$Olfactory_bulb_volume_mm3<-log10(squirrel.data$Olfactory_bulb_v
olume_mm3)
names(squirrel.data)[names(squirrel.data) == "Olfactory_bulb_volume_mm3"] <-
"OB"
squirrel.data$Brain_volume_mm3<-log10(squirrel.data$Brain_volume_mm3)
names(squirrel.data)[names(squirrel.data) == "Brain_volume_mm3"] <- "Brain.mg
"

#subset dataset per locomotion to use in model
Arb<- squirrel.data[ which(squirrel.data$Ecology=='Arboreal'), ]
Fos<- squirrel.data[ which(squirrel.data$Ecology=='Fossorial'), ]
Gli<- squirrel.data[ which(squirrel.data$Ecology=='Glider'), ]
Sca<- squirrel.data[ which(squirrel.data$Ecology=='Scansorial'), ]
Ter<- squirrel.data[ which(squirrel.data$Ecology=='Terrestrial'), ]

#Find taxa with specific locomotion
Arb_sp<-subset(squirrel.data, Ecology=="Arboreal", select=Species)
Fos_sp<-subset(squirrel.data, Ecology=="Fossorial", select=Species)
Gli_sp<-subset(squirrel.data, Ecology=="Glider", select=Species)
Sca_sp<-subset(squirrel.data, Ecology=="Scansorial", select=Species)
Ter_sp<-subset(squirrel.data, Ecology=="Terrestrial", select=Species)

#Select taxa per locomotion for the tree
```

```
Arb_species<-c("Protoxerus_stangeri","Heliosciurus_rufobrachium","Callosciuru
s_sp",
               "Sciurus_carolinensis","Sciurus_granatensis","Tamiasciurus_hud
sonicus",
               "Protosciurus_rachelae","Ratufa_affinis","Cedromus_wilsoni",
               "Prosciurus_relictus")

Fos_species<-c("Aplodontia_rufa","Mesogaulus_paniensis","Ischyromys_typus",
               "Pseudotomus_oweni","Pseudotomus_petersoni","Pseudotomus_hians
",
               "Pseudotomus_horribilis")

Gli_species<-c("Pteromyscus_pulverulentus","Aeromys_tephromelas","Pteromys_vo
lans",
               "Petaurista_petaurista","Hylopetes_spadiceus","Petinomys_setos
us",
               "Glaucomys_volans")

Sca_species<-c("Paraxerus_cepapi","Funisciurus_pyrropus","Tamias_minimus",
               "Dremomys_rufigenis","Rapamys_atramontis","Reithroparamys_deli
catissimus",
               "Paramys_copei","Paramys_delicatus")

Ter_species<-c("Xerus_rutilus","Cynomys_ludovicianus","Urocitellus_richardson
ii",
               "Marmota_marmota","Lariscus_insignis","Rhinosciurus_laticaudat
us")

#subset tree per locomotion
Arb_Tree<-drop.tip(tree_squirrel,tree_squirrel$tip.label[-match(Arb_species,
tree_squirrel$tip.label)])
Fos_Tree<-drop.tip(tree_squirrel,tree_squirrel$tip.label[-match(Fos_species,
tree_squirrel$tip.label)])
Gli_Tree<-drop.tip(tree_squirrel,tree_squirrel$tip.label[-match(Gli_species,
tree_squirrel$tip.label)])
Sca_Tree<-drop.tip(tree_squirrel,tree_squirrel$tip.label[-match(Sca_species,
tree_squirrel$tip.label)])
Ter_Tree<-drop.tip(tree_squirrel,tree_squirrel$tip.label[-match(Ter_species,
tree_squirrel$tip.label)])

#Create model PGLS regression line for each locomotion
Arbline_Br_B <-gls(OB ~ Brain.mg, correlation=corPagel (1,phy=Arb_Tree), data
=Arb)

Fosline_Br_B <-gls(OB ~ Brain.mg, correlation=corPagel (1,phy=Fos_Tree), data
=Fos)

Gliline_Br_B <-gls(OB ~ Brain.mg, correlation=corPagel (1,phy=Gli_Tree), data
=Gli)
```

```
Scaline_Br_B <-gls(OB ~ Brain.mg, correlation=corPagel (1,phy=Sca_Tree), data
=Sca)

Terline_Br_B <-gls(OB ~ Brain.mg, correlation=corPagel (1,phy=Ter_Tree), data
=Ter)

#Prepare PGLS for each locomotor mode
pgls.fit.Arb <- predict(Arbline_Br_B)
predframe.Arb <- with(Arb, data.frame(Species, Ecology, Brain.mg, OB = pgls.f
it.Arb))

pgls.fit.Fos <- predict(Fosline_Br_B)
predframe.Fos <- with(Fos, data.frame(Species, Ecology, Brain.mg, OB = pgls.f
it.Fos))

pgls.fit.Gli <- predict(Gliline_Br_B)
predframe.Gli <- with(Gli, data.frame(Species, Ecology, Brain.mg, OB = pgls.f
it.Gli))

pgls.fit.Sca <- predict(Scaline_Br_B)
predframe.Sca <- with(Sca, data.frame(Species, Ecology, Brain.mg, OB = pgls.f
it.Sca))

pgls.fit.Ter <- predict(Terline_Br_B)
predframe.Ter <- with(Ter, data.frame(Species, Ecology, Brain.mg, OB = pgls.f
it.Ter))

#Make graph with PGLS corrected regressions
ggplot(squirrel.data, aes(Brain.mg, OB, color = Ecology)) +
  geom_point(data = dplyr::filter(squirrel.data, Ecology == "Arboreal"),
             size = 2, aes(color = "#0EAF28")) +
  geom_point(data = dplyr::filter(squirrel.data, Ecology == "Fossorial"),
             size = 2, aes(color = "#975822")) +
  geom_point(data = dplyr::filter(squirrel.data, Ecology == "Glider"),
             size = 2, aes(color = "#73DAF3")) +
  geom_point(data = dplyr::filter(squirrel.data, Ecology == "Scansorial"),
             size = 2, aes(color = "#F31616")) +
  geom_point(data = dplyr::filter(squirrel.data, Ecology == "Terrestrial"),
             size = 2, aes(color = "#F3B116")) +
  geom_line(data = dplyr::filter(predframe.Arb, Ecology == "Arboreal"), color
= "#0EAF28",
            linetype = 1.5) +
  geom_line(data = dplyr::filter(predframe.Fos, Ecology == "Fossorial"), colo
r = "#975822",
            linetype = 1.5) +
  geom_line(data = dplyr::filter(predframe.Gli, Ecology == "Glider"), color =
"#73DAF3",
            linetype = 1.5) +
  geom_line(data = dplyr::filter(predframe.Sca, Ecology == "Scansorial"), col
or = "#F31616",
```

```
             linetype = 1.5) +
  geom_line(data = dplyr::filter(predframe.Ter, Ecology == "Terrestrial"), co
lor = "#F3B116",
             linetype = 1.5) +
  theme_minimal() +
  #theme(legend.position = "top") +
  scale_color_manual(name = "", values = c("#0EAF28","#73DAF3","#975822", "#F
31616", "#F3B116"),labels = c("Arboreal",
             "Glider","Fossorial", "Scansorial", "Terrestrial")) +
  theme(axis.text = element_text(size = 12), axis.title = element_text(size =
12,
             face = "bold")) +
  labs(x = "log(Endocranial volume)", y = "log(Olfactory bulb volume)") +
  geom_text(data = dplyr::filter(squirrel.data, Ecology == "Arboreal"), color
= "#0EAF28",
             aes(label = abbreviation), hjust = -0.3, vjust = 1.1)+
  geom_text(data = dplyr::filter(squirrel.data, Ecology == "Fossorial"), colo
r = "#975822",
             aes(label = abbreviation), hjust = -0.3, vjust = 1.1) +
  geom_text(data = dplyr::filter(squirrel.data, Ecology == "Glider"), color =
"#73DAF3",
             aes(label = abbreviation), hjust = -0.3, vjust = 1.1) +
  geom_text(data = dplyr::filter(squirrel.data, Ecology == "Scansorial"), col
or = "#F31616",
             aes(label = abbreviation), hjust = -0.3, vjust = 1.1) +
  geom_text(data = dplyr::filter(squirrel.data, Ecology == "Terrestrial"), co
lor = "#F3B116",
             aes(label = abbreviation), hjust = -0.3, vjust = 1.1)
```

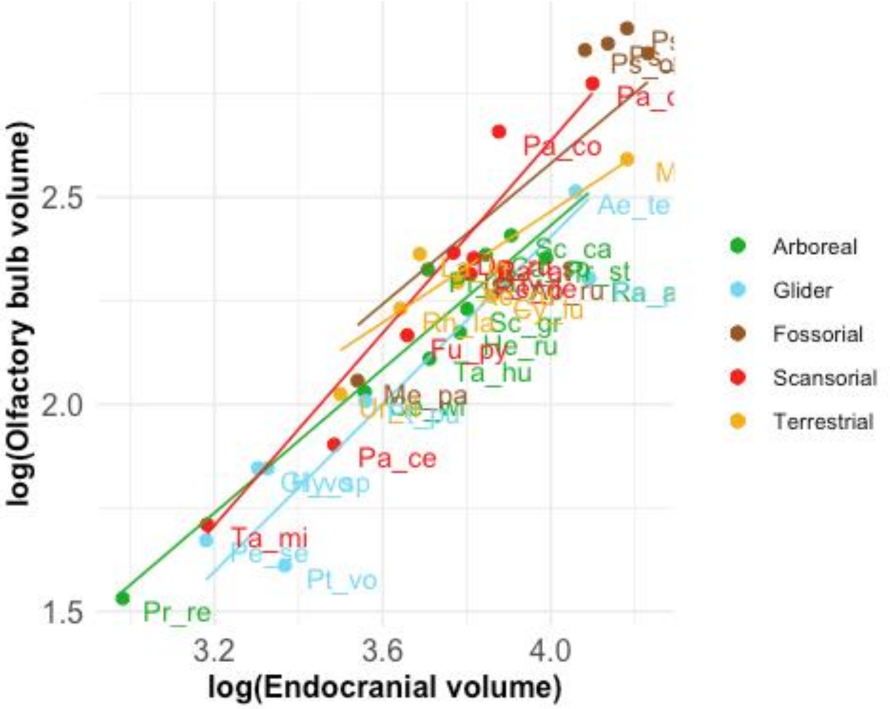

```
#7. PGLS regression for petrosal lobule vs. endocranial volu
me

#Code - Can endocranial volume and locomotion predict petrosal lobule size?

library(phytools) #open tree

## Loading required package: ape

## Loading required package: maps

library(ggplot2) #plots
library(nlme) # GLS analysis
library(RRPP) #pairwise comparisons

#directory
setwd("~/Desktop/Squirrel_June_8_2020/Code")

#Import squirrel data
squirrel.data1<-read.csv("squirrels_PEQ_res.csv", header=T)

#Delete a specific row and taxon in tree
squirrel.data<-squirrel.data1[-c(23),]

#Import tree
tree_squirrel1<-read.newick("Calibrated_tree_meng")

#delete taxa
tree_squirrel<-drop.tip(tree_squirrel1, c("Petinomys_setosus"))

#Transform data to Log10
squirrel.data$Petrosal_lobule_volume_mm3<-log10(squirrel.data$Petrosal_lobule
_volume_mm3)
names(squirrel.data)[names(squirrel.data) == "Petrosal_lobule_volume_mm3"] <-
"PL"

squirrel.data$Brain_volume_mm3<-log10(squirrel.data$Brain_volume_mm3)
names(squirrel.data)[names(squirrel.data) == "Brain_volume_mm3"] <- "Brain.mg
"

#Select other variables
Locomotion<-squirrel.data$Locomotion
abbreviation<-squirrel.data$abbreviation

##########################  Analyses -- OLS ###############################

# Look at the correlation among data point by Family
ggplot(squirrel.data, aes(Brain.mg, PL, color = Family)) +
  theme_light() + theme(legend.position = "top") +
  geom_point(data = dplyr::filter(squirrel.data, Family == "Sciuridae"), shap
```

```
e = 16, size = 3,
             aes(color = "#4DBBD5FF")) +
  geom_point(data = dplyr::filter(squirrel.data, Family == "Aplodontidae"), s
hape = 16, size = 3,
             aes(color = "#E64B35FF")) +
  geom_point(data = dplyr::filter(squirrel.data, Family == "Ischyromyidae"),
shape = 16, size = 3,
             aes(color = "#3C5488FF")) +
  scale_color_manual(name = "", values = c("#3C5488FF","#4DBBD5FF","#E64B35FF
"),labels = c("Ischyromyidae","Sciuridae", "Aplodontidae")) +
  labs(x = "log(Body mass)", y = "log(Petrosal lobule volume)") +
  geom_text(data = dplyr::filter(squirrel.data, Family == "Sciuridae"), color
= "#4DBBD5FF",
            aes(label = abbreviation), hjust = -0.3, vjust = 1.1) +
  geom_text(data = dplyr::filter(squirrel.data, Family == "Ischyromyidae"), c
olor = "#3C5488FF",
            aes(label = abbreviation), hjust = -0.3, vjust = 1.1) +
  geom_text(data = dplyr::filter(squirrel.data, Family == "Aplodontidae"), co
lor = "#E64B35FF",
            aes(label = abbreviation), hjust = -0.3, vjust = 1.1)
```

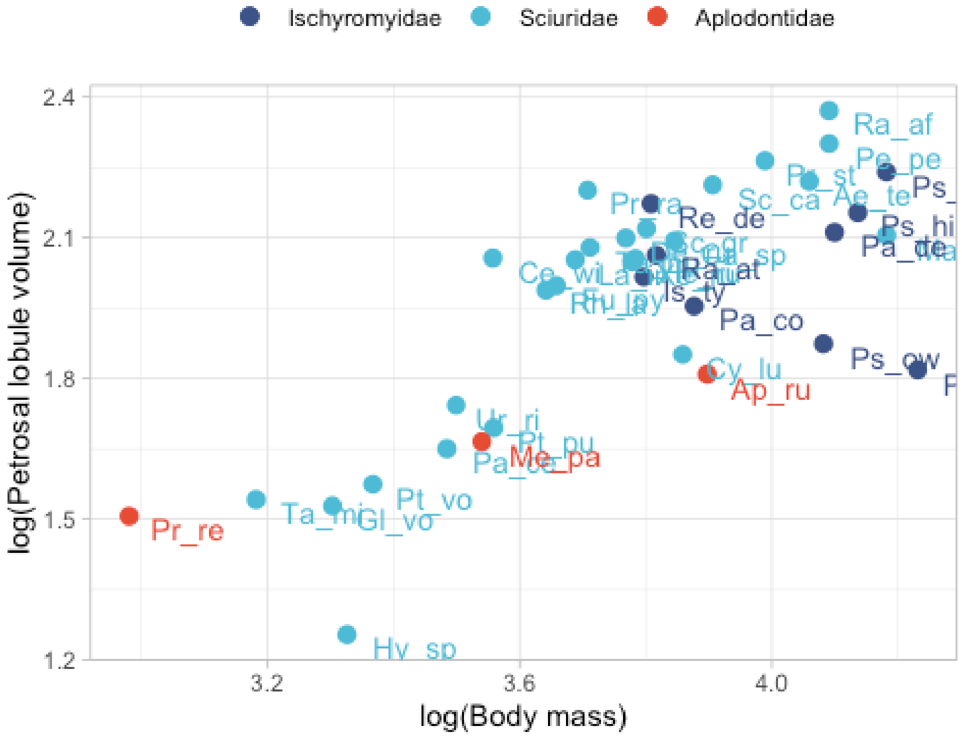


```
#Model using OLS (no phylogeny)
m.ols<-gls(PL ~ Brain.mg + Locomotion, data=squirrel.data, method="ML")
m.ols.body<-gls(PL ~ Brain.mg + Locomotion*Brain.mg, data=squirrel.data, meth
od="ML")
m.ols.loco<-gls(PL ~ Brain.mg*Locomotion, data=squirrel.data, method="ML") #s
ame as above
```

```
#Chosing the model to use
anova(m.ols, m.ols.body, m.ols.loco) # m.ols with lowest AIC

##             Model df       AIC       BIC   logLik   Test  L.Ratio p-value
## m.ols          1  4 -28.27516 -21.83148 18.13758
## m.ols.body     2  5 -27.88083 -19.82624 18.94041 1 vs 2 1.605671  0.2051
## m.ols.loco     3  5 -27.88083 -19.82624 18.94041

summary(m.ols)

## Generalized least squares fit by maximum likelihood
##   Model: PL ~ Brain.mg + Locomotion
##   Data: squirrel.data
##         AIC       BIC   logLik
##   -28.27516 -21.83148 18.13758
##
## Coefficients:
##                  Value Std.Error   t-value p-value
## (Intercept) -0.5604408 0.3276290 -1.710596  0.0963
## Brain.mg     0.7129028 0.0879821  8.102812  0.0000
## Locomotion  -0.0595484 0.0178903 -3.328529  0.0021
##
##  Correlation:
##            (Intr) Brn.mg
## Brain.mg   -0.986
## Locomotion  0.018 -0.166
##
## Standardized residuals:
##        Min         Q1        Med         Q3        Max
## -2.6942650 -0.5886289  0.1440400  0.6237286  1.9018450
##
## Residual standard error: 0.1482073
## Degrees of freedom: 37 total; 34 residual

#Residuals vs fitted plot
plot(fitted(m.ols), residuals(m.ols))
abline(0,0) #pattern visible so not good to use OLS
```

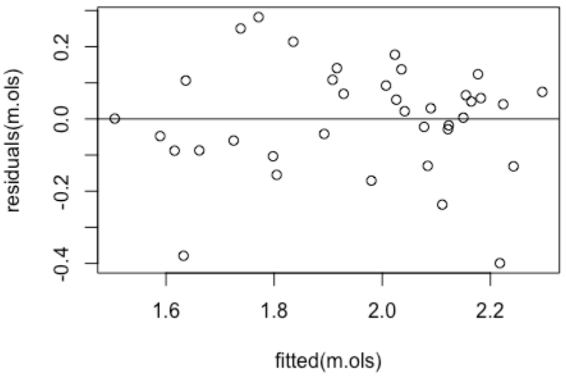


```
# Plot residuals ordered “by phylogeny” (Goldbogen et al., 2019; Supp. p.58)
is_tip <- tree_squirrel$edge[,2]<=length(tree_squirrel$tip.label)
ordered_tips <- tree_squirrel$edge[is_tip,2] # extract the order of tree tips
oj <- residuals(m.ols)
tl <- tree_squirrel$tip.label[ordered_tips] # check order in tree to put them
in same order in csv. file

#Plot residuals against phylogeny
Spe<-squirrel.data$Sp
ggplot(squirrel.data, aes(x=Spe, y = oj, color = Family))+
  theme_light() + theme(legend.position = "top") +
  labs(x = "Species index", y = "OLS residuals") +
  geom_point() +
  geom_abline(intercept = 0, slope = 0) ## pattern, i.e., green group lower c
ompared to blue group
```

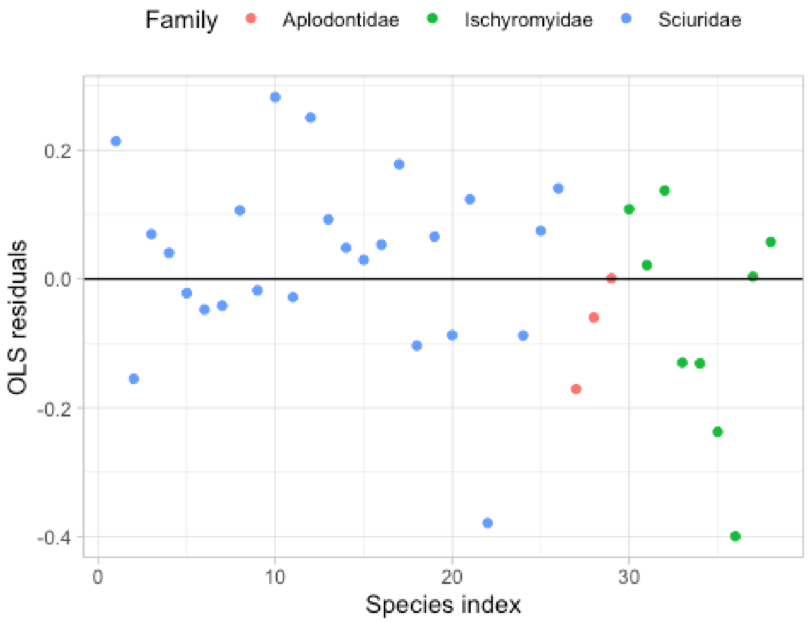

```r
##########################  Analyses -- PGLS ###############################

### Select PGLS model

# Model with locomotion only
Lambda<-gls(PL ~ Brain.mg + Locomotion, data=squirrel.data,
            correlation=corPagel(value=1,phy=tree_squirrel), method="ML")

Brownian<-gls(PL ~ Brain.mg + Locomotion, data=squirrel.data,
              correlation=corBrownian(1,phy=tree_squirrel), method="ML")

OU<-gls(PL ~ Brain.mg + Locomotion, data=squirrel.data,
        correlation=corMartins(1,phy=tree_squirrel,fixed = TRUE), method="ML"
)

Blomberg<-gls(PL ~ Brain.mg + Locomotion, data=squirrel.data,
              correlation=corBlomberg(1.5,phy=tree_squirrel,fixed = TRUE), me
thod="ML") #if 1, this is a Brownian model

# Model with interaction betwenn Locomotion and Body mass
Lambda1<-gls(PL ~ Brain.mg + Locomotion*Brain.mg, data=squirrel.data,
             correlation=corPagel(value=1,phy=tree_squirrel), method="ML")

Brownian1<-gls(PL ~ Brain.mg + Locomotion*Brain.mg, data=squirrel.data,
               correlation=corBrownian(1,phy=tree_squirrel), method="ML")

OU1<-gls(PL ~ Brain.mg + Locomotion*Brain.mg, data=squirrel.data,
         correlation=corMartins(1,phy=tree_squirrel, fixed = TRUE), method="M
L")

Blomberg1<-gls(PL ~ Brain.mg + Locomotion*Brain.mg, data=squirrel.data,
               correlation=corBlomberg(1.5,phy=tree_squirrel,fixed = TRUE), m
ethod="ML") #if 1, this is a Brownian model

#Chosing the best model using AIC
anova(Lambda,Brownian,OU, Blomberg) # Lambda is the best

##            Model df       AIC        BIC    logLik   Test  L.Ratio p-value
## Lambda         1  5 -30.02849 -21.973901 20.014245
## Brownian       2  4 -14.20760  -7.763928 11.103800 1 vs 2 17.82089  <.0001
## OU             3  4 -28.77361 -22.329936 18.386804
## Blomberg       4  4 -11.65012  -5.206452  9.825062

anova(Lambda1,Brownian1,OU1, Blomberg1)

##             Model df       AIC        BIC   logLik   Test  L.Ratio p-value
## Lambda1         1  6 -29.10121 -19.435698 20.55060
## Brownian1       2  5 -15.19369  -7.139097 12.59684 1 vs 2 15.90752   1e-04
## OU1             3  5 -28.94411 -20.889522 19.47206
## Blomberg1       4  5 -12.57676  -4.522172 11.28838

anova(Lambda,Lambda1) #Final model
```

```
##         Model df       AIC       BIC   logLik   Test  L.Ratio p-value
## Lambda      1  5 -30.02849 -21.9739 20.01424
## Lambda1     2  6 -29.10121 -19.4357 20.55060 1 vs 2 1.072715  0.3003

summary(Lambda)

## Generalized least squares fit by maximum likelihood
##   Model: PL ~ Brain.mg + Locomotion
##   Data: squirrel.data
##         AIC       BIC   logLik
##   -30.02849 -21.9739 20.01425
##
## Correlation Structure: corPagel
##  Formula: ~1
##  Parameter estimate(s):
##    lambda
## 0.5440868
##
## Coefficients:
##                  Value Std.Error   t-value p-value
## (Intercept) -1.1148065 0.3344562 -3.333191  0.0021
## Brain.mg     0.8252829 0.0841500  9.807287  0.0000
## Locomotion  -0.0459619 0.0164477 -2.794426  0.0085
##
##  Correlation:
##            (Intr) Brn.mg
## Brain.mg   -0.971
## Locomotion -0.006 -0.132
##
## Standardized residuals:
##         Min          Q1         Med          Q3         Max
## -2.18951779  0.06596869  0.71287507  1.08384885  2.06716356
##
## Residual standard error: 0.171157
## Degrees of freedom: 37 total; 34 residual

#Lambda = 0.54 (PL relative size shows some phylogenetic signal)
#Coefficients: p-value interpretation:
#Endocranial volume and locomotion alone can predict brain size (p-value = 0
and 0.002)

################# post ad-hoc test on locomotion ###############

#tutorial RRPP: https://cran.r-project.org/web/packages/RRPP/vignettes/Using.
RRPP.html

#Import squirrel data - to run the following
squirrel.data1<-read.csv("squirrels_PEQ_res.csv", header=T)
squirrel.data<-squirrel.data1[-c(23),]
tree_squirrel1<-read.newick("Calibrated_tree_meng")
```

```
tree_squirrel<-drop.tip(tree_squirrel1, c("Petinomys_setosus"))

PL<-log10(squirrel.data$Petrosal_lobule_volume_mm3)
Brain<-log10(squirrel.data$Brain_volume_mm3)
Ecology<-squirrel.data$Ecology

#Pairwise comparisons - non-phylogenetic! (Weisbecker et al., 2019; line 552
in Analyses)
interaction_frame <- rrpp.data.frame(pl=PL,brain=Brain,loco=Ecology)
fit<-lm.rrpp(pl~brain+loco,SS.type = c("I"),data=interaction_frame)

summary(fit, formula = FALSE)

##
## Linear Model fit with lm.rrpp
##
## Number of observations: 37
## Number of dependent variables: 1
## Data space dimensions: 1
## Sums of Squares and Cross-products: Type I
## Number of permutations: 1000
##
## Full Model Analysis of Variance
##
##     Df Residual Df       SS Residual SS       Rsq        F Z (from F)  Pr(
>F)
## fit  5          31 1.908127   0.5703861 0.7698677 20.74102   4.322849 0.00
075
##
##
## Redundancy Analysis (PCA on fitted values and residuals)
##
##                 Trace Proportion Rank
## Fitted    0.05300354  0.7698677    1
## Residuals 0.01584406  0.2301324    1
## Total     0.06884760  1.0000001    1
##
## Eigenvalues
##
##                  PC1
## Fitted    0.05300354
## Residuals 0.01584406
## Total     0.06884760

anova(fit)

##
## Analysis of Variance, using Residual Randomization
## Permutation procedure: Randomization of null model residuals
## Number of permutations: 1000
## Estimation method: Ordinary Least Squares
```

```
## Sums of Squares and Cross-products: Type I
## Effect sizes (Z) based on F distributions
##
##           Df      SS      MS     Rsq      F      Z Pr(>F)
## brain      1 1.40096 1.40096 0.56524 76.141 2.4119  0.001 **
## loco       4 0.50716 0.12679 0.20462  6.891 2.5979  0.001 **
## Residuals 31 0.57039 0.01840 0.23013
## Total     36 2.47851
## ---
## Signif. codes:  0 '***' 0.001 '**' 0.01 '*' 0.05 '.' 0.1 ' ' 1
##
## Call: lm.rrpp(f1 = pl ~ brain + loco, SS.type = c("I"), data = interaction
_frame)
```

```
Interactions <- pairwise(fit,covariate=interaction_frame$body,
                         groups=interaction_frame$loco)

summary(Interactions, test.type="dist") # shows which locomotor modes are sig
nificantly different
```

```
##
## Pairwise comparisons
##
## Groups: Arboreal Fossorial Glider Scansorial Terrestrial
##
## RRPP: 1000 permutations
##
## LS means:
## Vectors hidden (use show.vectors = TRUE to view)
##
## Pairwise distances between means, plus statistics
##                                 d UCL (95%)           Z Pr > d
## Arboreal:Fossorial     0.33713459 0.1761594  4.76341466  0.001
## Arboreal:Glider        0.24506289 0.1823458  3.05203018  0.008
## Arboreal:Scansorial    0.12822301 0.1621418  1.20631417  0.131
## Arboreal:Terrestrial   0.15872202 0.1792571  1.62851568  0.086
## Fossorial:Glider       0.09207170 0.2118122  0.09484013  0.401
## Fossorial:Scansorial   0.20891158 0.1831238  2.29501883  0.027
## Fossorial:Terrestrial  0.17841258 0.1906097  1.70935688  0.068
## Glider:Scansorial      0.11683988 0.1838993  0.73298353  0.224
## Glider:Terrestrial     0.08634087 0.1929839  0.09243771  0.420
## Scansorial:Terrestrial 0.03049900 0.1781101 -0.79290008  0.751
```

```
# Based on the fit model, how does brain size predicted to vary for each loco
motor category? (what I understand this does)
sizeDF <- data.frame(loco = c("Arboreal", "Scansorial","Glider","Terrestrial"
,"Fossorial"))
rownames(sizeDF) <- c("Arboreal", "Scansorial","Glider","Terrestrial","Fossor
ial")
sizePreds <- predict(fit,sizeDF)
```

```
##
## Warning: Not all variables in model accounted for in newdata.
## Missing variables will be averaged from observed data for prediction.
```

```
plot(sizePreds) # The brain size of Arboreal and Glider are more similar than
with the other locomotor categories
```

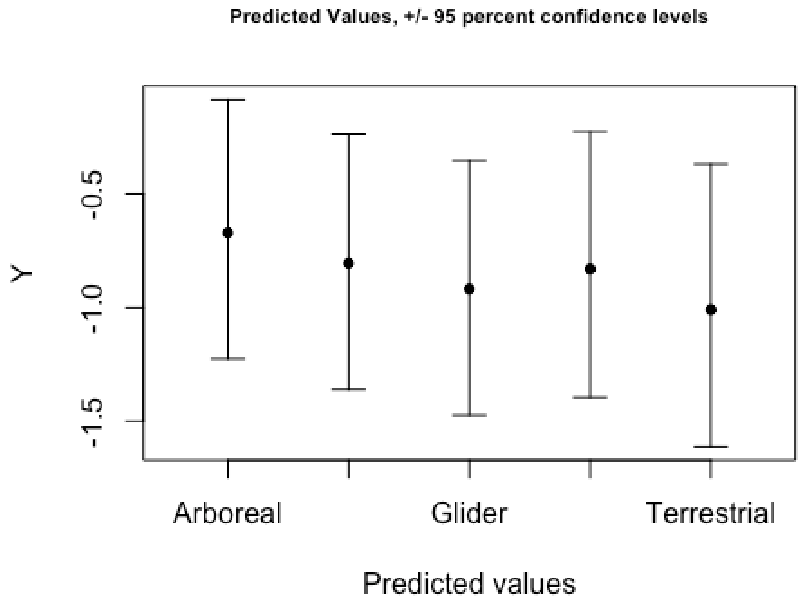


```
################ Make graph with PGLS regression lines for each locomotion ##
#########

#Import data
squirrel.data1<-read.csv("squirrels_PEQ_res.csv", header=T)
squirrel.data<-squirrel.data1[-c(23),]

#Import tree
tree_squirrel1<-read.newick("Calibrated_tree_meng")
tree_squirrel<-drop.tip(tree_squirrel1, c("Petinomys_setosus"))

#Transform data to log10
squirrel.data$Petrosal_lobule_volume_mm3<-log10(squirrel.data$Petrosal_lobule
_volume_mm3)
names(squirrel.data)[names(squirrel.data) == "Petrosal_lobule_volume_mm3"] <-
"PL"
squirrel.data$Brain_volume_mm3<-log10(squirrel.data$Brain_volume_mm3)
names(squirrel.data)[names(squirrel.data) == "Brain_volume_mm3"] <- "Brain.mg
"

#subset dataset per locomotion to use in model
Arb<- squirrel.data[ which(squirrel.data$Ecology=='Arboreal'), ]
```

```
Fos<- squirrel.data[ which(squirrel.data$Ecology=='Fossorial'), ]
Gli<- squirrel.data[ which(squirrel.data$Ecology=='Glider'), ]
Sca<- squirrel.data[ which(squirrel.data$Ecology=='Scansorial'), ]
Ter<- squirrel.data[ which(squirrel.data$Ecology=='Terrestrial'), ]

#Find taxa with specific locomotion
Arb_sp<-subset(squirrel.data, Ecology=="Arboreal", select=Species)
Fos_sp<-subset(squirrel.data, Ecology=="Fossorial", select=Species)
Gli_sp<-subset(squirrel.data, Ecology=="Glider", select=Species)
Sca_sp<-subset(squirrel.data, Ecology=="Scansorial", select=Species)
Ter_sp<-subset(squirrel.data, Ecology=="Terrestrial", select=Species)

#Select taxa per locomotion for the tree
Arb_species<-c("Protoxerus_stangeri","Heliosciurus_rufobrachium","Callosciuru
s_sp",
               "Sciurus_carolinensis","Sciurus_granatensis","Tamiasciurus_hud
sonicus",
               "Protosciurus_rachelae","Ratufa_affinis","Cedromus_wilsoni",
               "Prosciurus_relictus")

Fos_species<-c("Aplodontia_rufa","Mesogaulus_paniensis","Ischyromys_typus",
               "Pseudotomus_oweni","Pseudotomus_petersoni","Pseudotomus_hians
",
               "Pseudotomus_horribilis")

Gli_species<-c("Pteromyscus_pulverulentus","Aeromys_tephromelas","Pteromys_vo
lans",
               "Petaurista_petaurista","Hylopetes_spadiceus",
               "Glaucomys_volans")

Sca_species<-c("Paraxerus_cepapi","Funisciurus_pyrropus","Tamias_minimus",
               "Dremomys_rufigenis","Rapamys_atramontis","Reithroparamys_deli
catissimus",
               "Paramys_copei","Paramys_delicatus")

Ter_species<-c("Xerus_rutilus","Cynomys_ludovicianus","Urocitellus_richardson
ii",
               "Marmota_marmota","Lariscus_insignis","Rhinosciurus_laticaudat
us")

#subset tree per locomotion
Arb_Tree<-drop.tip(tree_squirrel,tree_squirrel$tip.label[-match(Arb_species,
tree_squirrel$tip.label)])
Fos_Tree<-drop.tip(tree_squirrel,tree_squirrel$tip.label[-match(Fos_species,
tree_squirrel$tip.label)])
Gli_Tree<-drop.tip(tree_squirrel,tree_squirrel$tip.label[-match(Gli_species,
tree_squirrel$tip.label)])
Sca_Tree<-drop.tip(tree_squirrel,tree_squirrel$tip.label[-match(Sca_species,
tree_squirrel$tip.label)])
Ter_Tree<-drop.tip(tree_squirrel,tree_squirrel$tip.label[-match(Ter_species,
```

```
tree_squirrel$tip.label)])

#Create model PGLS regression line for each locomotion
Arbline_Br_B <-gls(PL ~ Brain.mg, correlation=corPagel (1,phy=Arb_Tree,fixed=
T), data=Arb)

Fosline_Br_B <-gls(PL ~ Brain.mg, correlation=corPagel (1,phy=Fos_Tree), data
=Fos)

Gliline_Br_B <-gls(PL ~ Brain.mg, correlation=corPagel (1,phy=Gli_Tree), data
=Gli)

Scaline_Br_B <-gls(PL ~ Brain.mg, correlation=corPagel (1,phy=Sca_Tree), data
=Sca)

Terline_Br_B <-gls(PL ~ Brain.mg, correlation=corPagel (1,phy=Ter_Tree), data
=Ter)

#Prepare PGLS for each locomotor mode
pgls.fit.Arb <- predict(Arbline_Br_B)
predframe.Arb <- with(Arb, data.frame(Species, Ecology, Brain.mg, PL = pgls.f
it.Arb))

pgls.fit.Fos <- predict(Fosline_Br_B)
predframe.Fos <- with(Fos, data.frame(Species, Ecology, Brain.mg, PL = pgls.f
it.Fos))

pgls.fit.Gli <- predict(Gliline_Br_B)
predframe.Gli <- with(Gli, data.frame(Species, Ecology, Brain.mg, PL = pgls.f
it.Gli))

pgls.fit.Sca <- predict(Scaline_Br_B)
predframe.Sca <- with(Sca, data.frame(Species, Ecology, Brain.mg, PL = pgls.f
it.Sca))

pgls.fit.Ter <- predict(Terline_Br_B)
predframe.Ter <- with(Ter, data.frame(Species, Ecology, Brain.mg, PL = pgls.f
it.Ter))

#Make graph with PGLS corrected regressions
ggplot(squirrel.data, aes(Brain.mg, PL, color = Ecology)) +
  geom_point(data = dplyr::filter(squirrel.data, Ecology == "Arboreal"),
             size = 2, aes(color = "#0EAF28")) +
  geom_point(data = dplyr::filter(squirrel.data, Ecology == "Fossorial"),
             size = 2, aes(color = "#975822")) +
  geom_point(data = dplyr::filter(squirrel.data, Ecology == "Glider"),
             size = 2, aes(color = "#73DAF3")) +
  geom_point(data = dplyr::filter(squirrel.data, Ecology == "Scansorial"),
             size = 2, aes(color = "#F31616")) +
  geom_point(data = dplyr::filter(squirrel.data, Ecology == "Terrestrial"),
             size = 2, aes(color = "#F3B116")) +
```

```
  geom_line(data = dplyr::filter(predframe.Arb, Ecology == "Arboreal"), color
= "#0EAF28",
            linetype = 1.5) +
  geom_line(data = dplyr::filter(predframe.Fos, Ecology == "Fossorial"), colo
r = "#975822",
            linetype = 1.5) +
  geom_line(data = dplyr::filter(predframe.Gli, Ecology == "Glider"), color =
"#73DAF3",
            linetype = 1.5) +
  geom_line(data = dplyr::filter(predframe.Sca, Ecology == "Scansorial"), col
or = "#F31616",
            linetype = 1.5) +
  geom_line(data = dplyr::filter(predframe.Ter, Ecology == "Terrestrial"), co
lor = "#F3B116",
            linetype = 1.5) +
  theme_minimal() +
  #theme(legend.position = "top") +
  scale_color_manual(name = "", values = c("#0EAF28","#73DAF3","#975822", "#F
31616", "#F3B116"),labels = c("Arboreal",
            "Glider","Fossorial", "Scansorial", "Terrestrial")) +
  theme(axis.text = element_text(size = 12), axis.title = element_text(size =
12,
              face = "bold")) +
  labs(x = "log(Endocranial volume)", y = "log(Petrosal lobule volume)") +
  geom_text(data = dplyr::filter(squirrel.data, Ecology == "Arboreal"), color
= "#0EAF28",
            aes(label = abbreviation), hjust = -0.3, vjust = 1.1)+
  geom_text(data = dplyr::filter(squirrel.data, Ecology == "Fossorial"), colo
r = "#975822",
            aes(label = abbreviation), hjust = -0.3, vjust = 1.1) +
  geom_text(data = dplyr::filter(squirrel.data, Ecology == "Glider"), color =
"#73DAF3",
            aes(label = abbreviation), hjust = -0.3, vjust = 1.1) +
  geom_text(data = dplyr::filter(squirrel.data, Ecology == "Scansorial"), col
or = "#F31616",
            aes(label = abbreviation), hjust = -0.3, vjust = 1.1) +
  geom_text(data = dplyr::filter(squirrel.data, Ecology == "Terrestrial"), co
lor = "#F3B116",
            aes(label = abbreviation), hjust = -0.3, vjust = 1.1)
```

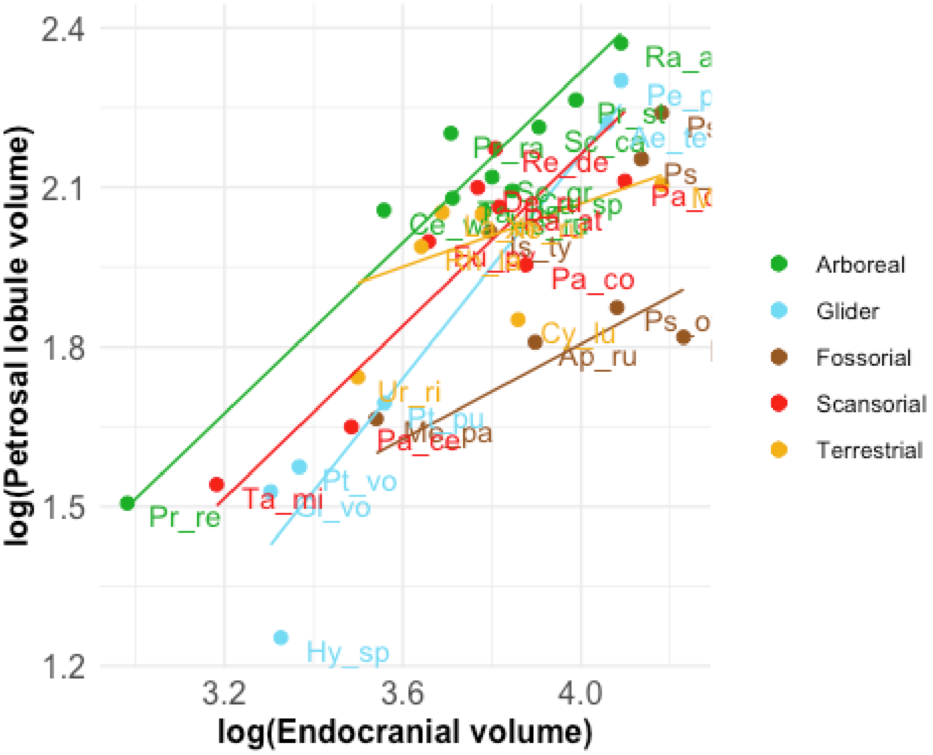
2.4
2.1
1.8
1.5
1.2
3.2
3.6
4.0
log(Petrosal lobule volume)
log(Endocranial volume)
Arboreal
Glider
Fossorial
Scansorial
Terrestrial
Pr_re
Ta_mi
Pt_vo
Hy_sp
Ur_ri
Pt_pu
Me_pa
Cy_lu
Ap_ru
Pa_co
Re_de
Is_ty

```
# 8. PGLS regression for neocortical surface area vs. endocr
anial surface area

#Can endocranial volume and locomotion predict neocortical size?

library(phytools) #open tree

## Loading required package: ape

## Loading required package: maps

library(ggplot2) #plots
library(nlme) # GLS analysis
library(RRPP) #pairwise comparisons



#directory
setwd("~/Desktop/Squirrel_June_8_2020/Code")

#Import squirrel data
squirrel.data<-read.csv("squirrels_PEQ_res.csv", header=T)

#Import tree
tree_squirrel<-read.newick("Calibrated_tree_meng")

#Transform data to log10
squirrel.data$Brain_surface_mm2<-log10(squirrel.data$Brain_surface_mm2)
names(squirrel.data)[names(squirrel.data) == "Brain_surface_mm2"] <- "Brain.s
urf"

squirrel.data$Neocortex_surface_mm2<-log10(squirrel.data$Neocortex_surface_mm
2)
names(squirrel.data)[names(squirrel.data) == "Neocortex_surface_mm2"] <- "Neo
cortex"

#Select other variables
Locomotion<-squirrel.data$Locomotion
abbreviation<-squirrel.data$abbreviation

##########################  Analyses -- OLS ###############################

# Look at the correlation among data point by Family
ggplot(squirrel.data, aes(Brain.surf, Neocortex, color = Family)) +
  theme_light() + theme(legend.position = "top") +
  geom_point(data = dplyr::filter(squirrel.data, Family == "Sciuridae"), shap
e = 16, size = 3,
             aes(color = "#4DBBD5FF")) +
  geom_point(data = dplyr::filter(squirrel.data, Family == "Aplodontidae"), s
hape = 16, size = 3,
             aes(color = "#E64B35FF")) +
```

```
  geom_point(data = dplyr::filter(squirrel.data, Family == "Ischyromyidae"), 
shape = 16, size = 3,
             aes(color = "#3C5488FF")) +
  scale_color_manual(name = "", values = c("#3C5488FF","#4DBBD5FF","#E64B35FF
"),labels = c("Ischyromyidae","Sciuridae", "Aplodontidae")) +
  labs(x = "log(Body mass)", y = "log(Endocranial volume)") +
  geom_text(data = dplyr::filter(squirrel.data, Family == "Sciuridae"), color
= "#4DBBD5FF",
            aes(label = abbreviation), hjust = -0.3, vjust = 1.1) +
  geom_text(data = dplyr::filter(squirrel.data, Family == "Ischyromyidae"), c
olor = "#3C5488FF",
            aes(label = abbreviation), hjust = -0.3, vjust = 1.1) +
  geom_text(data = dplyr::filter(squirrel.data, Family == "Aplodontidae"), co
lor = "#E64B35FF",
            aes(label = abbreviation), hjust = -0.3, vjust = 1.1)
```

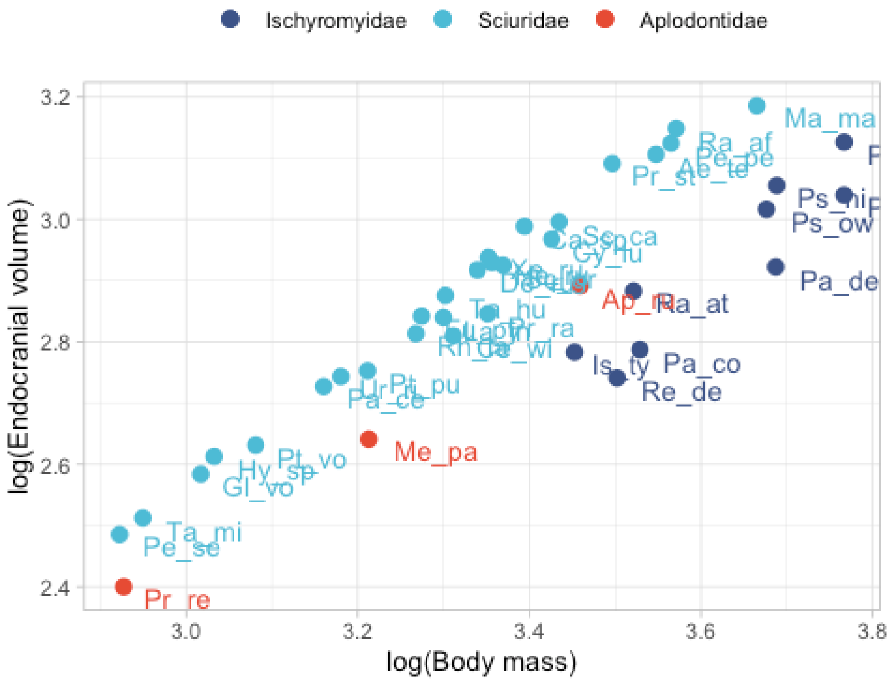


```
#Model using OLS (no phylogeny)
m.ols<-gls(Neocortex ~ Brain.surf + Locomotion, data=squirrel.data, method="M
L")
m.ols.body<-gls(Neocortex ~ Brain.surf + Locomotion*Brain.surf, data=squirrel
.data, method="ML")
m.ols.loco<-gls(Neocortex ~ Brain.surf*Locomotion, data=squirrel.data, method
="ML") #same as above

#Chosing the model to use
anova(m.ols, m.ols.body, m.ols.loco) # m.ols.body (and m.ols.loco) with lowes
t AIC
```

```
##            Model df       AIC       BIC   logLik   Test   L.Ratio p-value
## m.ols          1  4 -64.65549 -58.10514 36.32774
## m.ols.body     2  5 -63.40776 -55.21983 36.70388 1 vs 2 0.7522739  0.3858
## m.ols.loco     3  5 -63.40776 -55.21983 36.70388

summary(m.ols)

## Generalized least squares fit by maximum likelihood
##   Model: Neocortex ~ Brain.surf + Locomotion
##   Data: squirrel.data
##         AIC       BIC   logLik
##   -64.65549 -58.10514 36.32774
##
## Coefficients:
##                   Value  Std.Error   t-value p-value
## (Intercept)  0.3828315 0.23324923  1.641298  0.1097
## Brain.surf   0.7389898 0.06975066 10.594735  0.0000
## Locomotion  -0.0048370 0.01116764 -0.433130  0.6676
##
##  Correlation:
##            (Intr) Brn.sr
## Brain.surf -0.989
## Locomotion  0.010 -0.142
##
## Standardized residuals:
##         Min          Q1         Med          Q3         Max
## -2.36772038 -0.45361319  0.07752601  0.80781261  1.41391142
##
## Residual standard error: 0.09302083
## Degrees of freedom: 38 total; 35 residual

#Residuals vs fitted plot
plot(fitted(m.ols), residuals(m.ols))
abline(0,0) #pattern visible so not good to use OLS
```

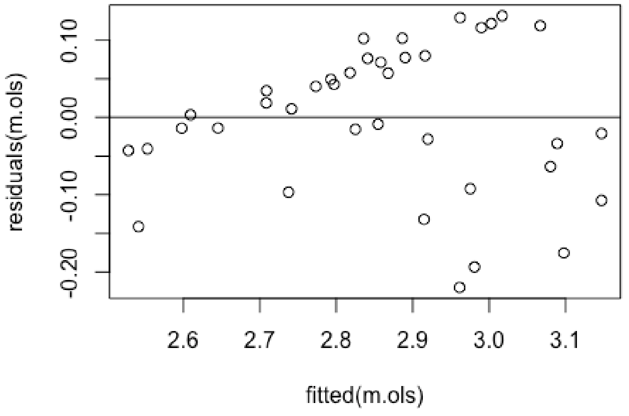

```
# Plot residuals ordered “by phylogeny” (Goldbogen et al., 2019; Supp. p.58)
is_tip <- tree_squirrel$edge[,2]<=length(tree_squirrel$tip.label)
ordered_tips <- tree_squirrel$edge[is_tip,2] # extract the order of tree tips
oj <- residuals(m.ols)
tl <- tree_squirrel$tip.label[ordered_tips] # check order in tree to put them
in same order in csv. file

#Plot residuals against phylogeny
Spe<-squirrel.data$Sp
ggplot(squirrel.data, aes(x=Spe, y = oj, color = Family))+
  theme_light() + theme(legend.position = "top") +
  labs(x = "Species index", y = "OLS residuals") +
  geom_point() +
  geom_abline(intercept = 0, slope = 0) ## pattern, i.e., red ones are very l
ow so need PGLS
```

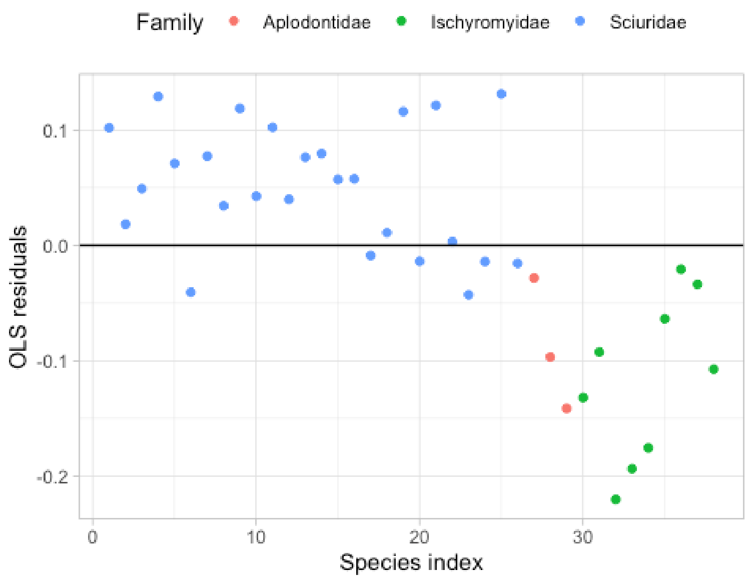


```
##########################  Analyses -- PGLS ###############################

### Select PGLS model
# Model with locomotion only
Lambda<-gls(Neocortex ~ Brain.surf + Locomotion, data=squirrel.data,
            correlation=corPagel(value=1,phy=tree_squirrel), method="ML")

Brownian<-gls(Neocortex ~ Brain.surf + Locomotion, data=squirrel.data,
              correlation=corBrownian(1,phy=tree_squirrel), method="ML")

OU<-gls(Neocortex ~ Brain.surf + Locomotion, data=squirrel.data,
        correlation=corMartins(1,phy=tree_squirrel,fixed = TRUE), method="ML"
)
```

```
Blomberg<-gls(Neocortex ~ Brain.surf + Locomotion, data=squirrel.data,
              correlation=corBlomberg(1.5,phy=tree_squirrel,fixed = TRUE), me
thod="ML") #if 1, this is a Brownian model

# Model with interaction betwenn Locomotion and Body mass
Lambda1<-gls(Neocortex ~ Brain.surf + Locomotion*Brain.surf, data=squirrel.da
ta,
             correlation=corPagel(value=1,phy=tree_squirrel), method="ML")

Brownian1<-gls(Neocortex ~ Brain.surf + Locomotion*Brain.surf, data=squirrel.
data,
               correlation=corBrownian(1,phy=tree_squirrel), method="ML")

OU1<-gls(Neocortex ~ Brain.surf + Locomotion*Brain.surf, data=squirrel.data,
         correlation=corMartins(1,phy=tree_squirrel, fixed = TRUE), method="M
L")

Blomberg1<-gls(Neocortex ~ Brain.surf + Locomotion*Brain.surf, data=squirrel.
data,
               correlation=corBlomberg(1.5,phy=tree_squirrel,fixed = TRUE), m
ethod="ML") #if 1, this is a Brownian model

#Chosing the best model using AIC
anova(Lambda,Brownian,OU, Blomberg) # Lambda is the best

##            Model df        AIC       BIC   logLik   Test   L.Ratio p-value
## Lambda         1  5 -119.09879 -110.9109 64.54940
## Brownian       2  4 -120.95371 -114.4034 64.47685 1 vs 2 0.1450887  0.7033
## OU             3  4  -69.24125  -62.6909 38.62062
## Blomberg       4  4 -120.74992 -114.1996 64.37496

anova(Lambda1,Brownian1,OU1, Blomberg1) # Lambda1 is the best

##             Model df        AIC        BIC   logLik   Test   L.Ratio p-value
## Lambda1         1  6 -118.23893 -108.41342 65.11947
## Brownian1       2  5 -119.77258 -111.58465 64.88629 1 vs 2 0.4663541  0.4947
## OU1             3  5  -67.53575  -59.34782 38.76788
## Blomberg1       4  5 -119.63418 -111.44625 64.81709

anova(Brownian,Blomberg) # Lambda1 is the best model overall

##            Model df       AIC       BIC   logLik
## Brownian       1  4 -120.9537 -114.4034 64.47685
## Blomberg       2  4 -120.7499 -114.1996 64.37496

summary(Brownian) #Final model

## Generalized least squares fit by maximum likelihood
##   Model: Neocortex ~ Brain.surf + Locomotion
##   Data: squirrel.data
##         AIC       BIC   logLik
##   -120.9537 -114.4034 64.47685
```

```
##
## Correlation Structure: corBrownian
##  Formula: ~1
##  Parameter estimate(s):
## numeric(0)
##
## Coefficients:
##                  Value  Std.Error  t-value p-value
## (Intercept) -0.5025502 0.10359552 -4.85108   0e+00
## Brain.surf   0.9759327 0.02446356 39.89331   0e+00
## Locomotion  -0.0138061 0.00362158 -3.81218   5e-04
##
##  Correlation:
##            (Intr) Brn.sr
## Brain.surf -0.832
## Locomotion -0.004 -0.117
##
## Standardized residuals:
##        Min         Q1        Med         Q3        Max
## -1.1321873  0.4659607  1.3042666  1.4529305  1.8358469
##
## Residual standard error: 0.1296314
## Degrees of freedom: 38 total; 35 residual
```

```
summary(Lambda)
```

```
## Generalized least squares fit by maximum likelihood
##   Model: Neocortex ~ Brain.surf + Locomotion
##   Data: squirrel.data
##         AIC       BIC  logLik
##   -119.0988 -110.9109 64.5494
##
## Correlation Structure: corPagel
##  Formula: ~1
##  Parameter estimate(s):
##   lambda
## 1.007677
##
## Coefficients:
##                  Value  Std.Error  t-value p-value
## (Intercept) -0.5193561 0.10200079 -5.09169   0e+00
## Brain.surf   0.9807544 0.02262787 43.34276   0e+00
## Locomotion  -0.0141460 0.00339692 -4.16436   2e-04
##
##  Correlation:
##            (Intr) Brn.sr
## Brain.surf -0.785
## Locomotion -0.026 -0.092
##
## Standardized residuals:
```

```
##        Min         Q1        Med         Q3        Max
## -1.0216783  0.4427651  1.1959113  1.3241735  1.6798138
##
## Residual standard error: 0.1430669
## Degrees of freedom: 38 total; 35 residual

#Lambda = 1.01 (Neocortical size shows a lot of phylogenetic signal)
#Coefficients: p-value interpretation:
#Locomotion and endocranial surface both alone predict brain size (p-value =
0)

################# post ad-hoc test on locomotion ################

#tutorial RRPP: https://cran.r-project.org/web/packages/RRPP/vignettes/Using.
RRPP.html

#Import squirrel data - to run the following
squirrel.data<-read.csv("squirrels_PEQ_res.csv", header=T)
Neocortex<-log10(squirrel.data$Neocortex_surface_mm2)
Brains<-log10(squirrel.data$Brain_surface_mm2)
Ecology<-squirrel.data$Ecology

#Pairwise comparisons - non-phylogenetic! (Weisbecker et al., 2019; line 552
in Analyses)
interaction_frame <- rrpp.data.frame(neocortex=Neocortex,brains=Brains,loco=E
cology)
fit<-lm.rrpp(neocortex~brains+loco,SS.type = c("I"),data=interaction_frame)

summary(fit, formula = FALSE)

##
## Linear Model fit with lm.rrpp
##
## Number of observations: 38
## Number of dependent variables: 1
## Data space dimensions: 1
## Sums of Squares and Cross-products: Type I
## Number of permutations: 1000
##
## Full Model Analysis of Variance
##
##     Df Residual Df    SS Residual SS       Rsq        F Z (from F)  Pr(>F)
## fit  5          32 1.209   0.1852462 0.8671352 41.76928   5.049993 0.00075
##
##
## Redundancy Analysis (PCA on fitted values and residuals)
##
##                 Trace Proportion Rank
## Fitted    0.03267568  0.8671352    1
## Residuals 0.00500665  0.1328646    1
```

```
## Total     0.03768233  0.9999999    1
##
## Eigenvalues
##
##                   PC1
## Fitted    0.03267568
## Residuals 0.00500665
## Total     0.03768233
```

```
anova(fit) # When phylogeny is NOT taken into account, all predict brain size
```

```
##
## Analysis of Variance, using Residual Randomization
## Permutation procedure: Randomization of null model residuals
## Number of permutations: 1000
## Estimation method: Ordinary Least Squares
## Sums of Squares and Cross-products: Type I
## Effect sizes (Z) based on F distributions
##
##           Df      SS      MS     Rsq       F      Z Pr(>F)
## brains     1 1.06367 1.06367 0.76290 183.742 2.8412  0.001 **
## loco       4 0.14533 0.03633 0.10423   6.276 2.4975  0.004 **
## Residuals 32 0.18525 0.00579 0.13286
## Total     37 1.39425
## ---
## Signif. codes:  0 '***' 0.001 '**' 0.01 '*' 0.05 '.' 0.1 ' ' 1
##
## Call: lm.rrpp(f1 = neocortex ~ brains + loco, SS.type = c("I"), data = int
eraction_frame)
```

```
Interactions <- pairwise(fit,covariate=interaction_frame$body,
                         groups=interaction_frame$loco)

summary(Interactions, test.type="dist") # shows which locomotor modes are sig
nificantly different
```

```
##
## Pairwise comparisons
##
## Groups: Arboreal Fossorial Glider Scansorial Terrestrial
##
## RRPP: 1000 permutations
##
## LS means:
## Vectors hidden (use show.vectors = TRUE to view)
##
## Pairwise distances between means, plus statistics
##                                 d  UCL (95%)          Z Pr > d
## Arboreal:Fossorial     0.151245875 0.09811670  3.5838130  0.003
## Arboreal:Glider        0.015201705 0.09308742 -0.8059797  0.749
## Arboreal:Scansorial    0.125299678 0.09058343  3.2772866  0.005
```

```
## Arboreal:Terrestrial   0.002237616 0.09277936 -1.2923687  0.967
## Fossorial:Glider       0.136044170 0.11555938  2.5301447  0.022
## Fossorial:Scansorial   0.025946197 0.09694076 -0.5314904  0.635
## Fossorial:Terrestrial  0.153483491 0.10676486  3.3712595  0.003
## Glider:Scansorial      0.110097973 0.09387547  2.4515613  0.019
## Glider:Terrestrial     0.017439321 0.10175309 -0.8512378  0.766
## Scansorial:Terrestrial 0.127537294 0.09998827  2.7788212  0.010

# Based on the fit model, how does brain size predicted to vary for each loco
motor category? (what I understand this does)
sizeDF <- data.frame(loco = c("Arboreal", "Scansorial","Glider","Terrestrial"
,"Fossorial"))
rownames(sizeDF) <- c("Arboreal", "Scansorial","Glider","Terrestrial","Fossor
ial")
sizePreds <- predict(fit,sizeDF)

##
## Warning: Not all variables in model accounted for in newdata.
## Missing variables will be averaged from observed data for prediction.

plot(sizePreds) # The brain size of Arboreal and Glider are more similar than
with the other locomotor categories
```

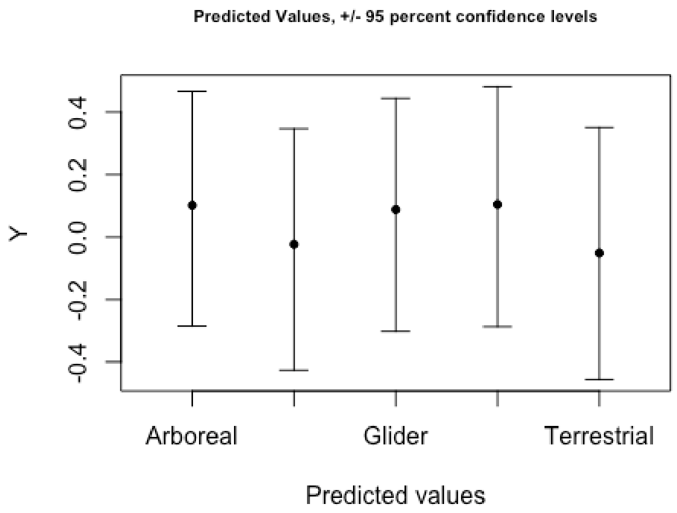


```
################ Make graph with PGLS regression lines for each locomotion ##
#########

#Import squirrel data
squirrel.data<-read.csv("squirrels_PEQ_res.csv", header=T)

squirrel.data$Brain_surface_mm2<-log10(squirrel.data$Brain_surface_mm2)
names(squirrel.data)[names(squirrel.data) == "Brain_surface_mm2"] <- "Brain.s
urf"
squirrel.data$Neocortex_surface_mm2<-log10(squirrel.data$Neocortex_surface_mm
```

```
2)
names(squirrel.data)[names(squirrel.data) == "Neocortex_surface_mm2"] <- "Neo
cortex"

#subset dataset per locomotion to use in model
Arb<- squirrel.data[ which(squirrel.data$Ecology=='Arboreal'), ]
Fos<- squirrel.data[ which(squirrel.data$Ecology=='Fossorial'), ]
Gli<- squirrel.data[ which(squirrel.data$Ecology=='Glider'), ]
Sca<- squirrel.data[ which(squirrel.data$Ecology=='Scansorial'), ]
Ter<- squirrel.data[ which(squirrel.data$Ecology=='Terrestrial'), ]

#Find taxa with specific locomotion
Arb_sp<-subset(squirrel.data, Ecology=="Arboreal", select=Species)
Fos_sp<-subset(squirrel.data, Ecology=="Fossorial", select=Species)
Gli_sp<-subset(squirrel.data, Ecology=="Glider", select=Species)
Sca_sp<-subset(squirrel.data, Ecology=="Scansorial", select=Species)
Ter_sp<-subset(squirrel.data, Ecology=="Terrestrial", select=Species)

#Select taxa per locomotion for the tree
Arb_species<-c("Protoxerus_stangeri","Heliosciurus_rufobrachium","Callosciuru
s_sp",
               "Sciurus_carolinensis","Sciurus_granatensis","Tamiasciurus_hud
sonicus",
               "Protosciurus_rachelae","Ratufa_affinis","Cedromus_wilsoni",
               "Prosciurus_relictus")

Fos_species<-c("Aplodontia_rufa","Mesogaulus_paniensis","Ischyromys_typus",
               "Pseudotomus_oweni","Pseudotomus_petersoni","Pseudotomus_hians
",
               "Pseudotomus_horribilis")

Gli_species<-c("Pteromyscus_pulverulentus","Aeromys_tephromelas","Pteromys_vo
lans",
               "Petaurista_petaurista","Hylopetes_spadiceus","Petinomys_setos
us",
               "Glaucomys_volans")

Sca_species<-c("Paraxerus_cepapi","Funisciurus_pyrropus","Tamias_minimus",
               "Dremomys_rufigenis","Rapamys_atramontis","Reithroparamys_deli
catissimus",
               "Paramys_copei","Paramys_delicatus")

Ter_species<-c("Xerus_rutilus","Cynomys_ludovicianus","Urocitellus_richardson
ii",
               "Marmota_marmota","Lariscus_insignis","Rhinosciurus_laticaudat
us")

#subset tree per locomotion
Arb_Tree<-drop.tip(tree_squirrel,tree_squirrel$tip.label[-match(Arb_species,
tree_squirrel$tip.label)])
```

```
Fos_Tree<-drop.tip(tree_squirrel,tree_squirrel$tip.label[-match(Fos_species,
tree_squirrel$tip.label)])
Gli_Tree<-drop.tip(tree_squirrel,tree_squirrel$tip.label[-match(Gli_species,
tree_squirrel$tip.label)])
Sca_Tree<-drop.tip(tree_squirrel,tree_squirrel$tip.label[-match(Sca_species,
tree_squirrel$tip.label)])
Ter_Tree<-drop.tip(tree_squirrel,tree_squirrel$tip.label[-match(Ter_species,
tree_squirrel$tip.label)])

#Create model PGLS regression line for each locomotion
Arbline_Br_B <-gls(Neocortex ~ Brain.surf, correlation=corBrownian (1,phy=Arb
_Tree), data=Arb)

Fosline_Br_B <-gls(Neocortex ~ Brain.surf, correlation=corBrownian (1,phy=Fos
_Tree), data=Fos)

Gliline_Br_B <-gls(Neocortex ~ Brain.surf, correlation=corBrownian (1,phy=Gli
_Tree), data=Gli)

Scaline_Br_B <-gls(Neocortex ~ Brain.surf, correlation=corBrownian (1,phy=Sca
_Tree), data=Sca)

Terline_Br_B <-gls(Neocortex ~ Brain.surf, correlation=corBrownian (1,phy=Ter
_Tree), data=Ter)

#Prepare PGLS for each locomotor mode
pgls.fit.Arb <- predict(Arbline_Br_B)
predframe.Arb <- with(Arb, data.frame(Species, Ecology, Brain.surf, Neocortex
= pgls.fit.Arb))

pgls.fit.Fos <- predict(Fosline_Br_B)
predframe.Fos <- with(Fos, data.frame(Species, Ecology, Brain.surf, Neocortex
= pgls.fit.Fos))

pgls.fit.Gli <- predict(Gliline_Br_B)
predframe.Gli <- with(Gli, data.frame(Species, Ecology, Brain.surf, Neocortex
= pgls.fit.Gli))

pgls.fit.Sca <- predict(Scaline_Br_B)
predframe.Sca <- with(Sca, data.frame(Species, Ecology, Brain.surf, Neocortex
= pgls.fit.Sca))

pgls.fit.Ter <- predict(Terline_Br_B)
predframe.Ter <- with(Ter, data.frame(Species, Ecology, Brain.surf, Neocortex
= pgls.fit.Ter))

#Make graph with PGLS corrected regressions
ggplot(squirrel.data, aes(Brain.surf, Neocortex, color = Ecology)) +
  geom_point(data = dplyr::filter(squirrel.data, Ecology == "Arboreal"),
             size = 2, aes(color = "#0EAF28")) +
  geom_point(data = dplyr::filter(squirrel.data, Ecology == "Fossorial"),
```

```
             size = 2, aes(color = "#975822")) +
  geom_point(data = dplyr::filter(squirrel.data, Ecology == "Glider"),
             size = 2, aes(color = "#73DAF3")) +
  geom_point(data = dplyr::filter(squirrel.data, Ecology == "Scansorial"),
             size = 2, aes(color = "#F31616")) +
  geom_point(data = dplyr::filter(squirrel.data, Ecology == "Terrestrial"),
             size = 2, aes(color = "#F3B116")) +
  geom_line(data = dplyr::filter(predframe.Arb, Ecology == "Arboreal"), color
= "#0EAF28",
            linetype = 1.5) +
  geom_line(data = dplyr::filter(predframe.Fos, Ecology == "Fossorial"), colo
r = "#975822",
            linetype = 1.5) +
  geom_line(data = dplyr::filter(predframe.Gli, Ecology == "Glider"), color =
"#73DAF3",
            linetype = 1.5) +
  geom_line(data = dplyr::filter(predframe.Sca, Ecology == "Scansorial"), col
or = "#F31616",
            linetype = 1.5) +
  geom_line(data = dplyr::filter(predframe.Ter, Ecology == "Terrestrial"), co
lor = "#F3B116",
            linetype = 1.5) +
  theme_minimal() +
  #theme(legend.position = "top") +
  scale_color_manual(name = "", values = c("#0EAF28","#73DAF3","#975822", "#F
31616", "#F3B116"),labels = c("Arboreal",
            "Glider","Fossorial", "Scansorial", "Terrestrial")) +
  theme(axis.text = element_text(size = 12), axis.title = element_text(size =
12,
            face = "bold")) +
  labs(x = "log(Endocranial surface area)", y = "log(Neocortical surface area
)") +
  geom_text(data = dplyr::filter(squirrel.data, Ecology == "Arboreal"), color
= "#0EAF28",
            aes(label = abbreviation), hjust = -0.3, vjust = 1.1)+
  geom_text(data = dplyr::filter(squirrel.data, Ecology == "Fossorial"), colo
r = "#975822",
            aes(label = abbreviation), hjust = -0.3, vjust = 1.1) +
  geom_text(data = dplyr::filter(squirrel.data, Ecology == "Glider"), color =
"#73DAF3",
            aes(label = abbreviation), hjust = -0.3, vjust = 1.1) +
  geom_text(data = dplyr::filter(squirrel.data, Ecology == "Scansorial"), col
or = "#F31616",
            aes(label = abbreviation), hjust = -0.3, vjust = 1.1) +
  geom_text(data = dplyr::filter(squirrel.data, Ecology == "Terrestrial"), co
lor = "#F3B116",
            aes(label = abbreviation), hjust = -0.3, vjust = 1.1)
```

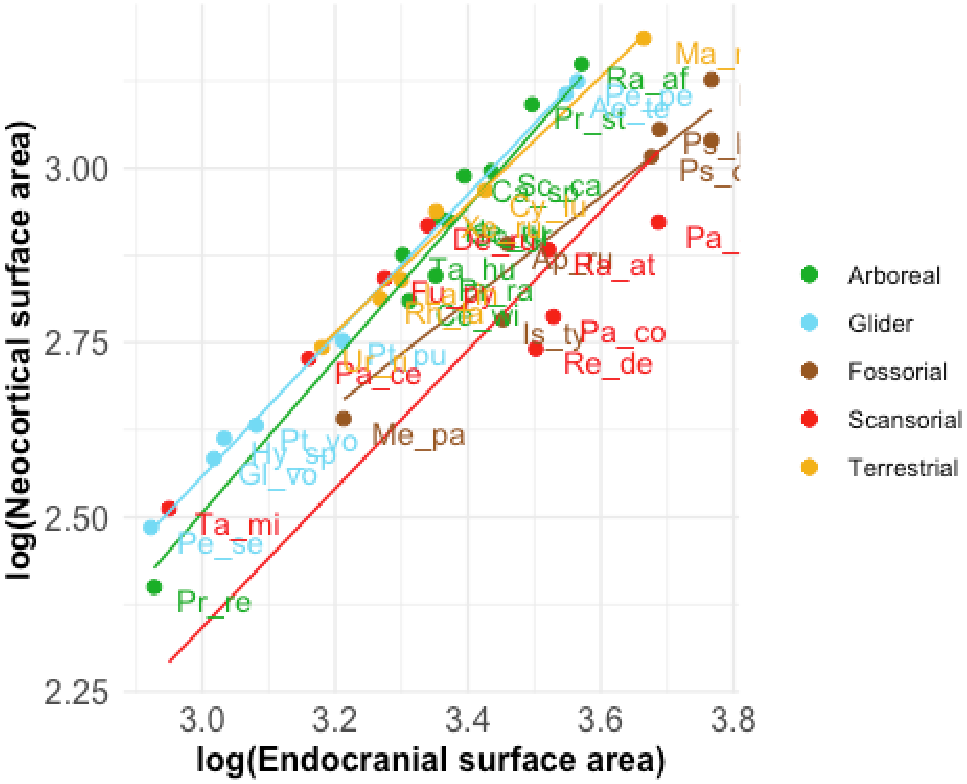

log(Neocortical surface area)
log(Endocranial surface area)
2.25
2.50
2.75
3.00
3.0
3.2
3.4
3.6
3.8
Arboreal
Glider
Fossorial
Scansorial
Terrestrial
Ra_af
Pr_st
Sc_ca
Ta_hu
Is_ty
Pa_co
Re_de
Pa_ce
Pt_vo
Me_pa
Ta_mi
Pr_re

```
# 9. Ancestral state reconstruction

library("ape")
library("strap") #geological scale

## Loading required package: geoscale

library("phytools") #ASR

## Loading required package: maps

#directory
setwd("~/Desktop/Squirrel_June_8_2020/Code")

#Import squirrel data
squirrel.data2<-read.csv("squirrels_PEQ_res.csv", header=T, row.names = 1)

#Import tree
tree_squirrel2<-read.newick("Calibrated_tree_meng")

######## Node numbering ############

### Node number on non-calibrated tree
tree_squirrel<-read.nexus("squirrel-tree_Meng.nex")
plotTree(tree_squirrel,pts=F,node.numbers=T,fsize=0.6)

### Node number on non-calibrated tree - without Petinomys
tree_squirrelEX<-drop.tip(tree_squirrel, c("Petinomys_setosus"))
plotTree(tree_squirrelEX,pts=F,node.numbers=T,fsize=0.6)

####Ancestral state reconstructions - Continous characters

squirrel.data<-squirrel.data2[,5:20] # Meng

for (i in 1:ncol(squirrel.data)){

  dat1<-as.matrix(squirrel.data)[,i]
  dat2<-na.exclude(dat1)#remove Nas
  dat3<-as.numeric(dat2)
  dat3<-setNames(dat3, names(dat2))

  #Drop the missing taxa
  if (anyNA(dat1, recursive = FALSE)==TRUE){
    phy<-drop.tip(tree_squirrel2, names(which(is.na(dat1))))
    anc.eq<-fastAnc(phy,dat3,vars=TRUE,CI=TRUE) # estimate states  with varia
nces & 95% confidence intervals for each node

    num.anc<-anc.eq$ace
    num.anc2<-round(num.anc,digits =2)
    write.table(num.anc2, paste(colnames(squirrel.data)[i],"_ancestral.csv"))
```

```
    #plot ancestral states in the tree

    #Represent ancestral states as colors
    plt<-contMap(phy,dat3, plot=FALSE)
    plt<-setMap(plt, colors=c("black","darkmagenta","violetred2", "orange", "
lightgoldenrod1"))

    # Open a pdf file
    pdf(paste(colnames(squirrel.data[i]),"plot.pdf"),width = 18.27, height =
11.69, paper= "a4r")

    # 2. Create a plot
    plot(plt,legend=FALSE,ylim=c(1-0.09*(Ntip(plt$tree)-1),Ntip(plt$tree)))

    #Bar for ancestral state range
    add.color.bar(leg=0.3*max(nodeHeights(phy)),plt$cols,colnames(squirrel.da
ta[i]),
                  lims=plt$lims,digits=3,prompt=FALSE,x=20,
                  y=1-0.08*(Ntip(plt$tree)-60),lwd=7,fsize=0.8,subtitle="")

    #Bar time scale
    phy$root.time <- 53

    time.tree<- geoscalePhylo(tree=ladderize(phy,right=FALSE), units=c("Perio
d", "Epoch", "Age"), boxes="Epoch",
                              cex.tip=0.8, cex.age=0.8, cex.ts=1.0, label.off
set=0, x.lim=c(0,53), lwd=3, width=2)

    #Label for nodes tree ASR
    nodelabels(num.anc2, cex=0.8, frame="none", adj = c(1.2, -0.3) )

    # Close the pdf file
    dev.off()

    #use a phenogram to represent the phenotypes
    par(mar = c(5.1, 4.1, 4.1, 2.1))

    # Open a pdf file
    pdf(paste(colnames(squirrel.data[i]),"plot2.pdf"),width = 18.27, height =
11.69, paper= "a4r")

    # 2. Create a plot
    phenogram(phy,dat3,fsize=0.6,ftype="i",spread.labels=TRUE, spread.cost=c(
1,0))

    # 2. Create the phenogram plot
    plotTree(phy,pts=F,node.numbers=T,fsize=0.6) #node labels

    # Close the pdf file
    dev.off()
```

```r
  }else {

    anc.eq<-fastAnc(tree_squirrel2,dat3,vars=TRUE,CI=TRUE) # estimate states
with variances & 95% confidence intervals for each node
    num.anc<-anc.eq$ace
    num.anc2<-round(num.anc,digits =2)

    write.table(num.anc2, paste(colnames(squirrel.data)[i],"_ancestral.csv"))

    #plot ancestral states in the tree

    #Represent ancestral states as colors
    plt<-contMap(tree_squirrel2,dat3, plot=FALSE)
    plt<-setMap(plt, colors=c("black","darkmagenta","violetred2", "orange", "
lightgoldenrod1"))

    # Open a pdf file
    pdf(paste(colnames(squirrel.data[i]),"plot.pdf"), width = 18.27, height =
11.69, paper= "a4r")

    # 2. Create a plot
    plot(plt,legend=FALSE,ylim=c(1-0.09*(Ntip(plt$tree)-1),Ntip(plt$tree)))

    #Bar for ancestral state range
    add.color.bar(leg=0.3*max(nodeHeights(tree_squirrel2)),plt$cols,colnames(
squirrel.data[i]),
                  lims=plt$lims,digits=3,prompt=FALSE,x=20,
                  y=1-0.08*(Ntip(plt$tree)-60),lwd=7,fsize=0.8,subtitle="")

    #Bar time scale
    tree_squirrel2$root.time <- 53

    time.tree<- geoscalePhylo(tree=ladderize(tree_squirrel2,right=FALSE), uni
ts=c("Period", "Epoch", "Age"), boxes="Epoch",
                              cex.tip=0.8, cex.age=0.8, cex.ts=1.0, label.off
set=0, x.lim=c(0,53), lwd=3, width=2)

    #Label for nodes tree ASR
    nodelabels(num.anc2, cex=0.8, frame="none", adj = c(1.2, -0.3) )

    # Close the pdf file
    dev.off()

    #use a phenogram to represent the phenotypes
    par(mar = c(5.1, 4.1, 4.1, 2.1))

    # Open a pdf file
    pdf(paste(colnames(squirrel.data[i]),"plot2.pdf"),width = 18.27, height =
11.69,  paper= "a4r")
```

```
    # 2. Create a plot
    phenogram(tree_squirrel2,dat3,fsize=0.6,ftype="i",spread.labels=TRUE, spr
ead.cost=c(1,0))

    # 2. Create the phenogram plot
    plotTree(tree_squirrel2,pts=F,node.numbers=T,fsize=0.6) #node labels

    # Close the pdf file
    dev.off()
  }
}

#Discrete characters: Locomotion

#Import squirrel data
dataloc<-read.csv("squirrels_PEQ_res.csv", header=T, row.names = 1)
loco<-setNames(dataloc[,24],rownames(dataloc))

plotTree(tree_squirrel2,type="phylogram",fsize=0.7,ftype="i",lwd=1)
cols<-setNames(c("chartreuse3", "chocolate4", "steelblue1","firebrick2","yell
ow"),levels(loco))
tiplabels(pie=to.matrix(loco[tree_squirrel2$tip.label],
                        levels(loco)),piecol=cols,cex=0.3)
add.simmap.legend(colors=cols,prompt=FALSE,x=-2*par()$usr[1],
                  y=-40*par()$usr[3],fsize=0.8)
```

```
###### Reconstruction ##########

#MCMC approach -- sample character histories from their posterior probability
distribution.
mtrees<-make.simmap(tree_squirrel2,loco,nsim=1000, model="ER")

## make.simmap is sampling character histories conditioned on
## the transition matrix
##
## Q =
##                Arboreal   Fossorial      Glider  Scansorial Terrestrial
## Arboreal    -0.11951460  0.02987865  0.02987865  0.02987865  0.02987865
## Fossorial    0.02987865 -0.11951460  0.02987865  0.02987865  0.02987865
## Glider       0.02987865  0.02987865 -0.11951460  0.02987865  0.02987865
## Scansorial   0.02987865  0.02987865  0.02987865 -0.11951460  0.02987865
## Terrestrial  0.02987865  0.02987865  0.02987865  0.02987865 -0.11951460
## (estimated using likelihood);
## and (mean) root node prior probabilities
## pi =
##    Arboreal   Fossorial      Glider  Scansorial Terrestrial
##         0.2         0.2         0.2         0.2         0.2
```

```
## Done.

#summarize a set of stochastic maps
pd<-summary(mtrees)
pd

## 1000 trees with a mapped discrete character with states:
##  Arboreal, Fossorial, Glider, Scansorial, Terrestrial
##
## trees have 25.914 changes between states on average
##
## changes are of the following types:
##      Arboreal,Fossorial Arboreal,Glider Arboreal,Scansorial
## x->y              2.599           1.543               1.802
##      Arboreal,Terrestrial Fossorial,Arboreal Fossorial,Glider
## x->y                1.539              1.288            0.688
##      Fossorial,Scansorial Fossorial,Terrestrial Glider,Arboreal
## x->y                0.622                 0.694           1.004
##      Glider,Fossorial Glider,Scansorial Glider,Terrestrial Scansorial,Arbo
real
## x->y            0.752             0.491              0.621
2.35
##      Scansorial,Fossorial Scansorial,Glider Scansorial,Terrestrial
## x->y                1.836             0.907                  0.892
##      Terrestrial,Arboreal Terrestrial,Fossorial Terrestrial,Glider
## x->y                 2.44                 0.795              0.673
##      Terrestrial,Scansorial
## x->y                  2.378
##
## mean total time spent in each state is:
##        Arboreal  Fossorial     Glider Scansorial Terrestrial    total
## raw  74.3375877 40.3752546 29.4715996 41.9831449  29.6391261 215.8067
## prop  0.3444637  0.1870899  0.1365648  0.1945405   0.1373411   1.0000

plot(pd,fsize=0.6,ftype="i",colors=cols,lwd=1,ylim=c(-2,Ntip(tree_squirrel2))
)

add.simmap.legend(colors=cols,prompt=FALSE,x=5,
                  y=8,fsize=0.6)

#### END
```
