# Supplementary material for: The impact of locomotion on the brain evolution of squirrels and close relatives
Source: Commun Biol. 2021 Apr 12;4:460. doi: 10.1038/s42003-021-01887-8 (PMC8042109; doi:10.1038/s42003-021-01887-8)
Supplement: Supplementary file 3 — Reporting Summary [file 42003_2021_1887_MOESM3_ESM.pdf]

nature research

Corresponding author(s): Ornella C. Bertrand

Last updated by author(s): Feb 10, 2021

Reporting Summary

Nature Research wishes to improve the reproducibility of the work that we publish. This form provides structure for consistency and transparency in reporting. For further information on Nature Research policies, see our Editorial Policies and the Editorial Policy Checklist.

Statistics

For all statistical analyses, confirm that the following items are present in the figure legend, table legend, main text, or Methods section.

n/a	Confirmed	
☐	☒	The exact sample size (*n*) for each experimental group/condition, given as a discrete number and unit of measurement
☐	☒	A statement on whether measurements were taken from distinct samples or whether the same sample was measured repeatedly
☐	☒	The statistical test(s) used AND whether they are one- or two-sided<br>*Only common tests should be described solely by name; describe more complex techniques in the Methods section.*
☐	☒	A description of all covariates tested
☐	☒	A description of any assumptions or corrections, such as tests of normality and adjustment for multiple comparisons
☐	☒	A full description of the statistical parameters including central tendency (e.g. means) or other basic estimates (e.g. regression coefficient) AND variation (e.g. standard deviation) or associated estimates of uncertainty (e.g. confidence intervals)
☐	☒	For null hypothesis testing, the test statistic (e.g. *F*, *t*, *r*) with confidence intervals, effect sizes, degrees of freedom and *P* value noted<br>*Give P values as exact values whenever suitable.*
☒	☐	For Bayesian analysis, information on the choice of priors and Markov chain Monte Carlo settings
☐	☒	For hierarchical and complex designs, identification of the appropriate level for tests and full reporting of outcomes
☒	☐	Estimates of effect sizes (e.g. Cohen's *d*, Pearson's *r*), indicating how they were calculated

Our web collection on statistics for biologists contains articles on many of the points above.

Software and code

Policy information about availability of computer code

Data collection	The endocranial data were collected with the software Avizo Lite 2019.4.
Data analysis	We used the statistical software R (version 3.6.2) and R Studio (version 1.2.5) to conduct the different analyses. The code used to conduct the different analyses is available in the code section of the supplementary data and at the following GitHub repository: https://github.com/Bertrand-Ornella/Brain-evolution-Sciuroidea.

For manuscripts utilizing custom algorithms or software that are central to the research but not yet described in published literature, software must be made available to editors and reviewers. We strongly encourage code deposition in a community repository (e.g. GitHub). See the Nature Research guidelines for submitting code & software for further information.

Data

Policy information about availability of data

All manuscripts must include a data availability statement. This statement should provide the following information, where applicable:

- Accession codes, unique identifiers, or web links for publicly available datasets
- A list of figures that have associated raw data
- A description of any restrictions on data availability

Previously published endocasts, the new surface rendering of the endocast of Reithroparamys delicatissimus and those of the three extant terrestrial squirrels generated for this project are available in MorphoSource (www.morphosource.org) at https://www.morphosource.org/Detail/ProjectDetail/Show/project_id/83.

Field-specific reporting

Please select the one below that is the best fit for your research. If you are not sure, read the appropriate sections before making your selection.

☐ Life sciences ☐ Behavioural & social sciences ☒ Ecological, evolutionary & environmental sciences

For a reference copy of the document with all sections, see nature.com/documents/nr-reporting-summary-flat.pdf

Ecological, evolutionary & environmental sciences study design

All studies must disclose on these points even when the disclosure is negative.

Study description	The goals of this study were to test the impact of locomotion, body mass and phylogeny on brain size and its components and to investigate the sequence of neurological changes that occurred over time in Sciuroidea.
Research sample	We built a dataset of 42 virtual cranial endocasts from 38 species of rodents: 25 extant and 13 extinct. Our fossil sample spans the early Eocene to the early Miocene, and incorporates all well-preserved crania accessible to us, which cover a breadth of the temporal, habitat, and locomotor diversity of these animals. These include 12 endocasts of well-preserved crania of ischyromyids. The sample includes 6 different species of the basal Paramyinae from the genera Paramys (2 species) and Pseudotomus (4 species) and three more derived taxa: Reithroparamys delicatissimus, Rapamys atramontis (N=2) (Reithroparamyinae), and Ischyromys typus (N=3; Ischyromyinae). Our sciuroid fossil sample includes basal taxa (the squirrel Cedromus wilsoni and aplodontiid Prosciurus relictus) and more derived species (the squirrel Protosciurus rachelae [N=2] and aplodontiid Mesogaulus paniensis). Our extant sample includes 24 endocasts of squirrels and one of Aplodontia rufa (the mountain beaver). This sample includes members of all extant squirrel subfamilies except the monospecific subfamily Sciurillinae. Our endocasts include 10 Sciurinae (tree and flying squirrels), 4 Callosciurinae (Asian squirrels), 1 Ratufinae (Ratufa affinis), and 9 Xerinae (ground squirrels and African tree squirrels) species, thus spanning a range of habitats and ecologies.
Sampling strategy	We gathered data that were already available and added three new virtual endocasts. There are additional taxa that potentially could be sampled in the future, but these were not available to us, and it is unclear if they are well enough preserved for CT scanning. With that said, our sample incorporates taxa that represent diverse locomotor conditions in both Sciuridae and Aplodontiidae, across a timeframe of 36 million years, which should provide an adequate framework for testing the effects of locomotor transitions on the brain through time.
Data collection	The data including the volume and surface of the brain and different regions of the brain were collected in Avizo by Ornella Bertrand (First author). The body mass was obtained using a published equation (Bertrand et al., 2016).<br><br>Bertrand OC, Schillaci MA, Silcox MT (2016) Cranial dimensions as estimators of body mass and locomotor habits in extant and fossil rodents. J Vert Paleontol 36 (1). doi:10.1080/02724634.2015.1014905
Timing and spatial scale	The data were collected on fossil and modern museum specimens.
Data exclusions	No data were excluded from the analyses.
Reproducibility	All the analyses can be reproduced using the data and code described in the supplementary information.
Randomization	To investigate the role of locomotion types on endocranial size and the size of brain components, we performed a non-phylogenetic ANOVA based on the Residual Randomization in R using the function “lm.rrpp” and ran post-hoc tests with the function “pairwise”, which are both from the package RRPP (version 0.6.1).
Blinding	Blinding is not relevant for our study as we were working with museum specimens.

Did the study involve field work? ☐ Yes ☒ No

Reporting for specific materials, systems and methods

We require information from authors about some types of materials, experimental systems and methods used in many studies. Here, indicate whether each material, system or method listed is relevant to your study. If you are not sure if a list item applies to your research, read the appropriate section before selecting a response.

Materials & experimental systems

n/a	Involved in the study
☒	☐ Antibodies
☒	☐ Eukaryotic cell lines
☐	☒ Palaeontology and archaeology
☒	☐ Animals and other organisms
☒	☐ Human research participants
☒	☐ Clinical data
☒	☐ Dual use research of concern

Methods

n/a	Involved in the study
☒	☐ ChIP-seq
☒	☐ Flow cytometry
☒	☐ MRI-based neuroimaging

Palaeontology and Archaeology

Specimen provenance

The majority of the virtual endocasts have already been published in the following papers: Bertrand and Silcox, 2016; Bertrand et al., 2016; 2017; 2018; 2019a; 2019b.

-Bertrand OC, Silcox MT (2016) First virtual endocasts of a fossil rodent: Ischyromys typus (Ischyromyidae, Oligocene) and brain evolution in rodents. J Vert Paleontol 36 (3). doi:10.1080/02724634.2016.1095762

-Bertrand OC, Amador-Mughal F, Silcox MT (2016) Virtual endocasts of Eocene Paramys (Paramyinae): oldest endocranial record for Rodentia and early brain evolution in Euarchontoglires. Proc Biol Sci 283 (1823). doi:10.1098/rspb.2015.2316

-Bertrand OC, Amador-Mughal F, Silcox MT (2017) Virtual endocast of the early Oligocene Cedromus wilsoni (Cedromurinae) and brain evolution in squirrels. J Anat 230 (1):128-151. doi:10.1111/joa.12537

-Bertrand OC, Amador-Mughal F, Lang MM, Silcox MT (2018) Virtual endocasts of fossil Sciuroidea: brain size reduction in the evolution of fossoriality. Palaeontology 61 (6):919-948. doi:10.1111/pala.12378

-Bertrand OC, Amador-Mughal F, Lang MM, Silcox MT (2019a) New Virtual Endocasts of Eocene Ischyromyidae and Their Relevance in Evaluating Neurological Changes Occurring Through Time in Rodentia. J Mamm Evol 26 (3):345-371. doi:10.1007/s10914-017-9425-6

-Bertrand OC, San Martin-Flores G, Silcox MT (2019b) Endocranial shape variation in the squirrel-related clade and their fossil relatives using 3D geometric morphometrics: contributions of locomotion and phylogeny to brain shape. J Zool 308 (3):197-211. doi:10.1111/jzo.12665

The newly described specimens were scanned at McGill University in Montreal (Quebec) at the Integrative Quantitative Biology Initiative (McGill University, QB). The specimens belong to the American Museum of Natural History (AMNH, New York).

Specimen deposition

All the virtual endocasts used for the study have been deposited in MorphoSource (www.morphosource.org) at https://www.morphosource.org/Detail/ProjectDetail/Show/project_id/83.

Dating methods

No new dates are provided.

☐ Tick this box to confirm that the raw and calibrated dates are available in the paper or in Supplementary Information.

Ethics oversight

No ethical approval or guidance was required for this study as we used 3D model of the brain of fossil and modern museum specimens.

Note that full information on the approval of the study protocol must also be provided in the manuscript.
